# Temperature Exposure and Psychiatric Symptoms in Adolescents From 2 European Birth Cohorts

**DOI:** 10.1001/jamanetworkopen.2024.56898

**Published:** 2025-01-28

**Authors:** Esmée Essers, Michelle Kusters, Laura Granés, Joan Ballester, Sami Petricola, Nerea Lertxundi, Ane Arregi, Ferran Ballester, Martine Vrijheid, Hanan El Marroun, Carmen Iñiguez, Henning Tiemeier, Mònica Guxens

**Affiliations:** 1 ISGlobal, Barcelona, Spain; 2 Department of Medicine and Life Sciences, Universitat Pompeu Fabra, Barcelona, Spain; 3 Department of Child and Adolescent Psychiatry and Psychology, Erasmus University Medical Centre, Rotterdam, the Netherlands; 4 Spanish Consortium for Research on Epidemiology and Public Health, Instituto de Salud Carlos III, Madrid, Spain; 5 Department of Psychiatry, Bellvitge University Hospital, Bellvitge Biomedical Research Institute, Barcelona, Spain; 6 Environmental Epidemiology and Child Development Group, Biogipuzkoa Health Research Institute, San Sebastian, Spain; 7 Faculty of Psychology, University of the Basque Country, San Sebastian, Spain; 8 Department of Nursing, Universitat de València, Valencia, Spain; 9 Department of Psychology, Education and Child Studies, Erasmus School of Social and Behavioural Sciences, Erasmus University Rotterdam, Rotterdam, the Netherlands; 10 Department of Statistics and Operational Research, Universitat de València, Valencia, Spain; 11 Department of Social and Behavioural Sciences, Harvard T.H. Chan School of Public Health, Boston; 12 ICREA, Barcelona, Spain

## Abstract

**Importance:**

Climate change can adversely affect mental health, but the association of ambient temperature with psychiatric symptoms remains poorly understood.

**Objective:**

To assess the association of ambient temperature exposure with internalizing, externalizing, and attention problems in adolescents from 2 population-based birth cohorts in Europe.

**Design, Setting, and Participants:**

This cohort study analyzed data from the Dutch Generation R Study and the Spanish INMA (Infancia y Medio Ambiente) Project. Generation R recruited 9898 women during pregnancy or shortly after birth, with children born between 2002 and 2006. INMA recruited 2270 pregnant women from Gipuzkoa, Sabadell, and Valencia, Spain, with children born between 2003 and 2008. Individuals born from live singleton births with available outcome and exposure data were included in the study. Data were analyzed between October 2023 and November 2024.

**Exposure:**

Daily ambient temperature 2 weeks, 1 month, and 2 months preceding outcome assessment was calculated between December 2015 and November 2022 at the residence at 100 × 100 m resolution utilizing the UrbClim model.

**Main Outcomes and Measures:**

The primary outcomes were internalizing, externalizing, and attention problems, measured with the maternal-reported Child Behavioral Checklist for ages 6 to 18 years; raw scores were square-root transformed, with higher scores indicating more problems. Distributed lag nonlinear models evaluated the associations of temperature exposure with problem scores in each country and region. For Spain, results from the 3 INMA regions were combined using random-effects meta-analysis. Results show the accumulated temperature association over each exposure period.

**Results:**

A total of 3934 participants from Generation R (mean [SD] age at assessment, 13.6 [0.4] years; 1971 female [50%]) and 885 from INMA (mean [SD] age at assessment, 14.9 [1.0] years; 458 female [52%]) were included. Most parents in both cohorts were native to the respective countries of each cohort and had relatively high socioeconomic status. Daily temperatures ranged from −5.2 °C to 32.6 °C in the Netherlands and 3.3 °C to 33.9 °C in Spain. In Generation R, the mean (SD) square-root transformed scores were 2.0 (1.2) for internalizing problems, 1.6 (1.3) for externalizing problems, and 1.5 (1.0) for attention problems, while in INMA these were 2.4 (1.2), 2.1 (1.3), and 1.5 (1.1), respectively. In the Netherlands, cumulative exposure to cold was associated with more internalizing problems (eg, 0.76 [95% CI, 0.20-1.32] higher square-root points at 5.5 °C exposure over a 2-month exposure). In Spain, cumulative exposure to heat was associated with more attention problems (eg, 1.52 [95% CI, 0.39-2.66] higher square-root points at 21.7 °C exposure over a 2-month exposure).

**Conclusions and Relevance:**

In this cohort study, exposure to cold in the Netherlands and heat in Spain were associated with more psychiatric symptoms, highlighting distinct temperature exposure and mental health associations among adolescents. Future studies should explore this across diverse climates to further quantify the intricate and multifactorial association of climate change with mental health.

## Key Points

**Question:**

Is ambient temperature exposure associated with internalizing, externalizing, and attention problems in adolescents from 2 European birth cohorts?

**Findings:**

In this cohort study evaluating 3934 adolescents in the Netherlands and 885 adolescents in Spain between 2015 and 2022, cold exposure was associated with more internalizing problems in the Netherlands, and heat exposure was associated with more attention problems in Spain.

**Meaning:**

This cohort study found that ambient temperature has distinct associations with psychiatric symptoms in adolescents from 2 European countries, highlighting the importance of conducting further research across diverse climates to further quantify the intricate and multifactorial association of climate change with mental health.

## Teaser

This cohort study explores associations of ambient temperature exposure with internalizing, externalizing, and attention problems among adolescents from the Netherlands and Spain.

## Introduction

Human-induced climate change has profoundly impacted the natural world and human population, disregarding geographical or physiological boundaries.^1^ Increasingly frequent and intense extreme temperatures threaten human health.^2^ While the association of temperature with physical health risks like mortality and morbidity is well-established,^3,4^ research on associations with mental health remains less explored, although evidence is growing.^5,6^ Cold or heat exposure disrupts thermoregulation and cell function, leading to changes in blood pressure, inflammation, or impaired brain cooling and oxygenation.^7,8,9^ Thermal discomfort is also known to trigger irritability or decrease happiness.^10,11^

Previous studies have predominantly examined end points such as mental health–related hospitalization or suicide risk,^12,13,14,15,16,17^ and have focused less on psychiatric symptoms preceding these end points.^18,19,20^ Evaluating symptoms like internalizing, externalizing, and attention problems provides insight into the psychiatric disorder continuum.^21^ Previous studies have found associations of higher temperatures with more externalizing problems in preadolescents, adolescents, and young adults aged 9 to 18 years in California,^20^ more depressive symptoms in individuals aged 45 years and older in China,^19^ and poorer emotional well-being in individuals aged 18 years and older in the US.^18^ However, these studies primarily examined heat exposure while disregarding cold, and failed to consider the delayed effect of temperature exposure.^18,19,20^ Also, prior research mostly examined adults, overlooking the vulnerable population of adolescents and young adults, even though approximately 1 in 7 youths aged 10 to 19 years experience psychiatric disorders.^22^ Considering the projected increase in frequency and intensity of extreme temperatures, more research is needed to elucidate how climate-related indicators impact mental health during this developmental stage.^5,23^ We aim to evaluate the nonlinear delayed associations of ambient temperature with internalizing, externalizing, and attention problems in adolescents from 2 European birth cohorts with varying climates.

## Methods

### Population and Study Design

This cohort study utilized data from 2 European population-based birth cohorts: the Dutch Generation R Study and the Spanish INMA (Infancia y Medio Ambiente) Project. The Generation R Study, based in Rotterdam, the Netherlands, recruited 9898 women during pregnancy or shortly after birth, with children born between 2002 and 2006.^24^ The INMA Project, based in Spain, recruited pregnant women across multiple Spanish regions between 1997 and 2008. Based on data availability, we included the Gipuzkoa (638 participants), Sabadell (657 participants), and Valencia (855 participants) regions, with children born between 2003 and 2008.^25^ An additional 120 mother-child pairs from Sabadell were included shortly after birth. We included adolescents from live singleton births with at least 1 available score for internalizing, externalizing, or attention problems, and complete daily temperature data up to 2 months before the outcome assessment (eFigure 1 in Supplement 1). Ethical approval was granted by the Medical Ethical Committee of Erasmus University Medical Centre Rotterdam for Generation R and the Ethical Committee of the Municipal Institute of Medical Investigation and the Ethical Committees of the hospitals involved for INMA. Written informed consent was obtained from parents and adolescents. We followed the Strengthening the Reporting of Observational Studies in Epidemiology (STROBE) reporting guideline.

### Temperature Exposure

The urban climate model UrbClim was used to calculate ambient temperature exposure between December 2015 and November 2022.^26^ UrbClim uses urban physics and urban structure information to estimate hourly 2-m temperature (°C) at a horizontal resolution of 100 × 100 m (eAppendix 1 in Supplement 1). This temperature data has been previously used for Generation R and INMA.^27^ We calculated daily temperature data from the 1st day to the 56th day (2 months) preceding outcome assessment. Using daily gridded observational data for European temperatures from ENSEMBLES Observations Gridded Dataset,^28^ we validated UrbClim daily temperature data from 2002 to 2022 and found high performance (average multiple *R*^2^ of 0.98; average root mean squared error of 1.6 °C across cohorts and regions).

### Psychiatric Symptoms

Internalizing, externalizing, and attention problems were assessed using the maternal-reported Child Behavior Checklist for ages 6 to 18 years (CBCL/6-18) in both cohorts.^21^ Ages of participants at time of assessment were between 12.6 and 16.6 years for Generation R and between 12.6 and 17.5 years for INMA. The CBCL/6-18 contains 112 items evaluated on a 3-point Likert scale asking about problems in the past 6 months. We used the problem scales for internalizing (32 items from the anxious or depressed, withdrawn or depressed, and somatic complaints scales), externalizing (35 items from the aggressive behavior and rule-breaking behavior scales), and attention (10 items). Theoretical problem scores ranges are 0 to 64 for internalizing, 0 to 70 for externalizing, and 0 to 20 for attention problems. Scores were square-root transformed to achieve linear regression assumptions, resulting in ranges of 0 to 8.0 for internalizing problems, 0 to 8.4 for externalizing problems, and 0 to 4.5 for attention problems, with higher values indicating more problems (eTable 1 in Supplement 1).

### Potential Confounding Variables

Using a directed acyclic graph based on data availability, prior research, and biological plausibility,^18,20,29^ we selected and harmonized potential confounding variables across cohorts (eFigure 2 in Supplement 1). Information included adolescent sex (female or male), parental age at enrollment (years), and national origin (the Netherlands, Suriname, Turkey, Morocco, other European, and other non-European in Generation R and Spain or other in INMA). Variables at the time of (or as close as possible to) the outcome assessment included adolescent age (years), parental education level (low, medium, or high), number of children in the household (1, 2, or ≥3), monthly household income (low, low-medium, medium-high, or high), family status (dual vs single parent), and parental employment status (paid vs unpaid job) (eTable 2 in Supplement 1). Greenness exposure at the residential address was determined using the Normalized Difference Vegetation Index, which uses Landsat satellite data to quantify the difference in red light absorbance by vegetation, yielding values from −1 to 1, with higher values indicating more greenness.^30^ In Generation R, neighborhood socioeconomic status of the residential address during the outcome assessment year was described in terms of wealth, education level, and labor market position of all households within the postal code using Statistics Netherlands data.^31^ In INMA, it was determined using a 2011 deprivation index, which combines 6 socioeconomic indicators.^32^ Seasonality was accounted for by including the outcome assessment month. Air pollution exposure was not included because we considered it a potential mediator on the pathway between temperature and the outcome (eFigure 2 in Supplement 1).^33^

### Statistical Analyses

Missing values of potential confounding variables and problem scores were imputed in Generation R and INMA analysis samples (eFigure 1 in Supplement 1) separately using the expectation-maximization procedure to obtain 1 imputed dataset (eTable 3 in Supplement 1).^34^ Problem scores were imputed for 20 participants in Generation R and missing values for other variables were less than 30% (eTable 4 in Supplement 1). After imputation, all cohorts or regions showed comparable distributions of variables between imputed and observed datasets (eTable 4 in Supplement 1). Population characteristics differed between those included or excluded in the analysis due to follow-up loss or missing CBCL/6-18 or temperature data (eTable 5 in Supplement 1). To mitigate potential selection bias, inverse probability weighting was performed. A weight per participant was calculated by estimating the inverse probability of participation in this study using the covariate balancing propensity score procedure.^35^ Variables included in this procedure and final weight distributions for each participant in each cohort or region are detailed in eTable 6 and eFigure 3 in Supplement 1.

To capture the nonlinear and delayed associations of temperature with each problem score, cohort- and region-specific distributed lag nonlinear models (DLNM) were applied.^36^ DLNM estimates the association of exposure with response across various time lags, enabling the capture of immediate and delayed associations.^37^ To derive results for Spain, we pooled results from the region-specific DLNM of the 3 INMA regions using random-effects meta-analysis fitted through restricted maximum likelihood.^38^ We evaluated 2-week, 1-month, and 2-month lag periods prior to the CBCL/6-18 assessment separately. Because no prior research, to our knowledge, has used DLNM to assess the association of ambient temperature with psychiatric symptoms, these periods were selected a priori to capture current symptoms while accounting for delayed cold temperature effects.^3,39^ After testing model parameterization and evaluating model fit, the association of exposure with response was modeled using natural cubic splines with knots at the 25th and 75th percentiles of temperature distribution across the lag period, and the association of lag with response was modeled linearly (eAppendix 2 in Supplement 1). The temperatures where the lowest adverse associations were observed for each outcome were calculated per cohort and region and lag period, averaged to country-level values for Generation R or INMA, and used as DLNM reference temperatures (eAppendix 2 and eTable 7 in Supplement 1). Final country-level DLNM estimates for each outcome are presented as square-root problem scores and 95% CIs, reflecting the accumulated association of temperature exposure over the lag period. Models were adjusted for the aforementioned potential confounding variables. We corrected for multiple testing on the outcome through identifying 2 effective number of tests using eigenvalues (based on 3 outcomes),^40^ adjusting the statistical significance level to *P* < .025 (.05/2).

We performed follow-up analyses in Generation R (due to data availability), aiming to evaluate potential reporter bias. Because we observed an association for internalizing problems, we evaluated the association of temperature exposure with adolescent-reported internalizing problems from the Youth Self-Report questionnaire^21^ administered concurrently with the CBCL/6-18, and maternal anxiety and depressive symptoms from the Brief Symptom Inventory collected at child age 9 years.^41^ As a sensitivity analysis in INMA (due to data availability), we adjusted the models for household air conditioning or heating. Additionally, we stratified significant main analysis associations by participants living in households with low (below median) vs high (above median) neighborhood socioeconomic status. To evaluate even shorter-term exposure, we tested a 3-day lag period. Finally, we tested various DLNM parameterizations: an association of exposure with response with knots at the 10th and 90th percentiles of the temperature distribution and an association of lag with response modeled with a natural cubic spline with an intercept and 1 knot at the median lag on the log-scale, 1 knot at the median lag equally spaced, and 2 knots equally spaced.

Analyses were done using R version 4.0.3 (R Project for Statistical Computing), using packages Amelia version 1.8.1, CBPS version 0.25, dlnm version 2.4.7, and mixmeta version 1.2.0. Analyses were conducted from October 2023 to November 2024.

## Results

**Figure 1.**
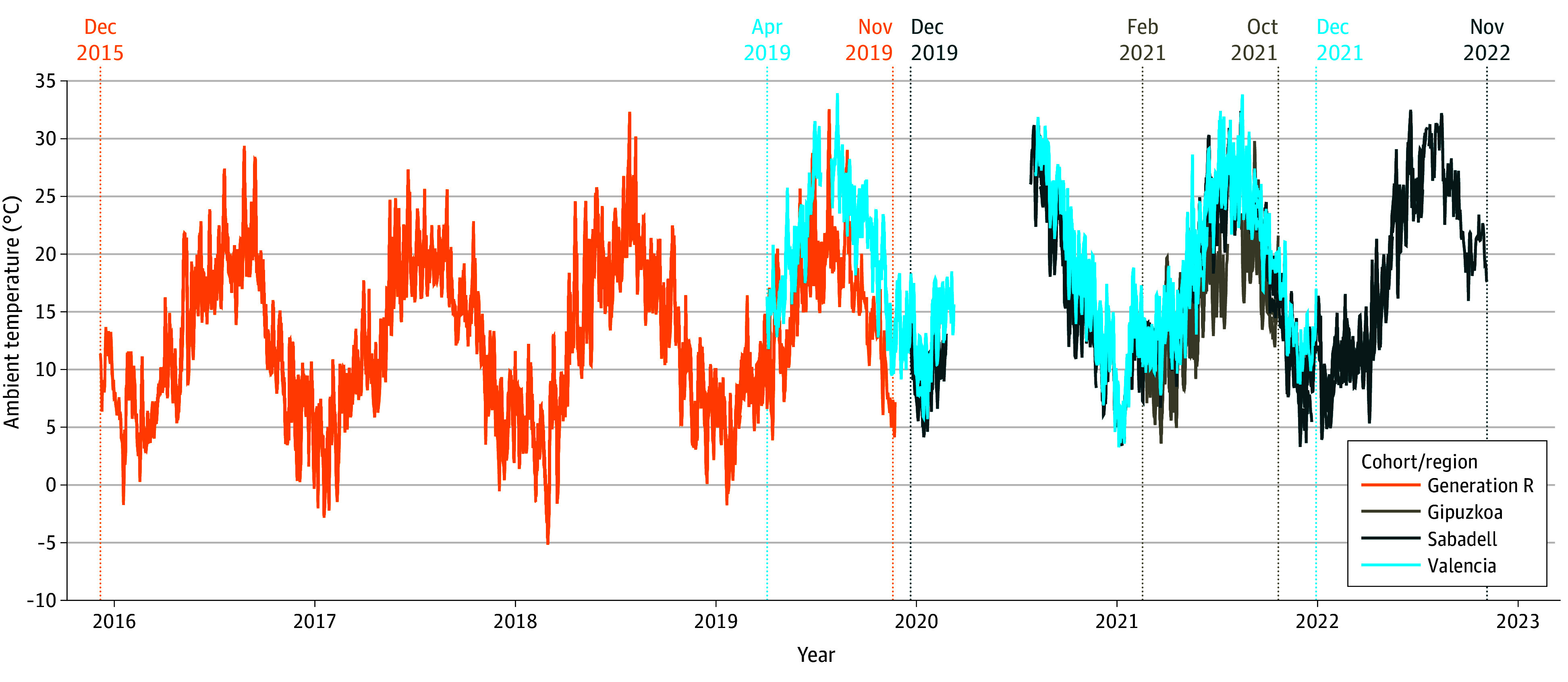
Daily Temperature in 2 European Birth Cohort Populations During the 2 Months Before Outcome Assessment The month and year at the top of the plot indicate the first and last months of Child Behavioral Checklist for ages 6 to 18 years assessment within each cohort or region. Gipuzkoa, Sabadell, and Valencia comprise the Spanish INMA (Infancia y Medio Ambiente) Project.

There were 4819 individuals included in the study, including 3934 from Generation R cohort (mean [SD] age at assessment, 13.6 [0.4] years; 1971 female [50%]) and 885 from INMA (mean [SD] age at assessment, 14.9 [1.0] years; 458 female [52%]) (Table). For both cohorts, the majority of parents were from the country of the cohort and the overall parental socioeconomic status was relatively high, indicated by the high education level, paid job status, and medium-high income level. The temporal pattern for the daily ambient temperatures ranged from −5.2 °C to 32.6 °C in Generation R and 3.3 °C to 33.9 °C in INMA during the 2-month exposure period prior to the CBCL/6-18 assessment (Figure 1). In Generation R, the mean (SD) square-root transformed scores were 2.0 (1.2) for internalizing problems, 1.6 (1.3) for externalizing problems, and 1.5 (1.0) for attention problems, while in INMA these were 2.4 (1.2), 2.1 (1.3), and 1.5 (1.1), respectively (eTable 1 in Supplement 1).

**Table.** Population Characteristics of the 2 European Birth Cohorts

Characteristic	Participants, No. (%) (N = 4789)	
	Generation R (n = 3934)	INMA (n = 855)
Participant characteristics		
Sex		
Female	1971 (50)	458 (52)
Male	1963 (50)	397 (48)
Age at CBCL/6-18 assessment, mean (SD), y	14 (<1)	15 (1)
Season of CBCL/6-18 assessment		
Summer	1070 (27)	100 (11)
Fall	893 (23)	206 (23)
Winter	1090 (28)	165 (19)
Spring	881 (22)	414 (47)
CBCL/6-18 score, median (IQR)^a^		
Internalizing problems	4 (1 to 8)	6 (3 to 11)
Externalizing problems	2 (0 to 6)	4 (1 to 9)
Attention problems	3 (1 to 5)	2 (1 to 6)
Maternal characteristics		
Age at enrollment, mean (SD), y	31 (5)	31 (4)
National origin^b^		
Country of cohort	2351 (60)	835 (95)
Suriname	274 (7)	NA
Turkey	201 (5)	NA
Morocco	168 (4)	NA
Other European	309 (8)	NA
Other non-European	587 (15)	NA
Other	NA	45 (5)
Educational level		
High	2123 (60)	358 (41)
Medium	1046 (30)	339 (39)
Low	337 (10)	170 (20)
Employment status (paid vs unpaid job)	3084 (82)	755 (86)
Paternal characteristics		
Age at enrollment, mean (SD), years	34 (6)	33 (5)
National origin^b^		
Country of cohort	2388 (63)	825 (93)
Suriname	245 (7)	NA
Turkey	199 (5)	NA
Morocco	172 (5)	NA
Other European	195 (5)	NA
Other non-European	564 (15)	NA
Other	NA	58 (7)
Educational level		
High	1964 (61)	221 (27)
Medium	855 (26)	342 (42)
Low	429 (13)	258 (31)
Employment status (paid vs unpaid job)	3263 (91)	825 (93)
Residential characteristics		
Residential surrounding greenness score, mean (SD)^c^	0.3 (0.1)	0.3 (0.2)
Neighborhood socioeconomic status, median (IQR)^d^	−0.1 (−0.2 to 0.1)	−0.5 (−0.9 to −0.0)
Family status (dual vs single parent)	3119 (80)	769 (88)
Monthly household income		
Low	621 (18)	98 (13)
Low-medium	966 (27)	150 (20)
Medium-high	1289 (36)	291 (38)
High	658 (19)	222 (29)
No. of children in the household		
1	634 (18)	136 (16)
2	1912 (56)	588 (69)
≥3	879 (26)	125 (15)

Abbreviations: CBCL/6-18, Child Behavioral Checklist for ages 6 to 18 years; INMA, Infancia y Medio Ambiente; NA, not available.

^a^ Raw score theoretical ranges are 0 to 64, 0 to 70, and 0 to 20 for the internalizing, externalizing, and attention problems, respectively.

^b^ For the Generation R Study, national origin was categorized as the country of the cohort (ie, the Netherlands), Suriname, Turkey, Morocco, other European, and other non-European, while for the INMA Project, national origin was categorized as country of the cohort (ie, Spain) and other (ie, any other country).

^c^ Normalized Difference Vegetation Index; scores range from −1 to 1 with higher values indicating more greenness.

^d^ Range, −0.37 to 0.31 in Generation R and −2.45 to 2.79 in INMA.

**Figure 2.**
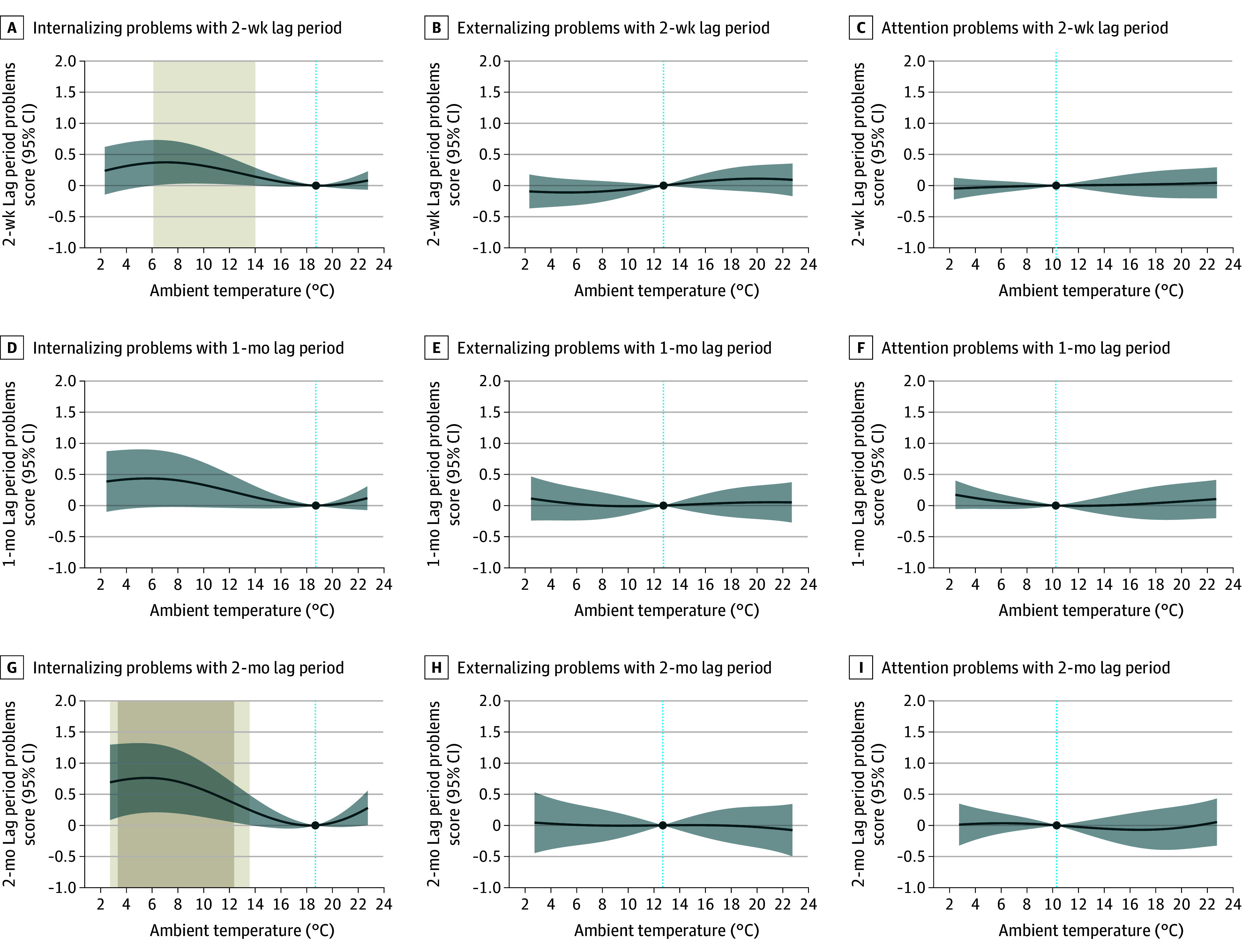
Cumulative Associations of Temperature Exposure Before Outcome Assessment With Psychiatric Symptoms in Adolescents From the Generation R Study The x-axis depicts the 5th to 95th percentile range of the country-wide temperature distribution (2.3°C to 22.8 °C for the 2-week lag period, 2.5°C to 22.7 °C for the 1-month lag period, and 2.7 °C to 22.8 °C for the 2-month lag period). Solid dark blue lines represent the country-average curve, expressed as β coefficients of the problem scores, derived from the distributed lag nonlinear models, with their respective 95% CIs shaded in light blue; the dark blue dot and light blue dotted line in each plot represents the reference temperature. Coefficients are estimated as the change in the square-root transformed outcome score at each temperature of the country-wide temperature distribution respective to the corresponding reference temperature. Light beige (colder compared with reference temperature) shaded areas indicate the range of statistically significant associations at the *P* < .05 level and dark beige shaded areas indicate statistically significant associations after correction for multiple testing (*P* < .025).

In Generation R, colder temperatures were associated with more internalizing problems, but only cumulative exposure between 3.3 °C and 12.4 °C (6th-50th percentile) during the 2 months prior to the outcome assessment survived correction for multiple testing (Figure 2G and eTable 8 in Supplement 1). For example, the estimated cumulative 2-month cold exposure of 5.5 °C (14th percentile) was associated with a 0.76 (95% CI, 0.20-1.32) higher square-root transformed internalizing problems score (corresponding to a raw score of 0.58). Associations revealed similar curves in adolescents living in low or high socioeconomic status neighborhoods, although estimates were larger in the latter (eFigure 4 in Supplement 1). No associations were found for externalizing or attention problems or for heat in Generation R.

**Figure 3.**
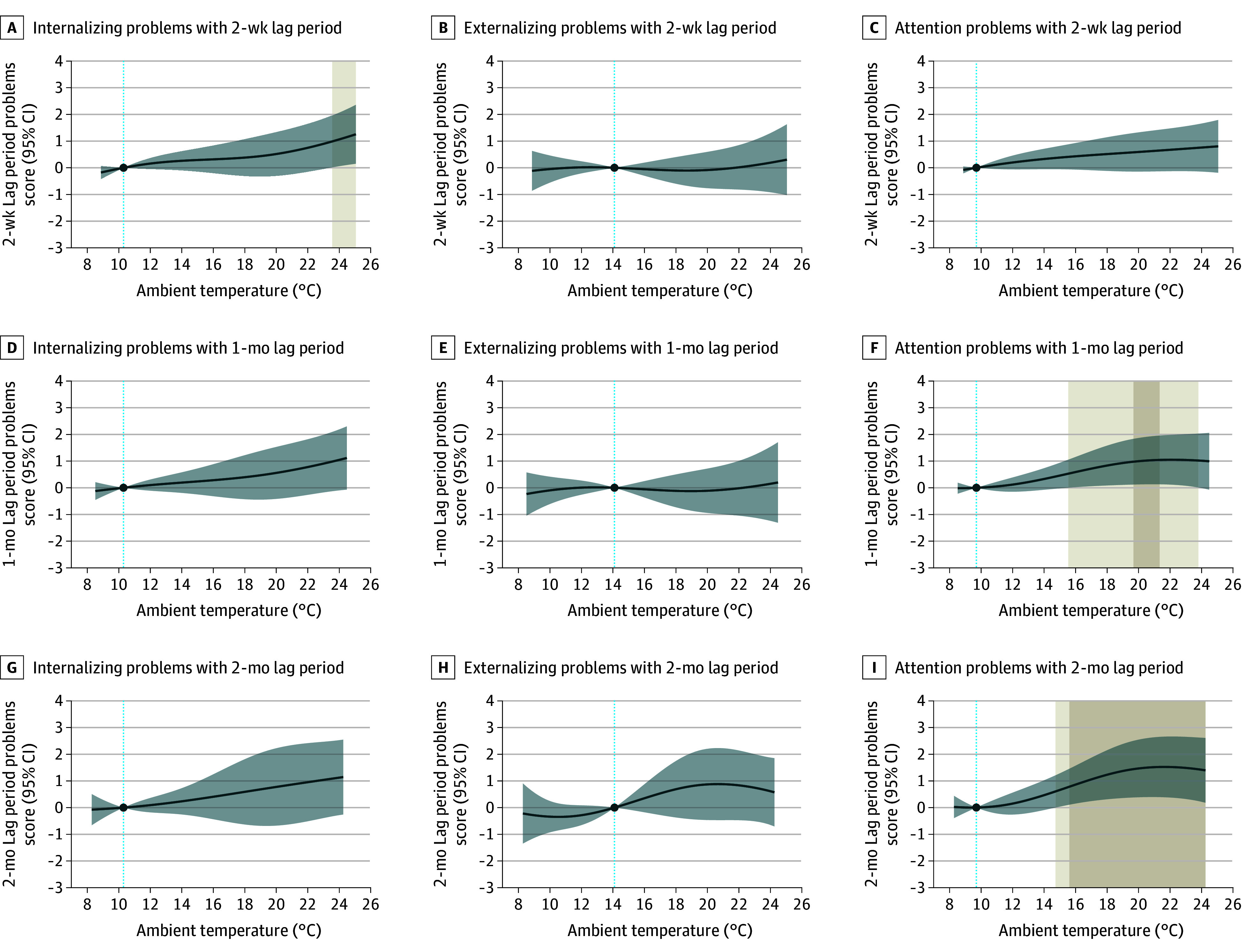
Cumulative Associations of Temperature Exposure Before Outcome Assessment With Psychiatric Symptoms in Adolescents From the INMA (Infancia y Medio Ambiente) Project The x-axis depicts the 5th to 95th percentile range of the country-wide temperature distribution (8.9 °C to 25.0 °C for the 2-week lag period, 8.5 °C to 24.5 °C for the 1-month lag period, and 8.0 °C to 24.3 °C for the 2-month lag period). Solid dark blue lines represent the country-average curve, expressed as β coefficients of the problem scores, derived from the random-effects meta-analysis models, with their respective 95% CIs shaded in light blue; the dark blue dot and light blue dotted line in each plot represents the reference temperature. Coefficients are estimated as the change in the square-root transformed outcome score at each temperature of the country-wide temperature distribution respective to the corresponding reference temperature. Light beige (warmer compared with reference temperature) shaded areas indicate the range of statistically significant associations at the *P* < .05 level and dark beige shaded areas indicate statistically significant associations after correction for multiple testing (*P* < .025).

In INMA, warmer temperatures were associated with more attention problems, with cumulative exposure between 19.7 °C and 21.3 °C (77th-84th percentile) 1 month prior to the outcome assessment, and between 15.6 °C and 24.3 °C (55th-95th percentile) 2 months prior, surviving correction for multiple testing (Figures 3F and I; eTable 9 in Supplement 1). For example, the estimated cumulative 2-month exposure of 21.7 °C (86th percentile) was associated with a 1.52 (95% CI, 0.38-2.66) higher square-root transformed attention problems score (corresponding to a raw score of 2.3). Two-week exposure to warmer temperatures was also associated with more internalizing problems, but associations did not survive correction for multiple testing (Figure 3A and eTable 9 in Supplement 1). No associations for externalizing problems or for cold were found in INMA. Stratifying by neighborhood socioeconomic status levels revealed no associations (eFigure 4 in Supplement 1).

Follow-up analysis in Generation R revealed similar curves to the main analysis for exposure to ambient temperature and adolescent-reported internalizing problems, albeit insignificant (eFigure 5 in Supplement 1). Results for maternal anxiety or depressive symptoms at an earlier child age of 9 years did not indicate significant associations (eFigure 5 in Supplement 1).

Sensitivity analyses adjusting the models for the presence of air conditioning or heating in the household in INMA showed similar associations (eFigure 6 and eFigure 7 in Supplement 1). Sensitivity analyses changing the lag period to 3 days (eFigure 8 in Supplement 1) or testing the robustness of results by changing the knot placements in the associations of exposure or lag with response revealed similar curves as those obtained by primary model choice parameters in both cohorts (eFigures 9-14 in Supplement 1).

## Discussion

This cross-national cohort study revealed that exposure to colder temperatures was associated with more internalizing problems in adolescents in the Netherlands, whereas exposure to warmer temperatures was associated with more attention problems in adolescents in Spain. These associations were mainly observed for the 2-month exposure before outcome assessment. These findings highlight how colder and warmer temperatures impact psychiatric well-being on different symptomatic levels.

Limited research on the health impacts of cold exposure have established that the body’s temperature regulation response to cold is slower than to heat, lasting weeks.^3,39^ Animal studies indicate cold exposure inhibits warmth-sensitive neurons via skin cold receptors, and in humans, it alters core body temperature, disrupting cell function, including in the brain.^7,42^ This stress triggers thermoregulatory responses like vasoconstriction, inflammation, and shivering.^3,7,43^ A meta-analysis^44^ found daily cold temperatures (1st-2.5th percentile) were not associated with worsening psychiatric disorders. However, meta-analyzed studies using DLNM had lower spatial resolution than this study (city and national level vs our residential level) and mainly evaluated hospitalization records or clinical diagnoses of disorders, complicating direct comparisons with our results. Additionally, psychiatric symptom scales are more sensitive to change, further challenging comparisons. To the best of our knowledge, no previous studies utilizing DLNM evaluated psychiatric symptoms.^44^ Our results show that subtler cold exposure (3.3 °C to 12.4 °C) in the Netherlands was associated with more internalizing problems, suggesting potential susceptibility to anxiety and depressive symptoms in colder conditions, possibly as a result of thermal discomfort. The absence of these associations in Spain may be due to insufficient cold exposure to trigger a physiological response (5th percentile: 8.0 °C in INMA vs 2.7 °C in Generation R for the 2-month period).

Studies have identified how the body responds to heat, which can include an increase in the levels of systemic inflammation markers and cortisol and disruption of proper cooling and oxygenation of the brain.^7,8,45,46^ Research suggests that exposure to heat can elevate core body temperature, triggering heat exhaustion and thermal discomfort, manifesting as changes in behavior like increased irritability.^7,10^ We identified that subtle warm temperatures (15.6 °C to 24.3 °C) were associated with more attention problems in 3 regions in Spain, suggesting that physiological responses may worsen with increased temperatures. Heat exposure, a major climate change threat, has been associated with increased risk of mental health end points, like emergency department visits or suicide.^6,16,44,47^ Individuals with preexisting mental disorders are particularly vulnerable to hospitalization when exposed to heat.^14^ To the best of our knowledge, no study has evaluated the association of ambient temperature with attention problems in adolescents. Scientific evidence repeatedly warns of heat-related mental health risks,^5,6^ and our results further underscore the importance of improved public health strategies. Disrupted sleep could mediate this association, with increased night temperatures having been associated with sleep disturbances,^7,8,48^ and disrupted sleep with poorer performance on attention tasks.^6,49^ The null associations in Generation R could be because a more prolonged high temperature exposure is needed to trigger attention problems.

We found no associations of temperature with externalizing problems. The heat-aggression hypothesis by Anderson and colleagues^50^ stipulates that hotter temperatures are associated with violence and aggression. However, a meta-analysis could not confirm this association.^50,51^ Only 1 study^20^ previously looked at psychiatric symptoms in children and adolescents and found that long-term exposure to high temperatures was associated with more aggressive behavior. Long-term exposure was estimated by aggregating temperature 1, 2, and 3 years before outcome assessment,^20^ hindering comparison with our findings. Finally, teachers might more accurately report externalizing problems, highlighting potential discrepancies in our parent-reported levels.

This study encompasses 3 main strengths. First, we used residential-level temperature data validated against European monitoring stations, with high temporal resolution, and considered address changes. Second, the DLNM approach evaluates the delayed cumulative associations of temperature exposure, providing flexibility to model both cold and heat, and considers time-series correlations. Third, we studied a continuum of psychiatric symptoms associated with anxiety and depression, aggression and violence, and attention deficit/hyperactivity disorder, rather than evaluating clinical end points, enhancing the study’s power.

### Limitations

The results should be interpreted in the context of several limitations. Primarily, exposure misclassification cannot be disregarded. First, we lacked information on participants’ behavioral patterns away from home. Second, ambient temperature does not necessarily reflect indoor temperatures, overlooking housing conditions like insulation, air conditioning, or heating that could alter exposure levels and physiological response. In the INMA cohort, we adjusted for air conditioning and heating and observed similar results. Moreover, we attempted to account for these conditions by adjusting for residential factors, including neighborhood, household composition, and parental socioeconomic characteristics. Additionally, evaluating temperature metrics such as cold spells and heatwaves could yield insights for future studies. Further, despite the assessment of a broader range of psychiatric symptoms bringing novelty to this study, we lacked information on the exact time frame of participants’ internalizing, externalizing, and attention problems. To capture current psychiatric symptoms, we examined 2-week, 1-month, and 2-month exposure periods, plus a 3-day period for sensitivity analysis, but some critical exposure windows may remain unexamined. Another limitation of the CBCL/6-18 questionnaire is possible maternal reporting bias, in which the current emotional state of the mother might influence their perception of the child’s. Yet, sensitivity analyses revealed no association of temperature with maternal anxiety or depressive symptoms, and the curves of the associations of temperature exposure with adolescent-reported internalizing symptoms showed a similar shape, albeit not significant. Additionally, DLNM lacks conventional guidelines for selecting the most appropriate model for the association of exposure and lag with response.^3^ We ensured robustness of findings by conducting sensitivity analyses and changing model parameterization.

## Conclusions

In conclusion, we highlight distinct associations of temperature exposure with adolescent psychiatric symptoms in 2 European countries; cold was associated with more internalizing problems in the Netherlands, and heat was associated with more attention problems in Spain. As climate change intensifies, these associations may become more pronounced in the near future. Studies should include individual-level exposure and evaluate psychiatric symptoms in adolescents from countries with varying temperatures. This study helps quantify the intricate and multifactorial nature of the association of climate change with mental health and can be leveraged to provide evidence for adaption strategies and climate action policies.

## Notes

Supplement 1. **eFigure 1.** Flowchart of the Study Population in the Two European Birth Cohorts **eAppendix 1.** Detailed Description of the Temperature Exposure Assessment **eTable 1.** Detailed Descriptives of Psychiatric Symptom Scores **eFigure 2.** DAG of the Temperature and Internalizing, Externalizing, and Attention Problems Associations **eTable 2.** Time Points of Data Collection for Potential Confounding Variables **eTable 3.** Details of the Expectation-Maximization Imputation **eTable 4.** Population Characteristics in Observed and Expectation-Maximization Imputed Datasets in the Two European Birth Cohorts **eTable 5.** Population Characteristics of Participants Included and Excluded in the Analysis in the Two European Birth Cohorts **eTable 6.** List of Variables Used as Predictors of Participation in the Covariate Balancing Propensity Score Procedure for Inverse Probability Weighting in the Two European Birth Cohorts **eFigure 3.** Distribution of Inverse Probability Weights for the Analysis Sample of Each Study Population in the Two European Birth Cohorts **eAppendix 2.** Details of the DLNM and Its Parameterization Decisions **eTable 7.** Optimum Temperatures Associated With the Lowest Outcome Score in the Two European Birth Cohorts **eTable 8.** Cumulative Associations Between Ambient Temperature Exposure Between Percentiles 5 to 95 Two Weeks, One Month, and Two Months Prior to CBCL Assessment and Internalizing, Externalizing, or Attention Problems in Generation R **eFigure 4.** Cumulative Associations Between Temperature Exposure During the Two Months Prior to Outcome Assessment and Internalizing Problems in Generation R or Attention Problems in INMA, Split by Low Versus High Neighbourhood Socioeconomic Status Level **eTable 9.** Cumulative Associations Between Ambient Temperature Exposure Between Percentiles 5 to 95 Two Weeks, One Month, and Two Months Prior to CBCL Assessment and Internalizing, Externalizing, or Attention Problems in INMA **eFigure 5.** Cumulative Associations Between Temperature Exposure During the Two Weeks or Two Months Prior to Outcome Assessment and Self-Reported Internalizing Problems From the YSR Assessment at Child Fourteen Years and Maternal Anxiety/Depressive Symptoms From the BSI Assessment at Child Nine Years in Generation R **eFigure 6.** Cumulative Associations Between Daily Temperature Exposure Two Weeks and Two Months Prior to CBCL Assessment and Internalizing, Externalizing, and Attention Problem in Adolescents From INMA, Additionally Adjusted for the Presence of Air Conditioning in the Household **eFigure 7.** Cumulative Associations Between Daily Temperature Exposure Two Weeks and Two Months Prior to CBCL Assessment and Internalizing, Externalizing, and Attention Problem in Adolescents From INMA, Additionally Adjusted for the Presence of Heating in the Household **eFigure 8.** Cumulative Associations Between Temperature Exposure Three Days Prior to the Outcome Assessment and Internalizing, Externalizing, and Attention Problems in Adolescents From Generation R and INMA **eFigure 9.** Cumulative Associations Between Temperature Exposure During the Two Weeks Prior to the Outcome Assessment and Internalizing, Externalizing, and Attention Problems in Adolescents From Generation R, With Knots Placed at the 10th and 90th Percentiles of Temperature Distribution in the Exposure-Response Relationship or the Lag-Response Relationship Modeled With: 1 Knot in the Log-Scale of the Lag Period, 1 Equally Distributed Knot in the Lag Period, or 2 Equally Distributed Knots in the Lag Period **eFigure 10.** Cumulative Associations Between Temperature Exposure During the One Month Prior to the Outcome Assessment and Internalizing, Externalizing, and Attention Problems in Adolescents From Generation R, With Knots Placed at the 10th and 90th Percentiles of Temperature Distribution in the Exposure-Response Relationship or the Lag-Response Relationship Modeled With: 1 Knot in the Log-Scale of the Lag Period, 1 Equally Distributed Knot in the Lag Period, or 2 Equally Distributed Knots in the Lag Period **eFigure 11.** Cumulative Associations Between Temperature Exposure During the Two Months Prior to the Outcome Assessment and Internalizing, Externalizing, and Attention Problems in Adolescents From Generation R, With Knots Placed at the 10th and 90th Percentiles of Temperature Distribution in the Exposure-Response Relationship or the Lag-Response Relationship Modeled With: 1 Knot in the Log-Scale of the Lag Period, 1 Equally Distributed Knot in the Lag Period, or 2 Equally Distributed Knots in the Lag Period **eFigure 12.** Cumulative Associations Between Temperature Exposure During the Two Weeks Prior to the Outcome Assessment and Internalizing, Externalizing, and Attention Problems in Adolescents From INMA, With Knots Placed at the 10th and 90th Percentiles of Temperature Distribution in the Exposure-Response Relationship or the Lag-Response Relationship Modeled With: 1 Knot in the Log-Scale of the Lag Period, 1 Equally Distributed Knot in the Lag Period, or 2 Equally Distributed Knots in the Lag Period **eFigure 13.** Cumulative Associations Between Temperature Exposure During the One Month Prior to the Outcome Assessment and Internalizing, Externalizing, and Attention Problems in Adolescents From INMA, With Knots Placed at the 10th and 90th Percentiles of Temperature Distribution in the Exposure-Response Relationship or the Lag-Response Relationship Modeled With: 1 Knot in the Log-Scale of the Lag Period, 1 Equally Distributed Knot in the Lag Period, or 2 Equally Distributed Knots in the Lag Period **eFigure 14.** Cumulative Associations Between Temperature Exposure During the Two Months Prior to the Outcome Assessment and Internalizing, Externalizing, and Attention Problems in Adolescents From INMA, With Knots Placed at the 10th and 90th Percentiles of Temperature Distribution in the Exposure-Response Relationship or the Lag-Response Relationship Modeled With: 1 Knot in the Log-Scale of the Lag Period, 1 Equally Distributed Knot in the Lag Period, or 2 Equally Distributed Knots in the Lag Period
eReferences

Supplement 2.
Data Sharing Statement
