# Supplementary material for: Temperature Exposure and Psychiatric Symptoms in Adolescents From 2 European Birth Cohorts
Source: JAMA Netw Open. 2025 Jan 28;8(1):e2456898. doi: 10.1001/jamanetworkopen.2024.56898 (PMC11775747; doi:10.1001/jamanetworkopen.2024.56898)
Supplement: Supplement 1. — eFigure 1. Flowchart of the Study Population in the Two European Birth Cohorts eAppendix 1. Detailed Description of the Temperature Exposure Assessment eTable 1. Detailed Descriptives of Psychiatric Symptom Scores eFigure 2. DAG of the Temperature and Internalizing, Externalizing, and Attention Problems Associations eTable 2. Time Points of Data Collection for Potential Confounding Variables eTable 3. Details of the Expectation-Maximization Imputation eTable 4. Population Characteristics in Observed and Expectation-Maximization Imputed Datasets in the Two European Birth Cohorts eTable 5. Population Characteristics of Participants Included and Excluded in the Analysis in the Two European Birth Cohorts eTable 6. List of Variables Used as Predictors of Participation in the Covariate Balancing Propensity Score Procedure for Inverse Probability Weighting in the Two European Birth Cohorts eFigure 3. Distribution of Inverse Probability Weights for the Analysis Sample of Each Study Population in the Two European Birth Cohorts eAppendix 2. Details of the DLNM and Its Parameterization Decisions eTable 7. Optimum Temperatures Associated With the Lowest Outcome Score in the Two European Birth Cohorts eTable 8. Cumulative Associations Between Ambient Temperature Exposure Between Percentiles 5 to 95 Two Weeks, One Month, and Two Months Prior to CBCL Assessment and Internalizing, Externalizing, or Attention Problems in Generation R eFigure 4. Cumulative Associations Between Temperature Exposure During the Two Months Prior to Outcome Assessment and Internalizing Problems in Generation R or Attention Problems in INMA, Split by Low Versus High Neighbourhood Socioeconomic Status Level eTable 9. Cumulative Associations Between Ambient Temperature Exposure Between Percentiles 5 to 95 Two Weeks, One Month, and Two Months Prior to CBCL Assessment and Internalizing, Externalizing, or Attention Problems in INMA eFigure 5. Cumulative Associations Between Temperature Exposure During the Two W [file jamanetwopen-e2456898-s001.pdf]

Supplemental Online Content

Essers E, Kusters M, Granés L, et al. Temperature exposure and psychiatric symptoms in adolescents from 2 European birth cohorts. *JAMA Netw. Open*. 2025;8(1):e2456898. doi:10.1001/jamanetworkopen.2024.56898

eFigure 1. Flowchart of the Study Population in the Two European Birth Cohorts

eAppendix 1. Detailed Description of the Temperature Exposure Assessment

eTable 1. Detailed Descriptives of Psychiatric Symptom Scores

eFigure 2. DAG of the Temperature and Internalizing, Externalizing, and Attention Problems Associations

eTable 2. Time Points of Data Collection for Potential Confounding Variables

eTable 3. Details of the Expectation-Maximization Imputation

eTable 4. Population Characteristics in Observed and Expectation-Maximization Imputed Datasets in the Two European Birth Cohorts

eTable 5. Population Characteristics of Participants Included and Excluded in the Analysis in the Two European Birth Cohorts

eTable 6. List of Variables Used as Predictors of Participation in the Covariate Balancing Propensity Score Procedure for Inverse Probability Weighting in the Two European Birth Cohorts

eFigure 3. Distribution of Inverse Probability Weights for the Analysis Sample of Each Study Population in the Two European Birth Cohorts

eAppendix 2. Details of the DLNM and Its Parameterization Decisions

eTable 7. Optimum Temperatures Associated With the Lowest Outcome Score in the Two European Birth Cohorts

eTable 8. Cumulative Associations Between Ambient Temperature Exposure Between Percentiles 5 to 95 Two Weeks, One Month, and Two Months Prior to CBCL Assessment and Internalizing, Externalizing, or Attention Problems in Generation R

eFigure 4. Cumulative Associations Between Temperature Exposure During the Two Months Prior to Outcome Assessment and Internalizing Problems in Generation R or Attention Problems in INMA, Split by Low Versus High Neighbourhood Socioeconomic Status Level

© 2025 Essers E et al. *JAMA Network Open*.

eTable 9. Cumulative Associations Between Ambient Temperature Exposure Between Percentiles 5 to 95 Two Weeks, One Month, and Two Months Prior to CBCL Assessment and Internalizing, Externalizing, or Attention Problems in INMA

eFigure 5. Cumulative Associations Between Temperature Exposure During the Two Weeks or Two Months Prior to Outcome Assessment and Self-Reported Internalizing Problems From the YSR Assessment at Child Fourteen Years and Maternal Anxiety/Depressive Symptoms From the BSI Assessment at Child Nine Years in Generation R

eFigure 6. Cumulative Associations Between Daily Temperature Exposure Two Weeks and Two Months Prior to CBCL Assessment and Internalizing, Externalizing, and Attention Problem in Adolescents From INMA, Additionally Adjusted for the Presence of Air Conditioning in the Household

eFigure 7. Cumulative Associations Between Daily Temperature Exposure Two Weeks and Two Months Prior to CBCL Assessment and Internalizing, Externalizing, and Attention Problem in Adolescents From INMA, Additionally Adjusted for the Presence of Heating in the Household

eFigure 8. Cumulative Associations Between Temperature Exposure Three Days Prior to the Outcome Assessment and Internalizing, Externalizing, and Attention Problems in Adolescents From Generation R and INMA

eFigure 9. Cumulative Associations Between Temperature Exposure During the Two Weeks Prior to the Outcome Assessment and Internalizing, Externalizing, and Attention Problems in Adolescents From Generation R, With Knots Placed at the 10th and 90th Percentiles of Temperature Distribution in the Exposure-Response Relationship or the Lag-Response Relationship Modelled With: 1 Knot in the Log-Scale of the Lag Period, 1 Equally Distributed Knot in the Lag Period, or 2 Equally Distributed Knots in the Lag Period

eFigure 10. Cumulative Associations Between Temperature Exposure During the One Month Prior to the Outcome Assessment and Internalizing, Externalizing, and Attention Problems in Adolescents From Generation R, With Knots Placed at the 10th and 90th Percentiles of Temperature Distribution in the Exposure-Response Relationship or the Lag-Response Relationship Modeled With: 1 Knot in the Log-Scale of the Lag Period, 1 Equally Distributed Knot in the Lag Period, or 2 Equally Distributed Knots in the Lag Period

eFigure 11. Cumulative Associations Between Temperature Exposure During the Two Months Prior to the Outcome Assessment and Internalizing, Externalizing, and Attention Problems in Adolescents From Generation R, With Knots Placed at the 10th and 90th Percentiles of Temperature Distribution in the Exposure-Response Relationship or the Lag-Response Relationship Modeled With: 1 Knot in the Log-Scale of the Lag Period, 1 Equally Distributed Knot in the Lag Period, or 2 Equally Distributed Knots in the Lag Period

© 2025 Essers E et al. *JAMA Network Open*.

eFigure 12. Cumulative Associations Between Temperature Exposure During the Two Weeks Prior to the Outcome Assessment and Internalizing, Externalizing, and Attention Problems in Adolescents From INMA, With Knots Placed at the 10th and 90th Percentiles of Temperature Distribution in the Exposure-Response Relationship or the Lag-Response Relationship Modeled With: 1 Knot in the Log-Scale of the Lag Period, 1 Equally Distributed Knot in the Lag Period, or 2 Equally Distributed Knots in the Lag Period

eFigure 13. Cumulative Associations Between Temperature Exposure During the One Month Prior to the Outcome Assessment and Internalizing, Externalizing, and Attention Problems in Adolescents From INMA, With Knots Placed at the 10th and 90th Percentiles of Temperature Distribution in the Exposure-Response Relationship or the Lag-Response Relationship Modeled With: 1 Knot in the Log-Scale of the Lag Period, 1 Equally Distributed Knot in the Lag Period, or 2 Equally Distributed Knots in the Lag Period

eFigure 14. Cumulative Associations Between Temperature Exposure During the Two Months Prior to the Outcome Assessment and Internalizing, Externalizing, and Attention Problems in Adolescents From INMA, With Knots Placed at the 10th and 90th Percentiles of Temperature Distribution in the Exposure-Response Relationship or the Lag-Response Relationship Modeled With: 1 Knot in the Log-Scale of the Lag Period, 1 Equally Distributed Knot in the Lag Period, or 2 Equally Distributed Knots in the Lag Period

eReferences

This supplemental material has been provided by the authors to give readers additional information about their work.

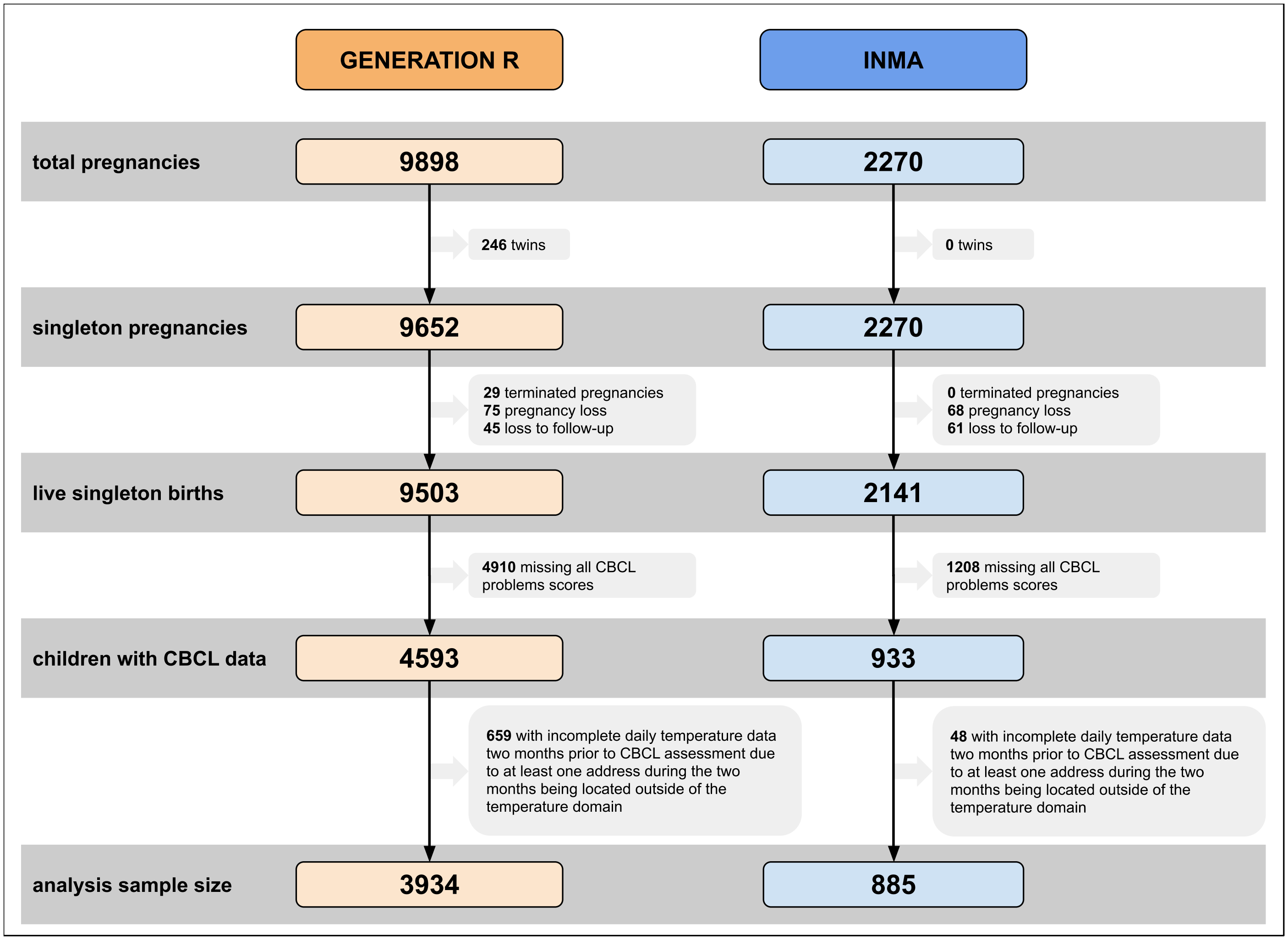

eFigure 1. Flowchart of the study population in the two European birth cohorts.

Abbreviations: CBCL, Child Behavioural Checklist

© 2025 Essers E et al. *JAMA Network Open*.

eAppendix 1. Detailed description of the temperature exposure assessment.

Accurate assessment of air temperature was ensured via the UrbClim™ model.[1] This model uses urban physics coupled with a 3D atmospheric boundary layer module that includes information on the urban structure (e.g., vegetation, land use and cover) to generate climate data at a high temporal (hourly) and spatial (100 x 100 metres) resolution.[1,2] UrbClim™ has been successfully validated in various European countries, including Spain and the UK[3–5], and the data has been used in previous projects utilizing Generation R and INMA data.[6,7] Detailed methodology is described elsewhere.[1,3–5]

The model is developed for domains of 30 x 30 km. In this study, the location of two domains for Generation R (Rotterdam and Den Haag) and a singular domain for each INMA region was placed based on the geographical area where the maximum number of participants lived based on all available geocoded addresses starting from pregnancy (87.5% for Generation R, 98.2% for INMA-Gipuzkoa, 95.3% for INMA-Sabadell, and 90.5% for INMA-Valencia). Temperature was calculated for the time period of interest (i.e., the years encompassing the two months prior to the Child Behavioural Checklist assessment): 2015 – 2019 for Generation R, 2021 – 2022 for INMA-Gipuzkoa, 2020 – 2022 for -Sabadell, and 2019 – 2021 for -Valencia. The hourly temperature data was converted into daily data based on the Coordinated Universal Time and estimated at the geocoded address where the participant lived at the time of Child Behavioural Checklist assessment, accounting for changes in address. Annex A on shows the 5th and 95th percentile of daily temperature distribution for the years of interest in the temperature domains for Generation R and the INMA regions (Gipuzkoa, Sabadell, and Valencia).

The model domains were set up using the European Terrestrial Reference System 1989 (EPSG 3035) as the coordinate system.[8] To obtain the most realistic representation for historical climate for the regions, land cover maps (identifying the spatial distribution of land cover types) were used. For Generation R, the 2006 European CORINE land cover map was used[9] and for INMA, the 2005 Sistema de Información de Ocupación del Suelo en España (SOISE) land cover map was used.[10] Annex B shows the conversion of the SOISE and CORINE classifications into the 15 classes of land cover in the UrbClim™ model. The model also uses Copernicus Land Imperviousness datasets (soil sealing maps), the Normalized Difference Vegetation Index from the TERRA satellite platforms MODIS instrument (vegetation cover maps), and data provided by the European Centre for Medium-range Weather Forecasting ERA5 reanalysis (background meteorology) as input data.[9,11,12]

© 2025 Essers E et al. *JAMA Network Open*.

Annex A. Daily temperature (ºC) at percentiles 5/95 in the temperature domains for Generation R (2015-2019) and INMA-Gipuzkoa (2021-2022), -Sabadell (2020-2022), and -Valencia (2019-2021).

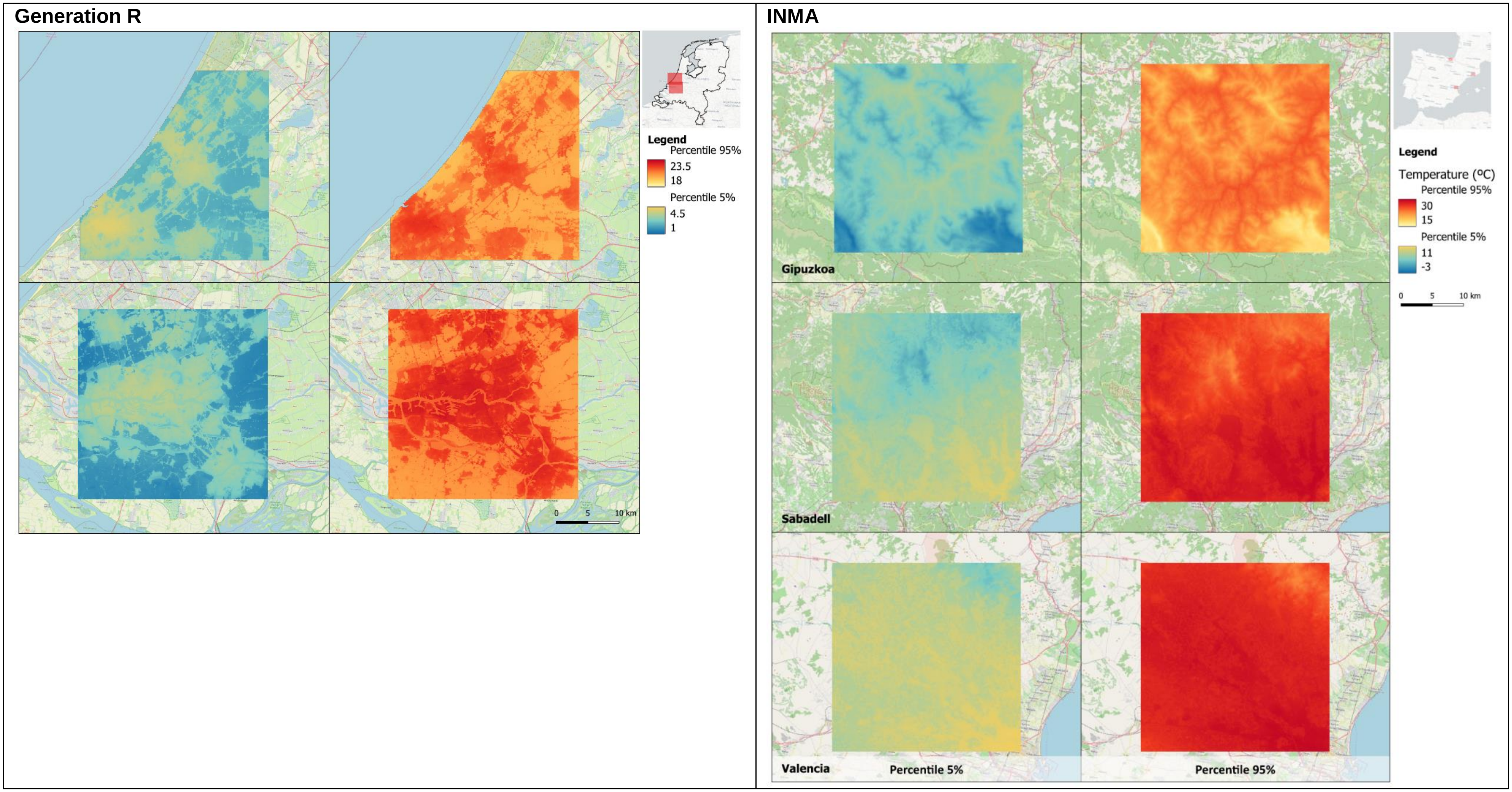

© 2025 Essers E et al. *JAMA Network Open*.

Annex B. Land use conversion tables from SOISE (using the CODIIGE codes) and CORINE classes into the fifteen UrbClim™ classes.

CODIIGE code	English name	UrbClim code	UrbClim class	CODIIGE code	English name	UrbClim code	UrbClim class
111	Helmet	1	Urban	1	Continuous urban fabric	1	Urban
112	Expansion	1	Urban	2	Discont. urban fabric	2	Suburban
113	Discontinuous	2	Suburban	3	Industrial or commercial units	3	Industrial
114	Urban green zone	4	Urban green	4	Road/rail networks and ass. land	3	Industrial
121	Agricultural and/or livestock facility	3	Industrial	5	Port areas	3	Industrial
122	Forestry facility	3	Industrial	6	Airports	3	Industrial
123	Mining extraction	3	Industrial	7	Mineral extraction sites	3	Industrial
130	Industrial	3	Industrial	8	Dump sites	3	Industrial
140	Endowment service	2	Suburban	9	Construction sites	3	Industrial
150	Agricultural settlement and orchard	2	Suburban	10	Green urban areas	4	Urban green
161	Road or rail network	3	Industrial	11	Sport and leisure facilities	4	Urban green
162	port	3	Industrial	12	Non-irrigated arable land	8	Cropland
163	Airport	3	Industrial	13	Permanent irrigated land	8	Cropland
171	Supply infrastructure	3	Industrial	14	Rice fields	8	Cropland
172	Waste infrastructure	3	Industrial	15	Vineyards	9	Shrubland
210	Herbaceous crop	8	Cropland	16	Fruit trees and berry plantations	10	Woodland
220	Greenhouse	3	Industrial	17	Olive groves	10	Woodland
231	Citrus fruit	11	Broadleaf trees	18	Pastures	7	Grassland
232	Non-citrus fruit	11	Broadleaf trees	19	Annual crops w/ permanent crops	8	Cropland
233	Vineyard	9	Shrubland	20	Complex cultivation patterns	8	Cropland
234	Olive grove	11	Broadleaf trees	21	Heterogeneous agricultural areas	8	Cropland
235	Other woody crops	10	Woodland	22	Agro-forestry areas	10	Woodland
236	Woody crop mix	9	Shrubland	23	Broad-leaved forest	11	Broadleaf trees
240	Meadow	7	Grassland	24	Coniferous forest	12	Needleleaf trees
250	Crop combination	8	Cropland	25	Mixed forest	11	Broadleaf trees
260	Combination of crops w. vegetation	8	Cropland	26	Natural grasslands	7	Grassland
311	Hardwood forest	10	Woodland	27	Moors and heathland	10	Woodland
313	Mixed forest	10	Woodland	28	Sclerophyllous vegetation	9	Shrubland
312	Coniferous forest	12	Needleleaf trees	29	Transit. woodland-shrub	9	Shrubland
320	Pasture or grassland	7	Grassland	30	Beaches, dunes, sand	6	Bare soil
330	Scrub	9	Shrubland	31	Bare rocks	6	Bare soil
340	Vegetation combination	9	Shrubland	32	Sparsely vegetated areas	9	Shrubland
351	Beach, dune or sand	6	Bare soil	33	Burnt areas	6	Bare soil
352	Roquedo	6	Bare soil	34	Glaciers & perpetual snow	5	Snow/ice
353	Temporarily deforested by fires	6	Bare soil	35	Inland marshes	9	Shrubland
354	Bare ground	6	Bare soil	36	Peat bogs	9	Shrubland
411	Wet and swampy area	7	Grassland	37	Salt marshes	7	Grassland
412	Peatbog	7	Grassland	38	Salines	7	Grassland
413	Marsh	7	Grassland	39	Intertidal flats	6	Bare soil
414	Saline	7	Grassland	40	Water courses	13	River
514	Artificial water foil	14	Inland water body	41	Water bodies	14	Inland water body
511	Water course	13	River	42	Coastal lagoons	15	Sea
512	Lake or lagoon	14	Inland water body	43	Estuaries	15	Sea
513	Reservoir	14	Inland water body	44	Sea and ocean	15	Sea
515	Sea	15	Sea				
516	Glacier and / or perpetual snow	5	Snow/Ice				

© 2025 Essers E et al. *JAMA Network Open*.

eTable 1. Detailed descriptives of psychiatric symptom scores

		Generation R								
		observed range							***theoretical range***	
		Mean	*Standard Deviation*	*Minimum*	*Percentile 25*	*Median*	*Percentile 75*	*Maximum*	*Minimum*	*Maximum*
Raw scores	Internalizing Problems	5.7	5.8	0.0	1.0	4.0	8.0	41.0	0.0	64.0
	Externalizing Problems	4.3	5.3	0.0	0.0	2.0	6.0	48.0	0.0	70.0
	Attention Problems	3.4	3.2	0.0	1.0	3.0	5.0	18.0	0.0	20.0
Square-root transformed scores	Internalizing Problems	2.0	1.2	0.0	1.0	2.0	2.8	6.4	0.0	8.0
	Externalizing Problems	1.6	1.3	0.0	0.0	1.4	2.5	6.9	0.0	8.4
	Attention Problems	1.5	1.0	0.0	1.0	1.7	2.2	4.2	0.0	4.5
		**INMA**								
		observed range							***theoretical range***	
		Mean	*Standard Deviation*	*Minimum*	*Percentile 25*	*Median*	*Percentile 75*	*Maximum*	*Minimum*	*Minimum*
Raw scores	Internalizing Problems	7.4	6.5	0.0	3.0	6.0	11.0	42.0	0.0	64.0
	Externalizing Problems	6.2	6.5	0.0	1.0	4.0	9.0	57.0	0.0	70.0
	Attention Problems	3.5	3.6	0.0	1.0	2.0	6.0	16.0	0.0	20.0
Square-root transformed scores	Internalizing Problems	2.4	1.2	0.0	1.7	2.5	3.3	6.5	0.0	8.0
	Externalizing Problems	2.1	1.3	0.0	1.0	2.0	3.0	7.6	0.0	8.4
	Attention Problems	1.5	1.1	0.0	1.0	1.4	2.5	4.0	0.0	4.5

© 2025 Essers E et al. *JAMA Network Open*.

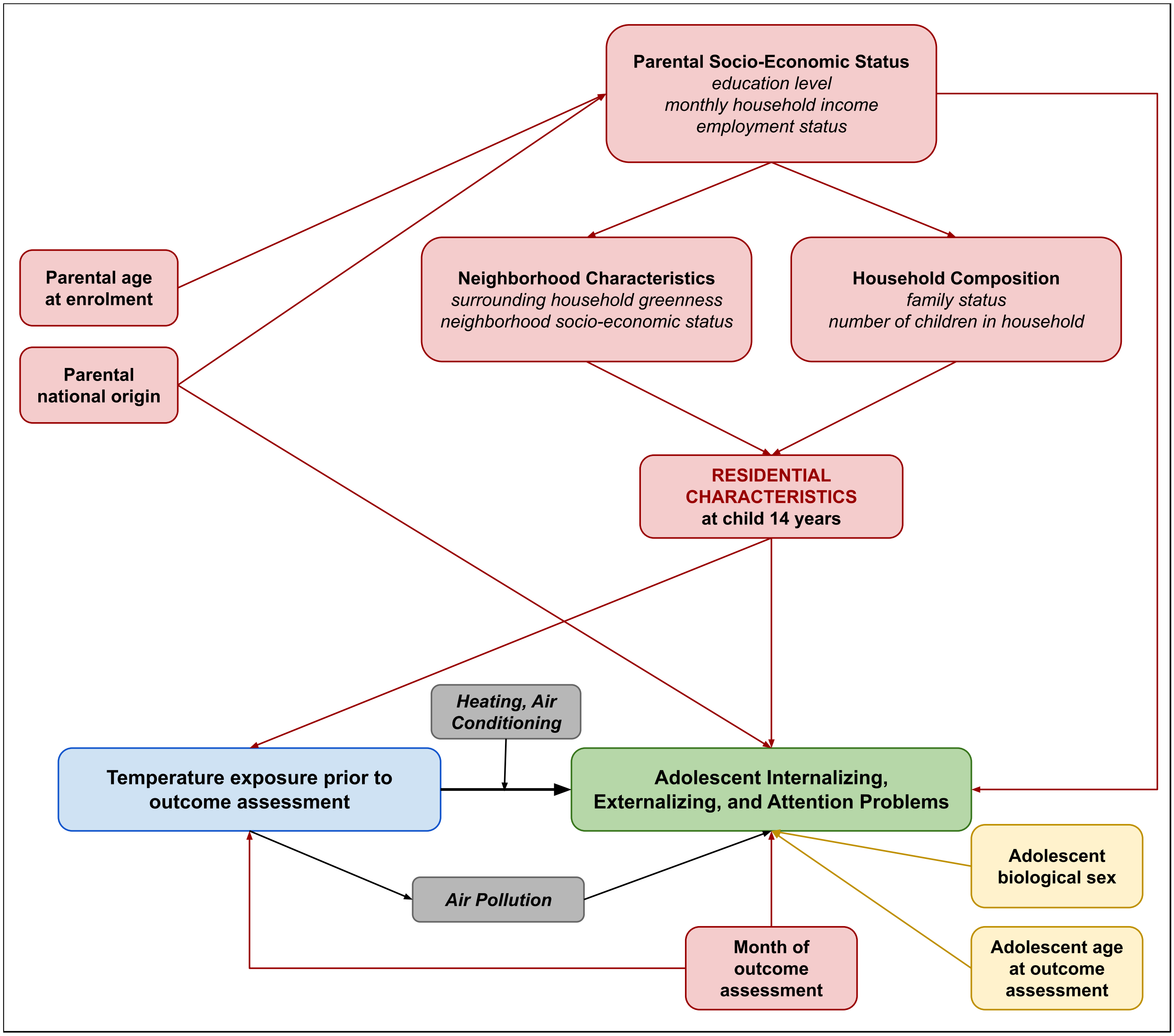

eFigure 2. Directed acyclic graph (DAG) of the temperature and internalizing, externalizing, and attention problems association.

The node in blue represents the exposure and in green the outcome. Red nodes and arrows represent potential confounding variables and pathways, yellow nodes represent ancestors of the outcome. Air pollution can be seen as a potential mediator with its biasing pathway in grey, and heating or air conditioning as a potential moderator.

© 2025 Essers E et al. *JAMA Network Open*.

eTable 2. Time points of data collection for potential confounding variables.

Potential Confounding Variable	Generation R			INMA		
	Baseline	*Follow-up at 5 years*	*Follow-up at 13 years* [a]	*Baseline*	*Follow-up at 11 years*	*Follow-up at 14-16 years* [a]
Adolescent biological sex	X			X		
Maternal age	X			X		
Paternal age	X			X		
Maternal national origin	X			X		
Paternal national origin	X			X		
Adolescent age			X [b]			X [b]
Maternal education level		X			X	X
Paternal education level		X			X	X
Number of children in the household		X				X
Monthly household income			X			X
Family status			X		X	X
Maternal employment status			X		X	X
Paternal employment status			X		X	X
Residential greenness exposure			X [c]			X [c]
Neighbourhood socioeconomic status			X [c]			X [c]
Month of outcome assessment			X [b]			X [b]

[a] outcome assessment was done during these follow-ups
[b] calculated for the exact date of the outcome assessment
[c] calculated at the residential address for the year of the outcome assessment

© 2025 Essers E et al. *JAMA Network Open*.

eTable 3. Details of the expectation-maximization imputation.

EXPECTATION-MAXIMIZATION IMPUTATION
**Software used and key setting:** R (version 4.3.0; R Core Team (2023)) – Amelia package v1.8.1
**Number of imputed datasets created:** 1
**Sample size for imputation:**<br>3934 for Generation R, 280 for INMA-Gipuzkoa, 352 for INMA-Sabadell, and 253 for INMA-Valencia
**Variables with repeated measures averaged into one value for imputation:**<br>Daily ambient temperature from 1 day until 56 days prior to the outcome assessment
**Variables used as predictors for the imputation:**<br>Adolescent biological sex<br>Maternal age at enrolment<br>Paternal age at enrolment<br>Maternal pre-pregnancy body mass index<br>Paternal body mass index at enrolment<br>Maternal national origin [a]<br>Paternal national origin [a]<br>Intelligence quotient mother [b]<br>Maternal educational level [b]<br>Paternal educational level [b]<br>Number of children in the household [b]<br>Residential surrounding greenness [b]<br>Residential neighbourhood socio-economic status [b]<br>Monthly household income [b]<br>Maternal smoking habit [b]<br>Maternal alcohol use [b, c]<br>Family status [b]<br>Maternal employment status [b]<br>Paternal employment status [b]<br>Averaged ambient temperature two months prior to outcome assessment<br>Internalizing problems score<br>Externalizing problems score<br>Attention problems score
**Diagnostics:** Visual inspection by checking convergence with convergence plots.
**References:** [13]

[a] Not used in the INMA-Gipuzkoa cohort due to too low variability between the categories
[b] Variables collected at the time of or as close as possible to the outcome assessment
[c] Unavailable in the INMA-Valencia cohort

eTable 4. Population characteristics in observed and expectation-maximization imputed datasets in the two European birth cohorts.

	Generation R (*N = 3,934*)			INMA								
				Gipuzkoa (*N = 280*)			Sabadell (*N = 352*)			Valencia (*N = 253*)		
	obs.	imp.	% imp.	obs.	imp.	% imp.	obs.	imp.	% imp.	obs.	imp.	% imp.
Adolescent Characteristics[1]												
Internalizing problems	2.0 (1.0; 2.8)	2.0 (1.0; 2.8)	0.03	2.0 (1.4; 2.7)	-	0.0	2.5 (1.7; 3.3)	-	0.0	2.7 (2.0; 3.6)	-	0.0
Externalizing problems	1.4 (0.0; 2.5)	1.4 (0.0; 2.5)	0.3	1.7 (1.0; 2.8)	-	0.0	2.0 (1.0; 3.0)	-	0.0	2.2 (1.4; 3.3)	-	0.0
Attention problems	1.7 (1.0; 2.2)	1.7 (1.0; 2.2)	0.3	1.4 (0.0; 2.2)	-	0.0	1.7 (1.0; 2.5)	-	0.0	1.7 (1.0; 2.7)	-	0.0
Maternal Characteristics												
Age (years)	31.4 (4.9)	-	0.0	31.7 (3.3)	-	0.0	30.9 (3.9)	30.9 (3.9)	1.1	31.0 (4.0)	-	0.0
Body mass index (kg/m^2)	23.4 (4.1)	23.5 (4.0)	23.9	23.1 (3.6)	-	0.0	23.9 (4.5)	23.9 (4.6)	2.3	23.5 (4.1)	-	0.0
Education level			10.9			1.8			3.4			0.4
High	60.5	57.8		53.8	53.9		36.2	36.6		34.5	34.4	
Medium	29.8	31.0		37.1	37.1		38.8	37.5		41.7	41.9	
Low	9.6	11.1		9.1	8.9		25.0	25.9		23.8	23.7	
National origin			1.1			0.0			1.4			0.0
Country of cohort	60.6	60.2		98.6	-		90.8	90.9		96.4	-	
Suriname	7.0	7.1		na	-		na	na		na	-	
Turkey	5.2	5.3		na	-		na	na		na	-	
Morocco	4.3	4.4		na	-		na	na		na	-	
Other, European	7.9	8.0		na	-		na	na		na	-	
Other, non-European	15.1	14.9		na	-		na	na		na	-	
Other	na	na		1.4	-		9.2	9.1		36	-	
Alcohol use (no *vs.* yes)	27.8	27.7	0.8	39.6	39.3	1.8	53.5	54.5	3.4	na	na	
Smoking habit (no *vs.* yes)	88.3	88.0	1.6	82.9	83.2	1.8	77.3	77.0	3.7	72.9	72.3	9.5
Intelligence quotient	98.1 (14.5)	97.6 (14.5)	10.4	100.9 (13.4)	100.4 (13.6)	18.2	104.5 (14.4)	104.5 (14.3)	2.6	101.7 (17.4)	101.5 (17.3)	2.4
Employment status (paid *vs.* unpaid job)	81.7	80.9	4.0	90.0	-	0.0	86.1	-	0.0	80.3	80.6	1.6
Paternal Characteristics												
Age (years)	33.9 (5.6)	33.9 (5.6)	12.9	34.0 (4.5)	34.0 (4.5)	0.4	32.7 (4.5)	32.6 (4.5)	12.2	32.6 (4.8)	-	0.0
Body mass index (kg/m^2)	25.2 (3.4)	25.3 (3.4)	26.6	25.5 (3.1)	25.6 (3.1)	3.2	26.0 (3.7)	26.0 (3.8)	2.0	25.9 (3.4)	-	0.0
Education level			17.4			5.0			11.4			4.0
High	60.5	56.9		29.3	29.3		27.6	28.1		23.5	22.9	
Medium	26.3	26.9		48.9	49.3		39.7	40.6		36.2	35.2	
Low	13.2	16.3		21.8	21.4		32.7	31.2		40.3	41.9	
National origin			4.3			0.0			0.6			0.0
Country of cohort	63.6	62.3		99.3	-		90.3	90.3		91.3	-	
Suriname	6.5	6.7		na	-		na	na		na	-	
Turkey	5.3	5.7		na	-		na	na		na	-	
Morocco	4.6	4.8		na	-		na	na		na	-	
Other, European	5.2	5.5		na	-		na	na		na	-	
Other, non-European	15.0	15.0		na	-		na	na		na	-	
Other	na	na		0.7	-		9.7	9.7		8.7	-	
Employment status (paid *vs.* unpaid job)	90.7	88.9	8.5	97.0	97.1	4.3	92.3	90.9	11.9	90.6	87.8	11.5

Values are percentage for categorical and mean (standard deviation) for continuous variables. Abbreviations: imp, imputed; na, not available; obs, observed.

[1] Values are the square-root transformed problem scores.

© 2025 Essers E et al. *JAMA Network Open*.

eTable 4, continued. Population characteristics in observed and expectation-maximization imputed datasets in the two European birth cohorts.

	Generation R *(N = 3,934)*			INMA								
				Gipuzkoa *(N = 280)*			Sabadell *(N = 352)*			Valencia *(N = 253)*		
	obs.	imp.	% imp.	obs.	imp.	% imp.	obs.	imp.	% imp.	obs.	imp.	% imp.
Household Characteristics												
Residential surrounding greenness	0.3 (0.1)	0.3 (0.1)	0.8	0.5 (0.1)	-	0.0	0.3 (0.1)	-	0.0	0.2 (0.1)	-	0.0
Family status (dual *vs.* single parent)	80.1	80.1	0.9	93.6	-	0.0	84.5	84.1	2.8	86.8	86.6	0.8
Monthly household income			10.2			14.3			21.6			3.2
low	17.6	18.3		2.5	3.2		14.9	15.3		20.8	20.2	
low-medium	27.3	26.3		11.2	12.5		20.3	23.9		27.4	26.9	
medium-high	36.5	36.8		35.8	37.1		39.5	35.5		39.2	39.5	
high	18.6	18.6		50.4	47.1		25.4	25.3		12.7	13.4	
Number of children in the household			12.9			2.5			8.0			0.4
1	18.5	19.8		8.4	8.6		16.4	17.3		23.8	23.7	
2	55.8	55.1		70.3	70.0		71.6	70.2		65.1	65.2	
3 or more	25.7	25.1		21.2	21.4		12.0	12.5		11.1	11.1	

Values are percentage for categorical and mean (standard deviation) for continuous variables. Abbreviations: imp, imputed; na, not available; obs, observed.

[1] Values are the square-root problem scores.

© 2025 Essers E et al. *JAMA Network Open*.

eTable 5. Population characteristics of participants included and excluded in the analysis in the two European birth cohorts

	Generation R			INMA								
				Gipuzkoa			Sabadell			Valencia		
	Included *(N = 3,934)*	Not Included *(N = 5,738)*	P-value	Included *(N = 280)*	Not Included *(N = 358)*	P-value	Included *(N = 352)*	Not Included *(N = 425)*	P-value	Included *(N = 253)*	Not Included *(N = 602)*	P-value
Maternal Characteristics												
Age (years)	31.4 (4.9)	28.8 (5.5)	**< 0.001**	31.7 (3.3)	31.1 (3.9)	**0.039**	30.9 (3.9)	29.6 (5.0)	**< 0.001**	31.0 (4.0)	29.2 (4.8)	**< 0.001**
Body mass index (kg/m^2)	23.4 (4.1)	23.8 (4.6)	**0.004**	23.1 (3.6)	22.9 (2.7)	0.256	23.9 (4.5)	23.7 (4.4)	0.611	23.5 (4.1)	23.9 (4.9)	0.653
Education level			**< 0.001**			0.526			0.491			0.491
High	60.5	50.9		53.8	47.6		36.2	31.3		34.5	28.9	
Medium	29.8	31.8		37.1	41.0		38.8	39.5		41.7	45.9	
Low	9.6	17.4		9.1	11.4		25.0	29.2		23.8	25.2	
National origin			**< 0.001**			**0.002**			**0.002**			**< 0.001**
Country of cohort	60.6	41.7		98.6	93.3		90.8	82.7		96.4	84.2	
Suriname	7.0	10.3		na	na		na	na		na	na	
Turkey	5.2	11.6		na	na		na	na		na	na	
Morocco	4.3	8.5		na	na		na	na		na	na	
Other, European	7.9	8.1		na	na		na	na		na	na	
Other, non-European	15.1	19.8		na	na		na	na		na	na	
Other	na	na		1.4	6.7		9.2	17.3		7.6	15.8	
Alcohol use (no *vs.* yes)	27.7	15.2	**< 0.001**	39.6	41.4	0.853	53.5	57.5	0.475	na	na	na
Smoking habit (no *vs.* yes)	88.3	91.1	**0.037**	82.9	76.0	0.164	77.3	66.2	**0.015**	72.9	63.4	0.063
Intelligence quotient	98.1 (3.4)	92.6 (15.9)	**< 0.001**	100.9 (13.4)	96.2 (13.7)	0.174	104.5 (14.1)	100.7 (14.8)	**0.009**	101.7 (17.4)	96.3 (15.3)	**< 0.001**
Employment status (paid *vs.* unpaid job)	81.7	81.9	0.939	90.0	84.5	0.185	86.1	77.9	**0.036**	80.3	67.7	**0.006**
Paternal Characteristics												
Age (years)	33.9 (5.6)	32.2 (6.3)	**< 0.001**	34.0 (4.5)	33.5 (4.4)	**0.002**	32.7 (4.5)	31.8 (5.8)	**0.004**	32.6 (4.8)	31.4 (5.2)	**< 0.001**
Body mass index (kg/m^2)	25.2 (3.4)	25.4 (3.6)	0.094	25.5 (3.1)	25.6 (3.1)	0.648	26.0 (3.7)	25.7 (3.3)	0.715	25.9 (3.4)	25.9 (3.6)	0.662
Education level			**< 0.001**			0.108			0.238			0.292
High	60.5	53.1		29.3	27.3		27.6	20.8		23.5	17.4	
Medium	26.3	25.9		48.9	40.4		39.7	39.2		36.2	36.1	
Low	13.2	21.0		21.8	32.3		32.7	40.0		40.3	46.5	
National origin			**< 0.001**			**< 0.001**			**0.026**			**0.002**
Country of cohort	63.6	44.0		99.3	93.3		90.3	84.6		91.3	82.6	
Suriname	6.5	9.9		na	na		na	na		na	na	
Turkey	5.3	11.0		na	na		na	na		na	na	
Morocco	4.6	9.3		na	na		na	na		na	na	
Other, European	5.2	6.4		na	na		na	na		na	na	
Other, non-European	14.9	19.3		na	na		na	na		na	na	
Other	na	na		0.7	6.7		9.7	15.4		8.7	17.4	
Employment status (paid *vs.* unpaid job)	90.7	93.5	**0.025**	97.0	97.0	1.00	92.3	93.6	0.810	90.6	86.8	0.358
Household Characteristics												
Residential surrounding greenness	0.3 (0.1)	0.3 (0.1)	**< 0.001**	0.3 (0.1)	0.4 (0.0)	0.328	0.3 (0.1)	0.3 (0.1)	0.202	0.2 (0.1)	0.2 (0.1)	**0.042**
Family status (dual *vs.* single parent)	80.1	85.3	**0.002**	93.6	92.4	0.852	84.5	72.8	**0.004**	86.8	73.1	**0.001**
Monthly household income			**< 0.001**			0.806			0.335			0.324
low	17.6	11.1		2.5	0.0		14.9	0.0		20.8	15.8	
low-medium	27.3	16.8		11.2	0.0		20.3	33.3		27.4	42.1	
medium-high	36.5	35.2		35.8	0.0		39.5	40.0		39.2	31.6	
high	18.6	36.9		50.4	100.0		25.4	26.7		12.7	10.5	
Number of children in the household			**< 0.001**			0.591			0.990			**0.029**
1	18.5	19.8		8.4	0.0		16.4	17.6		23.8	35.6	
2	55.8	48.7		70.3	50.0		71.6	70.6		65.1	44.4	
3 or more	25.7	31.5		21.2	50.0		12.0	11.8		11.1	20.0	

Values are percentage for categorical and mean (standard deviation) for continuous variables. P-value was obtained using the χ^2 test was for categorical variables and Wilcoxon Rank Sum test continuous variables, bolded values indicate significance at the 0.05 level. Abbreviations: imp, imputed; na, not available; obs, observed.

© 2025 Essers E et al. *JAMA Network Open*.

eTable 6. List of variables used as predictors of participation in the covariate balancing propensity score procedure for inverse probability weighting in the two European birth cohorts.

Predictors of participation	Generation R	INMA-Gipuzkoa	INMA-Sabadell	INMA-Valencia
Adolescent biological sex	X	X	X	X
Maternal age at enrolment	X	X	X	X
Paternal age at enrolment	X	X	X	X
Maternal national origin	X	X	X	X
Paternal national origin	X	X	X	X
Maternal educational level during pregnancy	X	X	X	X
Paternal educational level during pregnancy	*	X	X	X
Family status during pregnancy	X	X	X	X
Household monthly income during pregnancy	X	**	**	**
Maternal pre-pregnancy body mass index	X	X	X	X
Paternal body mass index at enrolment	X	X	X	X
Maternal parity at pregnancy	X	X	X	X
Neighbourhood socio-economic status during pregnancy	X	X	X	X
Surrounding household greenness during pregnancy	X	X	X	X
Maternal alcohol use during pregnancy	X	X	X	X
Maternal smoking habit during pregnancy	X	X	X	X
Maternal periconceptional folic acid use during pregnancy	X	X	X	X
Adolescent week of gestation	X	X	X	X
Maternal intelligence quotient	X	*	X	X

* Not included due to more than 40% missing
** Not included due to unavailability

© 2025 Essers E et al. *JAMA Network Open*.

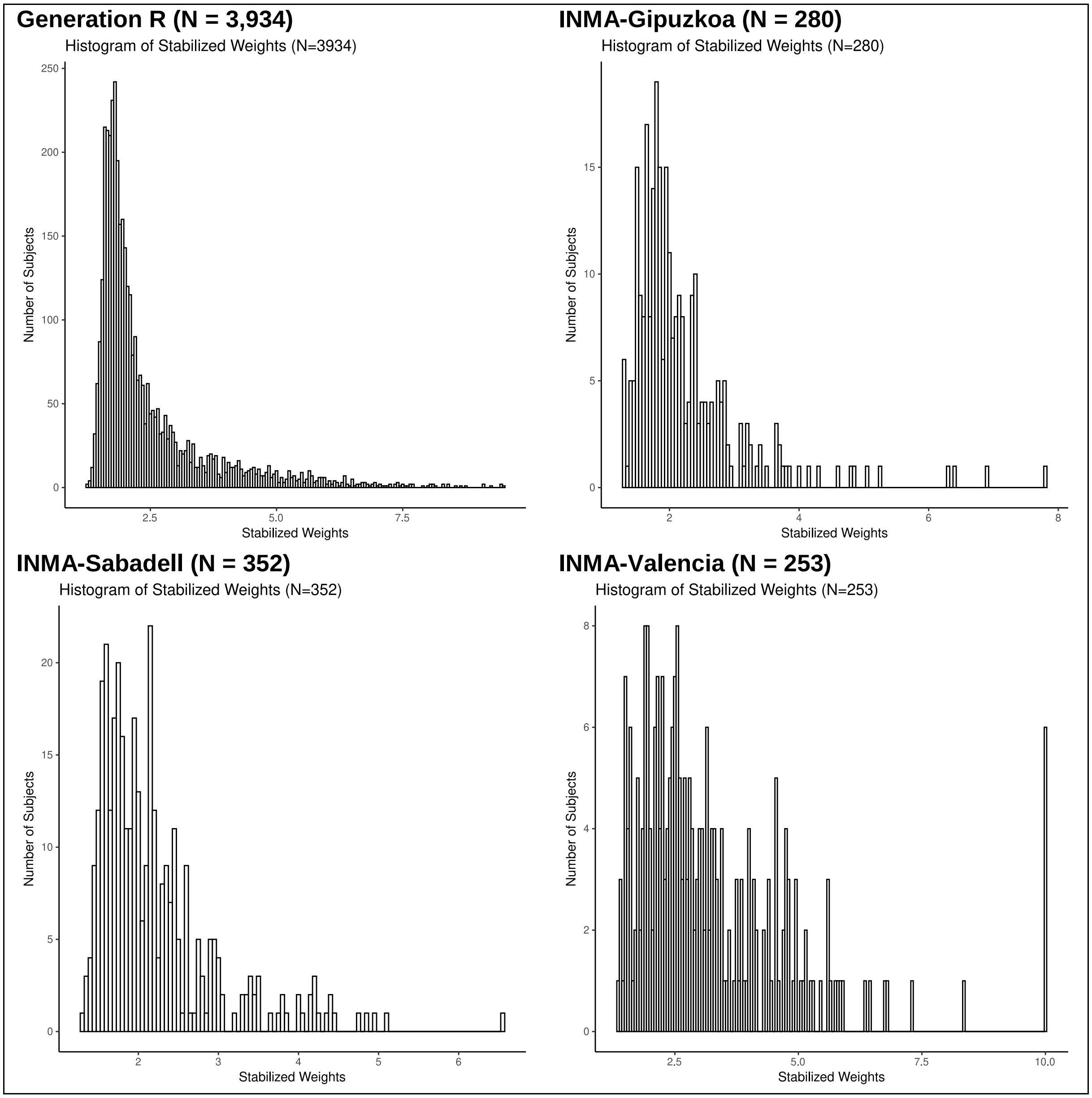

eFigure 3. Distribution of inverse probability weights for the analysis sample of each study population in the two European birth cohorts.

© 2025 Essers E et al. *JAMA Network Open*.

eAppendix 2. Details of the DLNM and its parameterization decisions

Distributed lag non-linear models (DLNM) are statistical models that analyze the lagged, potentially non-linear associations between an exposure and outcome over time[14,15]. DLNM are useful when the effect of an exposure, like ambient temperature, impact health outcomes with a delay in time, by allowing for varying strengths of the effect of different lags of time. The models estimate the exposure-response relationship while simultaneously accounting for the delayed associations between exposure and outcome (the lag-response relationship), all while considering the correlation with the time-series data (ambient temperature).

In this study, the parameterization of all DLNM was determined based on the following decisions. We first assessed the linearity of the exposure-response relationship using generalized additive models (GAM) and plotted the association between each day of temperature exposure and each outcome (i.e., each of the 56 days with the internalizing, externalizing, and attention problems score in Generation R, and INMA-Gipuzkoa, -Sabadell, and -Valencia = 672 GAM plots). The plots revealed predominantly non-linear associations and the exposure-response was therefore modelled non-linearly. We tested various knot placements in the exposure-response relationship (i.e., 25th and 75th or 10th and 90th percentiles) and in the lag-response relationship (i.e., linear or natural cubic spline with an intercept and i) one knot at lag 5.5 (median lag on the log-scale), iii) one knot at lag 28.5 (median lag), or iii) two knots at lags 19.3 and 37.7 (equally spaced)). We assessed the knot placements by evaluating the model fit by checking the Akaike Information Criterion (AIC), a common validation method in the literature.[15] In total we tested 48 models per outcome (three lag periods * two cohorts * two exposure-response modelling choices * four lag-response modelling choices). For the lag-response relationship, the AIC was almost always the lowest for the linear approach, although values differed minimally. Thus, we chose to model the lag-response relationship linearly. Subsequently, because the AICs differed extremely little between the models using the 25th/75th or 10th/90th percentiles we decided to model the exposure-response relationship setting knots at the 25th and 75th percentile, following previous literature using the same temperature data in INMA and/or Generation R.[6,7]

Next, we determined the values of the reference temperatures for the final plots of the main analysis. We calculated an optimum temperature between the 5th and 95th percentile for all three lag periods (i.e., two weeks, one month and two moths), all outcomes, and each cohort/region separately (see eTable 5). The optimum temperature is the temperature where minimal adverse effects are observed (i.e., temperature with the lowest problem score). Since we are showing the main results for three lag periods, we wanted to ease interpretation and ensure the same reference temperature for each outcome. Thus, we averaged the temperatures for the three exposure periods for Generation R and for the three exposure periods and three regions for INMA and used those as the final reference temperatures in the main analyses.

Finally, in the INMA country-level analysis, we tested the heterogeneity that might be introduced by climatological factors by using a multi-variate meta-regression and including region-specific average and range of temperature as meta-predictors. We determined the average and range of temperature of the two climatic regions representing our study population (subtropical maritime for INMA-Gipuzkoa and subtropical continental for INMA-Sabadell and -Valencia).[16] We added these climatological factors as fixed effects in models including all possible combinations (neither, average temperature, range of temperature, or both) and tested the effects through a multivariate Wald test and I^2 statistic.[17] For all models, excluding both climatological factors yielded the best model fit and thus no meta-predictors were added in the final meta-analytical models for the INMA analyses.

© 2025 Essers E et al. *JAMA Network Open*.

eTable 7. Optimum temperatures associated with the lowest outcome score in the two European birth cohorts

	Lag period-, outcome- and cohort/region-specific optimum temperatures																	
	Two-week lag period						One-month lag period						Two-month lag period					
	Internalizing Problems		Externalizing Problems		Attention Problems		Internalizing Problems		Externalizing Problems		Attention Problems		Internalizing Problems		Externalizing Problems		Attention Problems	
	ºC	Perc.	ºC	Perc.	ºC	Perc.	ºC	Perc.	ºC	Perc.	ºC	Perc.	ºC	Perc.	ºC	Perc.	ºC	Perc.
Generation R	19.2	79	5.2	13	2.3	5	18.7	76	10.0	40	11.9	48	18.3	74	22.8	95	16.8	66
INMA																		
Gipuzkoa	16.8	77	14.0	53	9.4	5	9.6	12	11.6	30	10.6	21	9	19	10.0	27	6.7	5
Sabadell	8.0	5	8.0	5	8.0	5	7.8	5	7.8	5	7.8	5	7.5	5	7.5	5	7.5	5
Valencia	9.3	5	27.5	95	11.3	11	13	21	27.0	95	12.2	16	12.1	15	12.9	20	13	21

	Outcome- and cohort-specific averaged reference temperatures					
	Averaged between the two-week, one-month, and two-month lag periods*					
	Internalizing Problems		Externalizing Problems		Attention Problems	
	ºC	Perc.	ºC	Perc.	ºC	Perc.
Generation R	18.7	76	12.7	51	10.5	41
INMA						
Two-week		11		41		8
One-month	10.3	12	14.1	42	9.7	9
Two-month		12		42		12

Orange shaded values are the optimum temperatures used for the region-specific distributed lag non-linear models for INMA and the green shaded values are the reference temperatures used to plot the final country-level results. * In INMA but not Generation R, the percentiles corresponding to the absolute temperature used in the R-code for the meta-analysis differ slightly for the lag periods.

© 2025 Essers E et al. *JAMA Network Open*.

eTable 8. Cumulative associations between ambient temperature exposure between percentiles 5 to 95 two weeks, one month, and two months prior to Child Behavioural Checklist assessment and internalizing, externalizing, or attention problems in Generation R

Two-week lag period

		Internalizing Problems			Externalizing Problems			Attention Problems		
Perc.	Temp. (ºC)	Coef.	95% CI		Coef.	95% CI		Coef.	95% CI	
5	2.3	0.24	-0.14	0.62	-0.09	-0.37	0.18	-0.05	-0.22	0.13
6	2.8	0.26	-0.12	0.64	-0.10	-0.36	0.16	-0.04	-0.20	0.12
7	3.4	0.29	-0.09	0.67	-0.10	-0.35	0.14	-0.04	-0.18	0.11
8	3.8	0.31	-0.07	0.68	-0.11	-0.35	0.13	-0.04	-0.17	0.10
9	4.1	0.32	-0.06	0.69	-0.11	-0.34	0.13	-0.03	-0.16	0.09
10	4.4	0.33	-0.05	0.70	-0.11	-0.34	0.12	-0.03	-0.15	0.09
11	4.6	0.33	-0.04	0.71	-0.11	-0.34	0.12	-0.03	-0.15	0.09
12	4.9	0.34	-0.03	0.71	-0.11	-0.33	0.11	-0.03	-0.14	0.08
13	5.2	0.35	-0.02	0.72	-0.11	-0.33	0.11	-0.03	-0.13	0.08
14	5.4	0.36	-0.01	0.73	-0.11	-0.32	0.10	-0.02	-0.13	0.08
15	5.7	0.36	-0.01	0.73	-0.11	-0.32	0.10	-0.02	-0.12	0.08
16	5.9	0.36	-0.00	0.73	-0.11	-0.32	0.10	-0.02	-0.11	0.07
17	6.1	**0.37**	**0.00**	**0.73**	-0.11	-0.31	0.09	-0.02	-0.11	0.07
18	6.3	**0.37**	**0.01**	**0.73**	-0.11	-0.31	0.09	-0.02	-0.11	0.07
19	6.5	**0.37**	**0.01**	**0.73**	-0.11	-0.30	0.09	-0.02	-0.10	0.07
20	6.7	**0.37**	**0.01**	**0.73**	-0.11	-0.30	0.09	-0.02	-0.10	0.06
21	6.9	**0.37**	**0.02**	**0.73**	-0.11	-0.30	0.08	-0.01	-0.09	0.06
22	7.1	**0.37**	**0.02**	**0.73**	-0.10	-0.29	0.08	-0.01	-0.09	0.06
23	7.2	**0.37**	**0.02**	**0.73**	-0.10	-0.29	0.08	-0.01	-0.08	0.06
24	7.4	**0.37**	**0.02**	**0.72**	-0.10	-0.28	0.08	-0.01	-0.08	0.06
25	7.5	**0.37**	**0.03**	**0.72**	-0.10	-0.28	0.08	-0.01	-0.08	0.05
26	7.7	**0.37**	**0.03**	**0.71**	-0.10	-0.27	0.08	-0.01	-0.07	0.05
27	7.9	**0.37**	**0.03**	**0.71**	-0.10	-0.27	0.07	-0.01	-0.07	0.05
28	8.0	**0.37**	**0.03**	**0.70**	-0.09	-0.26	0.07	-0.01	-0.06	0.05
29	8.2	**0.36**	**0.03**	**0.70**	-0.09	-0.25	0.07	-0.01	-0.06	0.04
30	8.4	**0.36**	**0.03**	**0.69**	-0.09	-0.25	0.07	-0.01	-0.05	0.04
31	8.5	**0.36**	**0.03**	**0.68**	-0.09	-0.24	0.07	-0.01	-0.05	0.04
32	8.7	**0.36**	**0.03**	**0.68**	-0.09	-0.23	0.06	-0.01	-0.05	0.04
33	8.8	**0.35**	**0.03**	**0.67**	-0.08	-0.23	0.06	-0.01	-0.04	0.03
34	9.0	**0.35**	**0.03**	**0.66**	-0.08	-0.22	0.06	0.00	-0.04	0.03
35	9.1	**0.34**	**0.03**	**0.66**	-0.08	-0.21	0.06	0.00	-0.03	0.03
36	9.3	**0.34**	**0.03**	**0.65**	-0.07	-0.20	0.06	0.00	-0.03	0.02
37	9.5	**0.34**	**0.03**	**0.64**	-0.07	-0.20	0.05	0.00	-0.02	0.02
38	9.6	**0.33**	**0.03**	**0.63**	-0.07	-0.19	0.05	0.00	-0.02	0.02
39	9.8	**0.32**	**0.03**	**0.61**	-0.06	-0.18	0.05	0.00	-0.01	0.01
40	10.0	**0.32**	**0.03**	**0.60**	-0.06	-0.16	0.04	0.00	-0.01	0.01
41	10.3	**0.31**	**0.03**	**0.59**	-0.06	-0.15	0.04	Ref.	Ref.	Ref.
42	10.5	**0.30**	**0.03**	**0.57**	-0.05	-0.14	0.04	0.00	-0.01	0.01
43	10.8	**0.29**	**0.03**	**0.55**	-0.05	-0.12	0.03	0.00	-0.01	0.02
44	11.0	**0.28**	**0.03**	**0.53**	-0.04	-0.11	0.03	0.00	-0.02	0.02
45	11.2	**0.27**	**0.03**	**0.51**	-0.03	-0.09	0.03	0.00	-0.03	0.03
46	11.5	**0.26**	**0.02**	**0.50**	-0.03	-0.08	0.02	0.00	-0.03	0.04
47	11.7	**0.25**	**0.02**	**0.48**	-0.02	-0.07	0.02	0.00	-0.04	0.05
48	11.9	**0.24**	**0.02**	**0.46**	-0.02	-0.05	0.01	0.00	-0.04	0.05
49	12.1	**0.23**	**0.02**	**0.44**	-0.01	-0.04	0.01	0.00	-0.05	0.06
50	12.4	**0.22**	**0.02**	**0.42**	-0.01	-0.02	0.01	0.01	-0.06	0.07

		Internalizing Problems			Externalizing Problems			Attention Problems		
Perc.	Temp. (ºC)	Coef.	95% CI		Coef.	95% CI		Coef.	95% CI	
51	12.7	**0.20**	**0.01**	**0.39**	Ref.	Ref.	Ref.	0.01	-0.07	0.08
52	13.1	**0.19**	**0.01**	**0.36**	0.01	-0.01	0.02	0.01	-0.08	0.09
53	13.4	**0.17**	**0.01**	**0.34**	0.01	-0.01	0.04	0.01	-0.09	0.10
54	13.7	**0.16**	**0.00**	**0.31**	0.02	-0.02	0.06	0.01	-0.10	0.11
55	14.0	**0.14**	**0.00**	**0.29**	0.03	-0.02	0.08	0.01	-0.10	0.12
56	14.3	0.13	-0.00	0.26	0.04	-0.03	0.10	0.01	-0.11	0.13
57	14.5	0.12	-0.00	0.25	0.04	-0.03	0.11	0.01	-0.12	0.14
58	14.8	0.11	-0.01	0.23	0.05	-0.03	0.13	0.01	-0.12	0.15
59	15.1	0.09	-0.01	0.20	0.05	-0.04	0.15	0.01	-0.13	0.16
60	15.5	0.08	-0.01	0.17	0.06	-0.05	0.17	0.01	-0.14	0.17
61	15.8	0.07	-0.01	0.15	0.07	-0.05	0.18	0.01	-0.15	0.18
62	16.0	0.06	-0.02	0.14	0.07	-0.05	0.19	0.01	-0.15	0.18
63	16.2	0.05	-0.02	0.12	0.07	-0.06	0.20	0.01	-0.16	0.19
64	16.4	0.05	-0.02	0.11	0.08	-0.06	0.22	0.02	-0.16	0.19
65	16.6	0.04	-0.02	0.10	0.08	-0.06	0.23	0.02	-0.17	0.20
66	16.8	0.03	-0.02	0.08	0.09	-0.06	0.24	0.02	-0.17	0.20
67	17.0	0.03	-0.02	0.07	0.09	-0.07	0.24	0.02	-0.18	0.21
68	17.2	0.02	-0.02	0.06	0.09	-0.07	0.25	0.02	-0.18	0.21
69	17.4	0.02	-0.02	0.05	0.09	-0.07	0.26	0.02	-0.18	0.22
70	17.6	0.01	-0.02	0.04	0.10	-0.07	0.27	0.02	-0.19	0.22
71	17.8	0.01	-0.01	0.04	0.10	-0.08	0.27	0.02	-0.19	0.23
72	18.0	0.01	-0.01	0.03	0.10	-0.08	0.28	0.02	-0.19	0.23
73	18.2	0.00	-0.01	0.02	0.10	-0.08	0.29	0.02	-0.19	0.23
74	18.4	0.00	-0.01	0.01	0.10	-0.08	0.29	0.02	-0.19	0.24
75	18.5	0.00	-0.00	0.01	0.11	-0.09	0.30	0.02	-0.20	0.24
76	18.7	Ref.	Ref.	Ref.	0.11	-0.09	0.30	0.02	-0.20	0.24
77	18.9	0.00	-0.01	0.00	0.11	-0.09	0.31	0.02	-0.20	0.25
78	19.1	0.00	-0.01	0.01	0.11	-0.09	0.31	0.02	-0.20	0.25
79	19.2	0.00	-0.01	0.01	0.11	-0.09	0.31	0.02	-0.20	0.25
80	19.4	0.00	-0.02	0.02	0.11	-0.10	0.32	0.03	-0.20	0.25
81	19.5	0.00	-0.02	0.02	0.11	-0.10	0.32	0.03	-0.20	0.25
82	19.7	0.00	-0.03	0.03	0.11	-0.10	0.32	0.03	-0.20	0.26
83	19.8	0.00	-0.03	0.03	0.11	-0.10	0.32	0.03	-0.20	0.26
84	20.0	0.00	-0.03	0.04	0.11	-0.11	0.33	0.03	-0.20	0.26
85	20.2	0.01	-0.04	0.05	0.11	-0.11	0.33	0.03	-0.20	0.26
86	20.4	0.01	-0.04	0.06	0.11	-0.11	0.33	0.03	-0.20	0.26
87	20.6	0.01	-0.04	0.07	0.11	-0.11	0.33	0.03	-0.20	0.27
88	20.8	0.02	-0.05	0.08	0.11	-0.12	0.33	0.03	-0.20	0.27
89	21.0	0.02	-0.05	0.09	0.11	-0.12	0.34	0.03	-0.20	0.27
90	21.1	0.03	-0.05	0.10	0.11	-0.12	0.34	0.03	-0.20	0.27
91	21.4	0.03	-0.05	0.12	0.11	-0.13	0.34	0.04	-0.20	0.28
92	21.7	0.04	-0.06	0.14	0.10	-0.14	0.34	0.04	-0.20	0.28
93	22.0	0.05	-0.06	0.16	0.10	-0.15	0.35	0.04	-0.20	0.28
94	22.4	0.07	-0.06	0.19	0.10	-0.16	0.35	0.04	-0.20	0.29
95	22.8	0.08	-0.07	0.23	0.09	-0.17	0.35	0.04	-0.21	0.29

Coefficients were obtained from distributed lag non-linear models and are estimated as the point change in the square-root transformed outcome score at each temperature of the lag period-specific temperature distribution between percentile 5 and 95, respective to the corresponding reference temperature (indicated at the temperature with Ref.). Bold values indicate associations that are significant at the 0.05 level and grey shaded values indicate associations that survived correction for multiple testing (P-value < 0.025). Distributed lag non-linear models were adjusted for age of adolescent at and month of Child Behavioural Checklist assessment, adolescent sex, parental age, national origin, educational level, employment status, and family status; monthly household income, number of children in the household, residential surrounding greenness, and neighbourhood socio-economic status. Abbreviations: CI, confidence interval; Coef., coefficient; Perc., percentile; Temp., temperature.

© 2025 Essers E et al. *JAMA Network Open*.

eTable 8, continued. Cumulative associations between ambient temperature exposure between percentiles 5 to 95 two weeks, one month, and two months prior to Child Behavioural Checklist assessment and internalizing, externalizing, or attention problems in Generation R

One-month lag period

		Internalizing Problems			Externalizing Problems			Attention Problems		
Perc.	Temp. (ºC)	Coef.	95% CI		Coef.	95% CI		Coef.	95% CI	
5	2.5	0.39	-0.10	0.87	0.11	-0.24	0.47	0.17	-0.06	0.40
6	3.0	0.40	-0.08	0.88	0.10	-0.24	0.43	0.15	-0.05	0.36
7	3.5	0.41	-0.07	0.89	0.08	-0.24	0.40	0.13	-0.05	0.32
8	3.9	0.42	-0.06	0.89	0.07	-0.24	0.38	0.12	-0.05	0.30
9	4.2	0.42	-0.05	0.90	0.06	-0.24	0.37	0.11	-0.05	0.28
10	4.5	0.43	-0.05	0.90	0.06	-0.24	0.35	0.11	-0.05	0.26
11	4.7	0.43	-0.04	0.90	0.05	-0.24	0.34	0.10	-0.05	0.25
12	5.0	0.43	-0.04	0.90	0.04	-0.24	0.33	0.09	-0.05	0.23
13	5.2	0.43	-0.03	0.90	0.04	-0.24	0.32	0.08	-0.05	0.22
14	5.5	0.43	-0.03	0.90	0.03	-0.24	0.31	0.08	-0.05	0.21
15	5.8	0.43	-0.03	0.90	0.03	-0.24	0.30	0.07	-0.06	0.19
16	6.0	0.43	-0.03	0.90	0.02	-0.24	0.29	0.06	-0.06	0.18
17	6.1	0.43	-0.03	0.89	0.02	-0.24	0.28	0.06	-0.06	0.18
18	6.3	0.43	-0.03	0.89	0.02	-0.24	0.27	0.06	-0.06	0.17
19	6.5	0.43	-0.02	0.89	0.02	-0.24	0.27	0.05	-0.06	0.16
20	6.7	0.43	-0.02	0.88	0.01	-0.23	0.26	0.05	-0.06	0.15
21	6.9	0.43	-0.02	0.88	0.01	-0.23	0.25	0.04	-0.05	0.14
22	7.1	0.42	-0.02	0.87	0.01	-0.23	0.25	0.04	-0.05	0.13
23	7.2	0.42	-0.02	0.86	0.01	-0.23	0.24	0.04	-0.05	0.13
24	7.4	0.42	-0.02	0.86	0.00	-0.23	0.23	0.03	-0.05	0.12
25	7.6	0.41	-0.02	0.85	0.00	-0.22	0.23	0.03	-0.05	0.11
26	7.7	0.41	-0.02	0.84	0.00	-0.22	0.22	0.03	-0.05	0.11
27	7.9	0.41	-0.02	0.84	0.00	-0.22	0.22	0.03	-0.05	0.10
28	8.1	0.40	-0.02	0.83	0.00	-0.21	0.21	0.02	-0.05	0.09
29	8.2	0.40	-0.02	0.82	0.00	-0.21	0.20	0.02	-0.04	0.08
30	8.4	0.39	-0.02	0.81	-0.01	-0.20	0.19	0.02	-0.04	0.08
31	8.5	0.39	-0.02	0.80	-0.01	-0.20	0.19	0.02	-0.04	0.07
32	8.7	0.38	-0.02	0.79	-0.01	-0.20	0.18	0.01	-0.04	0.07
33	8.8	0.38	-0.03	0.78	-0.01	-0.19	0.18	0.01	-0.03	0.06
34	9.0	0.37	-0.03	0.77	-0.01	-0.19	0.17	0.01	-0.03	0.05
35	9.1	0.37	-0.03	0.76	-0.01	-0.18	0.16	0.01	-0.03	0.05
36	9.3	0.36	-0.03	0.75	-0.01	-0.18	0.16	0.01	-0.03	0.04
37	9.4	0.35	-0.03	0.74	-0.01	-0.17	0.15	0.01	-0.02	0.03
38	9.6	0.35	-0.03	0.72	-0.01	-0.16	0.14	0.00	-0.02	0.03
39	9.8	0.34	-0.03	0.71	-0.01	-0.15	0.13	0.00	-0.01	0.02
40	10.0	0.33	-0.03	0.69	-0.01	-0.14	0.12	0.00	-0.01	0.01
41	10.3	0.32	-0.03	0.67	-0.01	-0.13	0.11	Ref.	Ref.	Ref.
42	10.5	0.31	-0.03	0.65	-0.01	-0.13	0.10	0.00	-0.01	0.01
43	10.7	0.30	-0.03	0.63	-0.01	-0.11	0.09	0.00	-0.02	0.02
44	11.0	0.29	-0.03	0.60	-0.01	-0.10	0.08	0.00	-0.03	0.02
45	11.2	0.27	-0.04	0.58	-0.01	-0.09	0.07	0.00	-0.04	0.03
46	11.5	0.26	-0.04	0.56	-0.01	-0.07	0.06	0.00	-0.05	0.04
47	11.7	0.25	-0.04	0.54	-0.01	-0.06	0.05	0.00	-0.06	0.05
48	11.9	0.24	-0.04	0.52	-0.01	-0.05	0.04	0.00	-0.07	0.06
49	12.1	0.23	-0.04	0.49	0.00	-0.04	0.03	0.00	-0.08	0.07
50	12.4	0.21	-0.04	0.47	0.00	-0.02	0.02	0.00	-0.09	0.08

		Internalizing Problems			Externalizing Problems			Attention Problems		
Perc.	Temp. (ºC)	Coef.	95% CI		Coef.	95% CI		Coef.	95% CI	
51	12.7	0.20	-0.04	0.43	Ref.	Ref.	Ref.	0.00	-0.10	0.09
52	13.0	0.18	-0.04	0.40	0.00	-0.01	0.02	0.00	-0.11	0.10
53	13.3	0.17	-0.04	0.37	0.00	-0.03	0.04	0.00	-0.12	0.12
54	13.7	0.15	-0.04	0.34	0.01	-0.04	0.06	0.00	-0.13	0.13
55	14.0	0.13	-0.05	0.31	0.01	-0.05	0.07	0.00	-0.14	0.15
56	14.3	0.12	-0.05	0.29	0.01	-0.07	0.09	0.00	-0.15	0.16
57	14.5	0.11	-0.05	0.27	0.01	-0.08	0.10	0.00	-0.16	0.17
58	14.7	0.10	-0.05	0.24	0.02	-0.09	0.12	0.01	-0.17	0.18
59	15.1	0.08	-0.05	0.21	0.02	-0.10	0.14	0.01	-0.18	0.20
60	15.5	0.07	-0.05	0.18	0.02	-0.11	0.16	0.01	-0.19	0.21
61	15.7	0.06	-0.05	0.16	0.03	-0.12	0.17	0.01	-0.19	0.22
62	16.0	0.05	-0.04	0.14	0.03	-0.13	0.18	0.02	-0.20	0.23
63	16.2	0.04	-0.04	0.13	0.03	-0.14	0.19	0.02	-0.20	0.24
64	16.4	0.04	-0.04	0.11	0.03	-0.14	0.21	0.02	-0.21	0.25
65	16.6	0.03	-0.04	0.10	0.03	-0.15	0.22	0.02	-0.21	0.26
66	16.8	0.03	-0.04	0.09	0.03	-0.16	0.22	0.02	-0.21	0.26
67	17.0	0.02	-0.04	0.08	0.04	-0.16	0.23	0.03	-0.22	0.27
68	17.2	0.02	-0.03	0.07	0.04	-0.17	0.24	0.03	-0.22	0.28
69	17.4	0.01	-0.03	0.06	0.04	-0.17	0.25	0.03	-0.22	0.29
70	17.6	0.01	-0.03	0.05	0.04	-0.18	0.26	0.03	-0.22	0.29
71	17.8	0.01	-0.03	0.04	0.04	-0.18	0.26	0.04	-0.23	0.30
72	18.0	0.00	-0.02	0.03	0.04	-0.18	0.27	0.04	-0.23	0.30
73	18.2	0.00	-0.02	0.02	0.04	-0.19	0.28	0.04	-0.23	0.31
74	18.4	0.00	-0.01	0.01	0.05	-0.19	0.28	0.04	-0.23	0.32
75	18.5	0.00	-0.01	0.01	0.05	-0.19	0.29	0.05	-0.23	0.32
76	18.7	Ref.	Ref.	Ref.	0.05	-0.20	0.29	0.05	-0.23	0.33
77	18.9	0.00	-0.01	0.01	0.05	-0.20	0.30	0.05	-0.23	0.33
78	19.0	0.00	-0.01	0.01	0.05	-0.20	0.30	0.05	-0.23	0.33
79	19.2	0.00	-0.02	0.02	0.05	-0.21	0.30	0.05	-0.23	0.34
80	19.4	0.00	-0.02	0.03	0.05	-0.21	0.31	0.06	-0.23	0.34
81	19.5	0.01	-0.02	0.04	0.05	-0.21	0.31	0.06	-0.23	0.34
82	19.7	0.01	-0.03	0.04	0.05	-0.21	0.31	0.06	-0.23	0.35
83	19.8	0.01	-0.03	0.05	0.05	-0.21	0.32	0.06	-0.23	0.35
84	20.0	0.01	-0.03	0.06	0.05	-0.22	0.32	0.06	-0.23	0.35
85	20.2	0.02	-0.04	0.07	0.05	-0.22	0.32	0.07	-0.22	0.36
86	20.4	0.02	-0.04	0.09	0.05	-0.22	0.33	0.07	-0.22	0.36
87	20.6	0.03	-0.04	0.10	0.05	-0.22	0.33	0.07	-0.22	0.37
88	20.7	0.03	-0.05	0.11	0.05	-0.23	0.33	0.07	-0.22	0.37
89	20.9	0.04	-0.05	0.13	0.05	-0.23	0.34	0.08	-0.22	0.37
90	21.1	0.05	-0.05	0.15	0.05	-0.23	0.34	0.08	-0.22	0.38
91	21.3	0.05	-0.06	0.16	0.05	-0.24	0.34	0.08	-0.21	0.38
92	21.7	0.07	-0.06	0.19	0.05	-0.24	0.35	0.09	-0.21	0.39
93	22.0	0.08	-0.06	0.22	0.05	-0.25	0.36	0.09	-0.21	0.39
94	22.3	0.10	-0.07	0.26	0.05	-0.26	0.36	0.10	-0.21	0.40
95	22.7	0.12	-0.07	0.31	0.05	-0.27	0.37	0.10	-0.20	0.41

Coefficients were obtained from distributed lag non-linear models and are estimated as the point change in the square-root transformed outcome score at each temperature of the lag period-specific temperature distribution between percentile 5 and 95, respective to the corresponding reference temperature (indicated at the temperature with Ref.). Bold values indicate associations that are significant at the 0.05 level and grey shaded values indicate associations that survived correction for multiple testing (P-value < 0.025). Distributed lag non-linear models were adjusted for age of adolescent at and month of Child Behavioural Checklist assessment, adolescent sex, parental age, national origin, educational level, employment status, and family status; monthly household income, number of children in the household, residential surrounding greenness, and neighbourhood socio-economic status. Abbreviations: CI, confidence interval; Coef., coefficient; Perc., percentile; Temp., temperature.

© 2025 Essers E et al. *JAMA Network Open*.

eTable 8, continued. Cumulative associations between ambient temperature exposure between percentiles 5 to 95 two weeks, one month, and two months prior to Child Behavioural Checklist assessment and internalizing, externalizing, or attention problems in Generation R

		Two-month lag period																			
		Internalizing Problems			Externalizing Problems			Attention Problems					Internalizing Problems			Externalizing Problems			Attention Problems		
Perc.	Temp. (ºC)	Coef.	95% CI		Coef.	95% CI		Coef.	95% CI		Perc.	Temp. (ºC)	Coef.	95% CI		Coef.	95% CI		Coef.	95% CI	
5	2.7	**0.69**	**0.09**	**1.30**	0.04	-0.45	0.53	0.01	-0.32	0.35	51	12.7	**0.33**	**0.04**	**0.62**	Ref.	Ref.	Ref.	-0.04	-0.15	0.08
6	3.3	**0.72**	**0.13**	**1.31**	0.04	-0.41	0.48	0.02	-0.28	0.31	52	13.0	**0.30**	**0.03**	**0.57**	0.00	-0.02	0.02	-0.04	-0.17	0.09
7	3.7	**0.73**	**0.15**	**1.31**	0.03	-0.40	0.46	0.02	-0.25	0.29	53	13.3	**0.27**	**0.02**	**0.53**	0.00	-0.04	0.04	-0.04	-0.18	0.10
8	4.0	**0.74**	**0.16**	**1.31**	0.03	-0.38	0.44	0.02	-0.23	0.28	54	13.6	**0.25**	**0.01**	**0.49**	0.00	-0.05	0.06	-0.05	-0.20	0.11
9	4.3	**0.75**	**0.17**	**1.32**	0.02	-0.37	0.42	0.03	-0.21	0.27	55	13.9	0.22	-0.00	0.44	0.00	-0.08	0.08	-0.05	-0.22	0.12
10	4.5	**0.75**	**0.18**	**1.32**	0.02	-0.36	0.41	0.03	-0.20	0.26	56	14.2	0.20	-0.01	0.40	0.00	-0.09	0.10	-0.06	-0.24	0.13
11	4.7	**0.75**	**0.19**	**1.32**	0.02	-0.36	0.39	0.03	-0.19	0.25	57	14.4	0.18	-0.02	0.37	0.00	-0.11	0.11	-0.06	-0.25	0.14
12	5.0	**0.76**	**0.20**	**1.32**	0.02	-0.35	0.38	0.03	-0.17	0.23	58	14.7	0.16	-0.02	0.34	0.00	-0.12	0.13	-0.06	-0.27	0.15
13	5.3	**0.76**	**0.20**	**1.32**	0.01	-0.34	0.37	0.03	-0.16	0.22	59	15.0	0.13	-0.03	0.29	0.00	-0.14	0.15	-0.06	-0.29	0.16
14	5.5	**0.76**	**0.20**	**1.32**	0.01	-0.33	0.36	0.03	-0.15	0.22	60	15.4	0.11	-0.04	0.25	0.00	-0.16	0.17	-0.07	-0.30	0.17
15	5.7	**0.76**	**0.21**	**1.32**	0.01	-0.33	0.35	0.03	-0.14	0.21	61	15.7	0.09	-0.04	0.22	0.00	-0.18	0.18	-0.07	-0.32	0.18
16	6.0	**0.76**	**0.21**	**1.31**	0.01	-0.32	0.34	0.03	-0.13	0.20	62	15.9	0.07	-0.05	0.19	0.00	-0.19	0.20	-0.07	-0.33	0.19
17	6.1	**0.76**	**0.21**	**1.31**	0.01	-0.32	0.33	0.03	-0.12	0.19	63	16.1	0.06	-0.05	0.17	0.00	-0.20	0.21	-0.07	-0.34	0.20
18	6.3	**0.76**	**0.21**	**1.30**	0.00	-0.31	0.32	0.03	-0.12	0.18	64	16.3	0.05	-0.05	0.15	0.00	-0.21	0.22	-0.07	-0.35	0.20
19	6.5	**0.75**	**0.21**	**1.30**	0.00	-0.31	0.31	0.03	-0.11	0.18	65	16.6	0.04	-0.05	0.13	0.00	-0.22	0.23	-0.07	-0.35	0.21
20	6.7	**0.75**	**0.21**	**1.29**	0.00	-0.30	0.31	0.03	-0.10	0.17	66	16.8	0.03	-0.05	0.11	0.00	-0.23	0.23	-0.07	-0.36	0.22
21	6.9	**0.74**	**0.21**	**1.28**	0.00	-0.30	0.30	0.03	-0.10	0.16	67	16.9	0.02	-0.05	0.10	0.00	-0.24	0.24	-0.07	-0.37	0.22
22	7.1	**0.74**	**0.21**	**1.27**	0.00	-0.29	0.29	0.03	-0.09	0.15	68	17.1	0.02	-0.05	0.08	0.00	-0.25	0.25	-0.07	-0.37	0.23
23	7.3	**0.73**	**0.20**	**1.26**	0.00	-0.29	0.28	0.03	-0.09	0.15	69	17.3	0.01	-0.05	0.07	0.00	-0.26	0.26	-0.07	-0.38	0.24
24	7.4	**0.73**	**0.20**	**1.25**	0.00	-0.28	0.28	0.03	-0.08	0.14	70	17.5	0.01	-0.04	0.06	0.00	-0.27	0.26	-0.07	-0.38	0.24
25	7.6	**0.72**	**0.20**	**1.24**	0.00	-0.28	0.27	0.03	-0.08	0.14	71	17.7	0.00	-0.04	0.04	0.00	-0.28	0.27	-0.07	-0.38	0.25
26	7.8	**0.71**	**0.20**	**1.23**	0.00	-0.27	0.26	0.03	-0.07	0.13	72	17.9	0.00	-0.03	0.03	-0.01	-0.28	0.27	-0.07	-0.39	0.25
27	7.9	**0.71**	**0.19**	**1.22**	0.00	-0.26	0.26	0.03	-0.07	0.12	73	18.1	0.00	-0.03	0.02	-0.01	-0.29	0.28	-0.07	-0.39	0.26
28	8.1	**0.70**	**0.19**	**1.20**	0.00	-0.26	0.25	0.03	-0.06	0.11	74	18.3	0.00	-0.02	0.01	-0.01	-0.30	0.28	-0.06	-0.39	0.26
29	8.3	**0.69**	**0.18**	**1.19**	0.00	-0.25	0.24	0.02	-0.06	0.11	75	18.5	0.00	-0.01	0.01	-0.01	-0.31	0.29	-0.06	-0.39	0.27
30	8.4	**0.68**	**0.18**	**1.18**	0.00	-0.24	0.24	0.02	-0.05	0.10	76	18.7	Ref.	Ref.	Ref.	-0.01	-0.31	0.29	-0.06	-0.39	0.27
31	8.6	**0.67**	**0.18**	**1.16**	0.00	-0.24	0.23	0.02	-0.05	0.09	77	18.8	0.00	-0.01	0.01	-0.01	-0.32	0.29	-0.06	-0.39	0.28
32	8.7	**0.66**	**0.17**	**1.15**	0.00	-0.23	0.22	0.02	-0.05	0.09	78	19.0	0.01	-0.01	0.02	-0.02	-0.32	0.29	-0.05	-0.39	0.28
33	8.9	**0.65**	**0.17**	**1.13**	-0.01	-0.23	0.22	0.02	-0.04	0.08	79	19.2	0.01	-0.01	0.03	-0.02	-0.33	0.30	-0.05	-0.39	0.29
34	9.0	**0.64**	**0.16**	**1.12**	-0.01	-0.22	0.21	0.02	-0.04	0.07	80	19.3	0.01	-0.02	0.05	-0.02	-0.33	0.30	-0.05	-0.39	0.29
35	9.2	**0.63**	**0.16**	**1.10**	-0.01	-0.21	0.20	0.02	-0.03	0.06	81	19.5	0.02	-0.02	0.06	-0.02	-0.34	0.30	-0.05	-0.39	0.30
36	9.3	**0.62**	**0.16**	**1.09**	-0.01	-0.20	0.19	0.01	-0.03	0.06	82	19.6	0.03	-0.02	0.07	-0.02	-0.34	0.30	-0.04	-0.39	0.30
37	9.5	**0.61**	**0.15**	**1.07**	-0.01	-0.20	0.19	0.01	-0.02	0.05	83	19.8	0.03	-0.02	0.09	-0.02	-0.35	0.30	-0.04	-0.39	0.31
38	9.7	**0.59**	**0.14**	**1.05**	-0.01	-0.19	0.18	0.01	-0.02	0.04	84	20.0	0.04	-0.03	0.11	-0.03	-0.36	0.30	-0.04	-0.38	0.31
39	9.9	**0.58**	**0.14**	**1.02**	-0.01	-0.18	0.17	0.01	-0.01	0.03	85	20.2	0.05	-0.03	0.13	-0.03	-0.36	0.30	-0.03	-0.38	0.32
40	10.1	**0.56**	**0.13**	**0.99**	0.00	-0.16	0.15	0.00	-0.01	0.01	86	20.4	0.06	-0.03	0.16	-0.03	-0.37	0.31	-0.03	-0.38	0.33
41	10.3	**0.54**	**0.12**	**0.96**	0.00	-0.15	0.14	Ref.	Ref.	Ref.	87	20.6	0.08	-0.03	0.18	-0.04	-0.38	0.31	-0.02	-0.37	0.33
42	10.5	**0.52**	**0.11**	**0.93**	0.00	-0.14	0.13	0.00	-0.01	0.01	88	20.8	0.09	-0.03	0.20	-0.04	-0.39	0.31	-0.02	-0.37	0.34
43	10.8	**0.50**	**0.10**	**0.90**	0.00	-0.12	0.11	-0.01	-0.03	0.01	89	21.0	0.10	-0.03	0.23	-0.04	-0.39	0.31	-0.01	-0.36	0.35
44	11.0	**0.48**	**0.10**	**0.86**	0.00	-0.11	0.10	-0.01	-0.04	0.02	90	21.2	0.12	-0.02	0.26	-0.05	-0.40	0.31	0.00	-0.36	0.35
45	11.3	**0.46**	**0.09**	**0.83**	0.00	-0.09	0.09	-0.01	-0.06	0.03	91	21.4	0.14	-0.02	0.30	-0.05	-0.41	0.31	0.00	-0.36	0.36
46	11.5	**0.44**	**0.08**	**0.79**	0.00	-0.08	0.07	-0.02	-0.07	0.04	92	21.7	0.17	-0.02	0.35	-0.06	-0.43	0.32	0.01	-0.35	0.37
47	11.7	**0.42**	**0.07**	**0.77**	0.00	-0.07	0.06	-0.02	-0.08	0.04	93	22.0	0.20	-0.01	0.40	-0.06	-0.45	0.32	0.02	-0.34	0.39
48	11.9	**0.40**	**0.06**	**0.74**	0.00	-0.05	0.05	-0.02	-0.10	0.05	94	22.3	0.23	-0.01	0.47	-0.07	-0.47	0.33	0.04	-0.33	0.41
49	12.1	**0.38**	**0.06**	**0.71**	0.00	-0.04	0.04	-0.03	-0.11	0.06	95	22.8	**0.28**	**0.00**	**0.56**	-0.08	-0.50	0.34	0.05	-0.32	0.43
50	12.4	**0.36**	**0.05**	**0.67**	0.00	-0.02	0.02	-0.03	-0.13	0.07											

Coefficients were obtained from distributed lag non-linear models and are estimated as the point change in the square-root transformed outcome score at each temperature of the lag period-specific temperature distribution between percentile 5 and 95, respective to the corresponding reference temperature (indicated at the temperature with Ref.). Bold values indicate associations that are significant at the 0.05 level and grey shaded values indicate associations that survived correction for multiple testing (P-value < 0.025). Distributed lag non-linear models were adjusted for age of adolescent at and month of Child Behavioural Checklist assessment, adolescent sex, parental age, national origin, educational level, employment status, and family status; monthly household income, number of children in the household, residential surrounding greenness, and neighbourhood socio-economic status. Abbreviations: CI, confidence interval; Coef., coefficient; Perc., percentile; Temp., temperature.

© 2025 Essers E et al. *JAMA Network Open*.

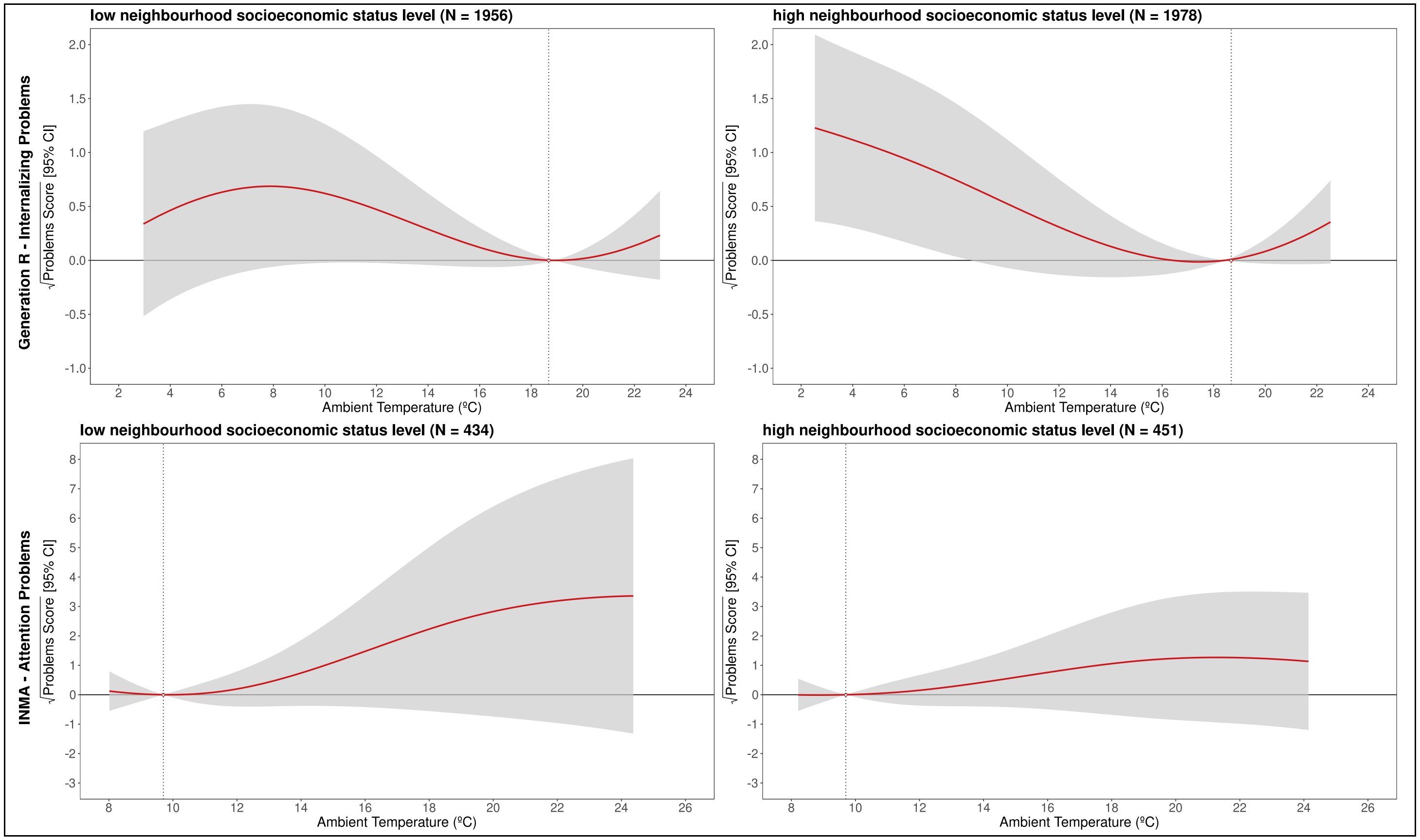

eFigure 4. Cumulative associations between temperature exposure during the two months prior to outcome assessment and internalizing problems in Generation R (top) or attention problems in INMA (bottom), split by neighbourhood socioeconomic status.

The x-axis depicts the 5th to 95th range of the country-wide temperature distribution (for Generation R 3.0 to 23.0 ºC in the low group and 2.5 to 22.5 ºC in the high group, and for INMA, 5.6 to 27.6 ºC in the low group and 5.5 to 27.3 ºC in the high group). Solid red lines represent the country-average curve, expressed as beta coefficients of the problem scores, derived from the distributed lag non-linear (Generation R) or the random-effects meta-analysis (INMA) models, with their respective 95% confidence intervals in grey. Coefficients are estimated as the change in the outcome score at each temperature of the country-wide temperature distribution respective to the corresponding reference temperature (black dotted lines). Within each cohort/region, distributed lag non-linear models were adjusted for age of adolescent at and month of Child Behavioural Checklist assessment, adolescent sex, parental age, national origin, educational level, employment status, and family status; monthly income, number of children in the household, and residential surrounding greenness.

© 2025 Essers E et al. *JAMA Network Open*.

eTable 9. Cumulative associations between ambient temperature exposure between percentiles 5 to 95 two weeks, one month, and two months prior to Child Behavioural Checklist assessment and internalizing, externalizing, or attention problems in INMA

		Two-week lag period																		
		Internalizing Problems			Externalizing Problems			Attention Problems					Internalizing Problems			Externalizing Problems			Attention Problems		
Perc.	Temp. (ºC)	Coef.	95% CI		Coef.	95% CI		Coef.	95% CI		Perc.	Temp. (ºC)	Coef.	95% CI		Coef.	95% CI		Coef.	95% CI	
5	8.9	-0.18	-0.42	0.06	-0.12	-0.86	0.63	-0.08	-0.21	0.05	51	15.2	0.30	-0.15	0.75	-0.03	-0.17	0.11	0.39	-0.06	0.84
6	9.2	-0.13	-0.30	0.04	-0.09	-0.76	0.57	-0.05	-0.12	0.03	52	15.4	0.30	-0.16	0.76	-0.04	-0.20	0.13	0.40	-0.06	0.86
7	9.5	-0.09	-0.22	0.03	-0.08	-0.68	0.53	-0.02	-0.04	0.01	53	15.6	0.31	-0.18	0.79	-0.04	-0.24	0.15	0.41	-0.06	0.89
8	9.8	-0.06	-0.15	0.02	-0.06	-0.62	0.49	0.01	0.00	0.01	54	15.8	0.31	-0.18	0.81	-0.05	-0.26	0.17	0.42	-0.06	0.90
9	10.0	-0.04	-0.08	0.01	-0.05	-0.56	0.46	0.03	-0.01	0.07	55	16.0	0.32	-0.19	0.82	-0.05	-0.29	0.19	0.43	-0.07	0.92
10	10.2	-0.01	-0.03	0.00	-0.04	-0.52	0.43	0.04	-0.02	0.11	56	16.1	0.32	-0.20	0.84	-0.06	-0.32	0.20	0.43	-0.07	0.94
11	10.3	0.00	-0.00	0.01	-0.03	-0.48	0.42	0.06	-0.03	0.14	57	16.3	0.32	-0.21	0.86	-0.06	-0.34	0.22	0.44	-0.07	0.96
12	10.5	0.02	-0.01	0.05	-0.03	-0.45	0.40	0.07	-0.03	0.18	58	16.4	0.33	-0.22	0.88	-0.07	-0.37	0.24	0.45	-0.08	0.97
13	10.7	0.04	-0.01	0.09	-0.02	-0.42	0.38	0.09	-0.04	0.21	59	16.6	0.33	-0.23	0.90	-0.07	-0.40	0.25	0.46	-0.08	0.99
14	10.8	0.06	-0.02	0.13	-0.01	-0.39	0.36	0.10	-0.04	0.25	60	16.8	0.34	-0.24	0.92	-0.08	-0.42	0.27	0.46	-0.09	1.01
15	11.0	0.07	-0.02	0.17	-0.01	-0.35	0.34	0.12	-0.05	0.28	61	16.9	0.34	-0.25	0.94	-0.08	-0.45	0.29	0.47	-0.09	1.03
16	11.2	0.09	-0.03	0.21	0.00	-0.32	0.32	0.13	-0.05	0.32	62	17.2	0.35	-0.27	0.96	-0.08	-0.48	0.31	0.48	-0.10	1.05
17	11.4	0.11	-0.03	0.24	0.00	-0.29	0.30	0.15	-0.05	0.35	63	17.3	0.36	-0.28	0.99	-0.09	-0.51	0.33	0.49	-0.10	1.07
18	11.5	0.12	-0.03	0.27	0.01	-0.28	0.29	0.16	-0.06	0.37	64	17.5	0.36	-0.29	1.01	-0.09	-0.54	0.35	0.49	-0.11	1.09
19	11.6	0.13	-0.04	0.29	0.01	-0.26	0.28	0.17	-0.06	0.39	65	17.7	0.37	-0.29	1.03	-0.10	-0.56	0.37	0.50	-0.11	1.11
20	11.7	0.14	-0.04	0.31	0.01	-0.24	0.27	0.18	-0.06	0.41	66	18.0	0.38	-0.30	1.06	-0.10	-0.59	0.39	0.51	-0.12	1.14
21	11.8	0.15	-0.04	0.33	0.01	-0.23	0.26	0.18	-0.06	0.43	67	18.1	0.39	-0.31	1.08	-0.10	-0.61	0.41	0.52	-0.12	1.16
22	11.9	0.15	-0.04	0.35	0.01	-0.22	0.25	0.19	-0.06	0.44	68	18.3	0.39	-0.31	1.10	-0.10	-0.63	0.42	0.52	-0.12	1.17
23	12.0	0.16	-0.04	0.36	0.02	-0.21	0.24	0.20	-0.06	0.46	69	18.5	0.40	-0.32	1.13	-0.10	-0.65	0.44	0.53	-0.13	1.19
24	12.1	0.17	-0.05	0.38	0.02	-0.20	0.23	0.20	-0.06	0.47	70	18.6	0.41	-0.32	1.14	-0.10	-0.67	0.46	0.54	-0.13	1.20
25	12.2	0.17	-0.05	0.40	0.02	-0.18	0.22	0.21	-0.06	0.49	71	18.7	0.42	-0.32	1.16	-0.10	-0.68	0.47	0.54	-0.13	1.22
26	12.4	0.18	-0.05	0.42	0.02	-0.17	0.20	0.22	-0.06	0.51	72	18.9	0.43	-0.32	1.18	-0.10	-0.69	0.48	0.55	-0.14	1.23
27	12.5	0.19	-0.05	0.44	0.02	-0.15	0.19	0.23	-0.06	0.53	73	19.0	0.44	-0.32	1.20	-0.10	-0.70	0.50	0.55	-0.14	1.24
28	12.6	0.20	-0.06	0.45	0.02	-0.14	0.18	0.24	-0.06	0.54	74	19.1	0.45	-0.32	1.22	-0.10	-0.71	0.51	0.56	-0.14	1.25
29	12.7	0.20	-0.06	0.47	0.02	-0.13	0.17	0.25	-0.06	0.56	75	19.4	0.46	-0.32	1.24	-0.10	-0.73	0.53	0.57	-0.14	1.27
30	12.8	0.21	-0.06	0.48	0.02	-0.12	0.16	0.26	-0.06	0.57	76	19.6	0.48	-0.32	1.27	-0.10	-0.74	0.55	0.57	-0.14	1.29
31	12.9	0.22	-0.06	0.49	0.02	-0.11	0.15	0.26	-0.06	0.58	77	19.8	0.50	-0.31	1.30	-0.09	-0.75	0.57	0.58	-0.14	1.31
32	13.0	0.22	-0.07	0.51	0.02	-0.10	0.14	0.27	-0.06	0.60	78	20.0	0.52	-0.30	1.33	-0.09	-0.76	0.59	0.59	-0.14	1.32
33	13.1	0.23	-0.07	0.52	0.02	-0.09	0.12	0.28	-0.06	0.61	79	20.2	0.54	-0.29	1.37	-0.08	-0.77	0.62	0.60	-0.14	1.34
34	13.3	0.23	-0.07	0.54	0.01	-0.08	0.11	0.28	-0.06	0.62	80	20.5	0.57	-0.27	1.40	-0.07	-0.78	0.65	0.61	-0.14	1.36
35	13.4	0.24	-0.07	0.55	0.01	-0.07	0.09	0.29	-0.06	0.64	81	20.8	0.59	-0.25	1.44	-0.06	-0.79	0.68	0.62	-0.14	1.38
36	13.5	0.24	-0.08	0.56	0.01	-0.06	0.08	0.30	-0.06	0.65	82	21.0	0.62	-0.24	1.48	-0.05	-0.79	0.70	0.63	-0.14	1.40
37	13.6	0.25	-0.08	0.58	0.01	-0.05	0.07	0.31	-0.06	0.67	83	21.2	0.65	-0.22	1.51	-0.03	-0.80	0.73	0.64	-0.13	1.42
38	13.7	0.25	-0.08	0.59	0.01	-0.04	0.05	0.31	-0.05	0.68	84	21.6	0.69	-0.19	1.57	-0.01	-0.81	0.78	0.65	-0.13	1.44
39	13.8	0.25	-0.09	0.60	0.01	-0.03	0.04	0.32	-0.05	0.69	85	21.9	0.73	-0.15	1.62	0.01	-0.81	0.83	0.67	-0.13	1.46
40	13.9	0.26	-0.09	0.61	0.00	-0.02	0.03	0.32	-0.05	0.70	86	22.1	0.77	-0.13	1.67	0.03	-0.82	0.87	0.68	-0.13	1.48
41	14.0	0.26	-0.09	0.62	0.00	-0.01	0.01	0.33	-0.05	0.71	87	22.4	0.80	-0.11	1.71	0.04	-0.83	0.91	0.69	-0.13	1.50
42	14.1	0.27	-0.10	0.63	0.00	-0.00	0.00	0.33	-0.05	0.72	88	22.6	0.83	-0.08	1.75	0.06	-0.83	0.95	0.70	-0.13	1.52
43	14.2	0.27	-0.10	0.64	0.00	-0.02	0.01	0.34	-0.05	0.73	89	22.9	0.88	-0.05	1.82	0.09	-0.85	1.02	0.71	-0.13	1.55
44	14.3	0.27	-0.11	0.65	0.00	-0.03	0.02	0.35	-0.05	0.74	90	23.2	0.93	-0.02	1.88	0.11	-0.86	1.09	0.72	-0.13	1.57
45	14.4	0.27	-0.11	0.66	-0.01	-0.04	0.03	0.35	-0.05	0.75	91	23.6	**0.99**	**0.02**	**1.96**	0.14	-0.88	1.17	0.74	-0.13	1.61
46	14.5	0.28	-0.12	0.67	-0.01	-0.06	0.04	0.36	-0.05	0.77	92	23.9	**1.05**	**0.05**	**2.05**	0.18	-0.91	1.28	0.75	-0.14	1.65
47	14.6	0.28	-0.12	0.68	-0.01	-0.08	0.05	0.36	-0.05	0.78	93	24.2	**1.10**	**0.08**	**2.12**	0.21	-0.94	1.36	0.77	-0.15	1.68
48	14.8	0.28	-0.13	0.70	-0.02	-0.10	0.06	0.37	-0.05	0.79	94	24.7	**1.18**	**0.12**	**2.24**	0.26	-0.98	1.49	0.78	-0.17	1.74
49	14.9	0.29	-0.13	0.71	-0.02	-0.12	0.08	0.38	-0.05	0.81	95	25.1	**1.26**	**0.15**	**2.36**	0.30	-1.02	1.63	0.80	-0.19	1.79
50	15.0	0.29	-0.14	0.73	-0.02	-0.14	0.09	0.38	-0.06	0.82											

Coefficients were obtained from random-effects meta-analysis and are estimated as the change in the square-root transformed outcome score at each temperature of the lag period-specific country-wide temperature distribution between percentile 5 and 95 respective to the corresponding reference temperature (10.4, 14.1, and 9.6 ºC for internalizing, externalizing, and attention problems, respectively). Bold values indicate associations that are significant at the 0.05 level and grey shaded values indicate associations that survived correction for multiple testing (P-value < 0.025). Within each INMA region, distributed lag non-linear models were adjusted for age of adolescent at and month of Child Behavioural Checklist assessment, adolescent sex, parental age, national origin, educational level, employment status, and family status; monthly income, number of children in the household, residential surrounding greenness, and neighbourhood socio-economic status. Abbreviations: CI, confidence interval; Coef., coefficient; Perc., percentile; Temp., temperature.

© 2025 Essers E et al. *JAMA Network Open*.

eTable 9, continued. Cumulative associations between ambient temperature exposure between percentiles 5 to 95 two weeks, one month, and two months prior to Child Behavioural Checklist assessment and internalizing, externalizing, or attention problems in INMA

		One-month lag period																			
		Internalizing Problems			Externalizing Problems			Attention Problems					Internalizing Problems			Externalizing Problems			Attention Problems		
Perc.	Temp. (ºC)	Coef.	95% CI		Coef.	95% CI		Coef.	95% CI		Perc.	Temp. (ºC)	Coef.	95% CI		Coef.	95% CI		Coef.	95% CI	
5	8.5	-0.12	-0.45	0.21	-0.24	-1.04	0.57	-0.02	-0.22	0.19	51	15.2	0.25	-0.26	0.75	-0.03	-0.21	0.14	0.48	-0.01	0.98
6	8.9	-0.10	-0.35	0.16	-0.20	-0.93	0.53	-0.01	-0.15	0.13	52	15.4	0.25	-0.27	0.78	-0.04	-0.24	0.16	0.50	-0.00	1.01
7	9.2	-0.08	-0.27	0.12	-0.17	-0.83	0.49	-0.01	-0.10	0.08	53	15.5	0.26	-0.28	0.81	-0.04	-0.27	0.19	**0.53**	**0.00**	**1.05**
8	9.4	-0.06	-0.20	0.09	-0.15	-0.76	0.47	-0.01	-0.05	0.04	54	15.7	0.27	-0.30	0.83	-0.05	-0.31	0.21	**0.55**	**0.01**	**1.09**
9	9.7	-0.04	-0.15	0.06	-0.13	-0.70	0.44	0.00	-0.01	0.01	55	15.9	0.28	-0.31	0.86	-0.05	-0.34	0.23	**0.57**	**0.02**	**1.13**
10	9.9	-0.03	-0.10	0.04	-0.11	-0.64	0.42	0.00	-0.02	0.03	56	16.0	0.28	-0.32	0.88	-0.06	-0.37	0.25	**0.59**	**0.02**	**1.16**
11	10.1	-0.01	-0.05	0.02	-0.09	-0.59	0.41	0.01	-0.05	0.07	57	16.2	0.29	-0.33	0.91	-0.06	-0.40	0.27	**0.61**	**0.03**	**1.19**
12	10.3	0.00	-0.00	0.00	-0.08	-0.54	0.39	0.02	-0.07	0.11	58	16.3	0.30	-0.34	0.93	-0.07	-0.43	0.29	**0.63**	**0.04**	**1.23**
13	10.5	0.01	-0.02	0.04	-0.06	-0.50	0.38	0.03	-0.08	0.14	59	16.5	0.31	-0.35	0.97	-0.08	-0.47	0.32	**0.66**	**0.04**	**1.27**
14	10.7	0.02	-0.03	0.07	-0.05	-0.47	0.36	0.03	-0.10	0.17	60	16.7	0.32	-0.36	1.00	-0.08	-0.51	0.34	**0.68**	**0.05**	**1.31**
15	10.8	0.03	-0.04	0.10	-0.04	-0.44	0.35	0.04	-0.11	0.19	61	16.9	0.33	-0.37	1.03	-0.09	-0.54	0.36	**0.70**	**0.06**	**1.35**
16	10.9	0.04	-0.05	0.13	-0.04	-0.42	0.34	0.05	-0.12	0.21	62	17.1	0.34	-0.38	1.06	-0.09	-0.57	0.38	**0.72**	**0.06**	**1.39**
17	11.1	0.05	-0.06	0.15	-0.03	-0.40	0.33	0.06	-0.12	0.23	63	17.2	0.35	-0.39	1.08	-0.10	-0.60	0.40	**0.74**	**0.07**	**1.42**
18	11.2	0.05	-0.07	0.18	-0.02	-0.38	0.33	0.06	-0.13	0.25	64	17.4	0.36	-0.40	1.11	-0.10	-0.63	0.42	**0.76**	**0.07**	**1.45**
19	11.3	0.06	-0.07	0.20	-0.02	-0.36	0.32	0.07	-0.13	0.27	65	17.5	0.37	-0.41	1.14	-0.11	-0.66	0.44	**0.78**	**0.08**	**1.49**
20	11.4	0.07	-0.08	0.22	-0.01	-0.34	0.31	0.08	-0.14	0.29	66	17.7	0.38	-0.42	1.17	-0.11	-0.69	0.47	**0.80**	**0.08**	**1.52**
21	11.5	0.07	-0.09	0.23	-0.01	-0.32	0.30	0.08	-0.14	0.31	67	17.9	0.39	-0.42	1.20	-0.11	-0.71	0.49	**0.82**	**0.08**	**1.56**
22	11.6	0.08	-0.09	0.25	-0.01	-0.31	0.30	0.09	-0.14	0.33	68	18.0	0.40	-0.43	1.22	-0.12	-0.74	0.50	**0.84**	**0.09**	**1.59**
23	11.8	0.09	-0.10	0.27	0.00	-0.29	0.29	0.10	-0.15	0.34	69	18.2	0.41	-0.44	1.25	-0.12	-0.76	0.52	**0.85**	**0.09**	**1.62**
24	11.9	0.09	-0.10	0.29	0.00	-0.28	0.28	0.11	-0.15	0.37	70	18.4	0.42	-0.44	1.28	-0.12	-0.79	0.54	**0.87**	**0.10**	**1.65**
25	12.0	0.10	-0.11	0.31	0.00	-0.26	0.27	0.12	-0.15	0.38	71	18.5	0.43	-0.44	1.30	-0.12	-0.80	0.56	**0.89**	**0.10**	**1.67**
26	12.1	0.11	-0.11	0.32	0.01	-0.24	0.26	0.13	-0.15	0.40	72	18.6	0.44	-0.44	1.32	-0.12	-0.82	0.57	**0.90**	**0.10**	**1.69**
27	12.2	0.11	-0.12	0.34	0.01	-0.23	0.25	0.14	-0.15	0.43	73	18.8	0.45	-0.44	1.34	-0.13	-0.84	0.58	**0.91**	**0.11**	**1.71**
28	12.4	0.12	-0.12	0.36	0.01	-0.21	0.24	0.15	-0.14	0.45	74	19.0	0.47	-0.44	1.38	-0.13	-0.86	0.61	**0.93**	**0.11**	**1.75**
29	12.5	0.12	-0.13	0.38	0.01	-0.20	0.22	0.16	-0.14	0.47	75	19.2	0.49	-0.44	1.41	-0.13	-0.88	0.63	**0.94**	**0.11**	**1.78**
30	12.6	0.13	-0.13	0.39	0.01	-0.18	0.21	0.17	-0.14	0.48	76	19.4	0.50	-0.44	1.44	-0.12	-0.90	0.65	**0.96**	**0.12**	**1.80**
31	12.7	0.13	-0.14	0.41	0.01	-0.17	0.20	0.18	-0.13	0.50	77	19.7	0.53	-0.43	1.48	-0.12	-0.92	0.68	**0.98**	**0.12**	**1.83**
32	12.8	0.14	-0.14	0.42	0.01	-0.16	0.19	0.20	-0.13	0.52	78	19.9	0.55	-0.42	1.52	-0.11	-0.94	0.71	**0.99**	**0.13**	**1.86**
33	12.9	0.15	-0.15	0.44	0.01	-0.14	0.17	0.21	-0.13	0.54	79	20.2	0.57	-0.41	1.55	-0.11	-0.95	0.74	**1.00**	**0.13**	**1.88**
34	13.1	0.15	-0.15	0.46	0.01	-0.13	0.15	0.22	-0.12	0.57	80	20.4	0.60	-0.39	1.59	-0.10	-0.97	0.76	**1.02**	**0.13**	**1.90**
35	13.2	0.16	-0.16	0.47	0.01	-0.11	0.14	0.24	-0.11	0.59	81	20.6	0.62	-0.38	1.62	-0.09	-0.98	0.79	**1.02**	**0.13**	**1.92**
36	13.4	0.16	-0.16	0.49	0.01	-0.09	0.12	0.25	-0.11	0.61	82	20.8	0.64	-0.36	1.65	-0.08	-0.99	0.82	**1.03**	**0.13**	**1.93**
37	13.5	0.17	-0.17	0.51	0.01	-0.07	0.10	0.27	-0.10	0.64	83	21.1	0.67	-0.34	1.68	-0.07	-1.00	0.85	**1.04**	**0.13**	**1.94**
38	13.6	0.18	-0.17	0.53	0.01	-0.06	0.08	0.28	-0.09	0.66	84	21.3	0.70	-0.32	1.72	-0.06	-1.01	0.89	**1.04**	**0.13**	**1.96**
39	13.7	0.18	-0.18	0.54	0.01	-0.05	0.06	0.30	-0.09	0.68	85	21.6	0.72	-0.30	1.75	-0.05	-1.02	0.93	**1.05**	**0.13**	**1.97**
40	13.8	0.19	-0.18	0.56	0.01	-0.03	0.04	0.31	-0.08	0.70	86	21.8	0.75	-0.29	1.78	-0.03	-1.03	0.96	**1.05**	**0.13**	**1.97**
41	14.0	0.19	-0.19	0.57	0.00	-0.02	0.02	0.32	-0.08	0.72	87	22.0	0.77	-0.27	1.82	-0.02	-1.04	1.00	**1.05**	**0.12**	**1.98**
42	14.1	0.20	-0.20	0.59	0.00	-0.00	0.01	0.34	-0.07	0.75	88	22.3	0.81	-0.24	1.86	0.00	-1.06	1.06	**1.05**	**0.11**	**1.99**
43	14.2	0.20	-0.20	0.60	0.00	-0.02	0.01	0.35	-0.06	0.77	89	22.6	0.85	-0.21	1.91	0.02	-1.08	1.12	**1.05**	**0.10**	**2.00**
44	14.3	0.21	-0.21	0.62	0.00	-0.03	0.03	0.37	-0.06	0.79	90	22.8	0.88	-0.19	1.95	0.04	-1.10	1.19	**1.04**	**0.09**	**2.00**
45	14.4	0.21	-0.21	0.64	-0.01	-0.06	0.04	0.38	-0.05	0.82	91	23.1	0.92	-0.17	2.00	0.06	-1.13	1.26	**1.04**	**0.07**	**2.01**
46	14.5	0.22	-0.22	0.65	-0.01	-0.08	0.06	0.40	-0.05	0.84	92	23.4	0.96	-0.14	2.06	0.09	-1.17	1.35	**1.03**	**0.05**	**2.02**
47	14.6	0.22	-0.23	0.67	-0.01	-0.10	0.07	0.41	-0.04	0.86	93	23.8	1.02	-0.11	2.14	0.13	-1.21	1.47	**1.02**	**0.01**	**2.03**
48	14.8	0.23	-0.24	0.69	-0.02	-0.12	0.09	0.43	-0.03	0.89	94	24.1	1.07	-0.09	2.22	0.16	-1.26	1.58	1.01	-0.03	2.04
49	14.9	0.23	-0.24	0.71	-0.02	-0.15	0.10	0.44	-0.03	0.92	95	24.5	1.12	-0.07	2.30	0.20	-1.31	1.71	0.99	-0.07	2.06
50	15.0	0.24	-0.25	0.73	-0.03	-0.17	0.12	0.46	-0.02	0.94											

Coefficients were obtained from random-effects meta-analysis and are estimated as the change in the square-root transformed outcome score at each temperature of the lag period-specific country-wide temperature distribution between percentile 5 and 95 respective to the corresponding reference temperature (10.4, 14.1, and 9.6 ºC for internalizing, externalizing, and attention problems, respectively). Bold values indicate associations that are significant at the 0.05 level and grey shaded values indicate associations that survived correction for multiple testing (P-value < 0.025). Within each INMA region, distributed lag non-linear models were adjusted for age of adolescent at and month of Child Behavioural Checklist assessment, adolescent sex, parental age, national origin, educational level, employment status, and family status; monthly income, number of children in the household, residential surrounding greenness, and neighbourhood socio-economic status. Abbreviations: CI, confidence interval; Coef., coefficient; Perc., percentile; Temp., temperature.

© 2025 Essers E et al. *JAMA Network Open*.

eTable 9, continued. Cumulative associations between ambient temperature exposure between percentiles 5 to 95 two weeks, one month, and two months prior to Child Behavioural Checklist assessment and internalizing, externalizing, or attention problems in INMA

		Two-month lag period																			
		Internalizing Problems			Externalizing Problems			Attention Problems					Internalizing Problems			Externalizing Problems			Attention Problems		
Perc.	Temp. (ºC)	Coef.	95% CI		Coef.	95% CI		Coef.	95% CI		Perc.	Temp. (ºC)	Coef.	95% CI		Coef.	95% CI		Coef.	95% CI	
5	8.0	-0.09	-0.78	0.61	-0.19	-1.45	1.08	0.03	-0.49	0.56	51	14.9	0.31	-0.32	0.94	0.14	-0.06	0.35	**0.64**	**0.02**	**1.25**
6	8.3	-0.08	-0.66	0.51	-0.22	-1.35	0.90	0.02	-0.39	0.44	52	15.1	0.32	-0.34	0.99	0.18	-0.08	0.43	**0.67**	**0.04**	**1.30**
7	8.6	-0.07	-0.55	0.42	-0.25	-1.26	0.76	0.01	-0.32	0.34	53	15.3	0.34	-0.36	1.04	0.21	-0.10	0.52	**0.71**	**0.07**	**1.35**
8	8.8	-0.06	-0.46	0.34	-0.27	-1.18	0.64	0.00	-0.24	0.25	54	15.4	0.36	-0.37	1.09	0.24	-0.12	0.60	**0.74**	**0.09**	**1.40**
9	9.1	-0.05	-0.37	0.27	-0.29	-1.11	0.53	0.00	-0.17	0.17	55	15.6	0.37	-0.39	1.14	0.28	-0.13	0.69	**0.78**	**0.11**	**1.45**
10	9.3	-0.04	-0.29	0.21	-0.31	-1.06	0.44	0.00	-0.10	0.10	56	15.8	0.39	-0.41	1.18	0.31	-0.15	0.77	**0.81**	**0.12**	**1.50**
11	9.5	-0.03	-0.22	0.15	-0.32	-1.01	0.37	0.00	-0.04	0.04	57	15.9	0.40	-0.43	1.23	0.34	-0.17	0.84	**0.84**	**0.14**	**1.55**
12	9.7	-0.02	-0.16	0.11	-0.33	-0.97	0.31	0.00	-0.01	0.01	58	16.1	0.41	-0.44	1.27	0.37	-0.18	0.92	**0.88**	**0.16**	**1.59**
13	9.9	-0.02	-0.11	0.07	-0.34	-0.94	0.26	0.00	-0.05	0.06	59	16.2	0.43	-0.46	1.32	0.40	-0.20	0.99	**0.91**	**0.17**	**1.64**
14	10.1	-0.01	-0.06	0.04	-0.34	-0.91	0.23	0.01	-0.09	0.10	60	16.4	0.44	-0.48	1.36	0.42	-0.22	1.06	**0.94**	**0.19**	**1.68**
15	10.3	0.00	-0.01	0.01	-0.35	-0.89	0.20	0.01	-0.12	0.14	61	16.5	0.46	-0.49	1.41	0.45	-0.23	1.13	**0.97**	**0.20**	**1.73**
16	10.4	0.01	-0.02	0.03	-0.35	-0.87	0.17	0.02	-0.15	0.18	62	16.7	0.47	-0.51	1.45	0.48	-0.25	1.21	**1.00**	**0.22**	**1.78**
17	10.6	0.01	-0.04	0.07	-0.35	-0.85	0.15	0.03	-0.17	0.22	63	16.9	0.49	-0.53	1.51	0.52	-0.27	1.30	**1.04**	**0.23**	**1.84**
18	10.7	0.02	-0.06	0.10	-0.35	-0.83	0.14	0.03	-0.19	0.25	64	17.1	0.51	-0.55	1.57	0.55	-0.29	1.39	**1.07**	**0.25**	**1.90**
19	10.8	0.03	-0.08	0.13	-0.35	-0.82	0.13	0.04	-0.20	0.29	65	17.3	0.52	-0.57	1.62	0.58	-0.30	1.46	**1.10**	**0.26**	**1.95**
20	11.0	0.03	-0.10	0.16	-0.34	-0.80	0.12	0.05	-0.22	0.32	66	17.5	0.54	-0.59	1.66	0.61	-0.32	1.53	**1.14**	**0.27**	**2.00**
21	11.1	0.04	-0.11	0.19	-0.34	-0.79	0.11	0.06	-0.23	0.35	67	17.6	0.56	-0.60	1.72	0.64	-0.34	1.61	**1.17**	**0.29**	**2.05**
22	11.2	0.05	-0.12	0.22	-0.33	-0.77	0.10	0.07	-0.24	0.38	68	17.9	0.58	-0.62	1.78	0.67	-0.36	1.70	**1.21**	**0.30**	**2.11**
23	11.4	0.05	-0.14	0.24	-0.33	-0.76	0.10	0.08	-0.25	0.41	69	18.0	0.59	-0.63	1.82	0.69	-0.37	1.76	**1.23**	**0.31**	**2.15**
24	11.5	0.06	-0.14	0.27	-0.32	-0.74	0.10	0.09	-0.26	0.44	70	18.2	0.61	-0.64	1.86	0.71	-0.38	1.81	**1.26**	**0.32**	**2.19**
25	11.6	0.07	-0.15	0.29	-0.32	-0.72	0.09	0.10	-0.26	0.46	71	18.3	0.62	-0.65	1.90	0.73	-0.39	1.86	**1.28**	**0.33**	**2.23**
26	11.7	0.07	-0.16	0.31	-0.31	-0.71	0.09	0.11	-0.26	0.49	72	18.5	0.64	-0.66	1.94	0.76	-0.41	1.92	**1.31**	**0.34**	**2.28**
27	11.8	0.08	-0.17	0.33	-0.30	-0.69	0.09	0.13	-0.26	0.52	73	18.7	0.66	-0.67	1.99	0.78	-0.42	1.97	**1.33**	**0.34**	**2.32**
28	11.9	0.09	-0.17	0.35	-0.29	-0.67	0.09	0.14	-0.26	0.54	74	19.0	0.68	-0.68	2.04	0.80	-0.43	2.04	**1.36**	**0.36**	**2.37**
29	12.0	0.09	-0.18	0.37	-0.28	-0.65	0.08	0.15	-0.26	0.56	75	19.2	0.70	-0.68	2.09	0.82	-0.44	2.09	**1.39**	**0.36**	**2.42**
30	12.2	0.10	-0.18	0.39	-0.27	-0.62	0.08	0.17	-0.26	0.59	76	19.5	0.73	-0.69	2.14	0.84	-0.45	2.14	**1.42**	**0.37**	**2.46**
31	12.3	0.11	-0.19	0.41	-0.26	-0.60	0.08	0.18	-0.26	0.62	77	19.7	0.75	-0.68	2.17	0.86	-0.46	2.17	**1.44**	**0.38**	**2.50**
32	12.4	0.12	-0.19	0.43	-0.25	-0.57	0.08	0.20	-0.25	0.64	78	19.9	0.77	-0.68	2.21	0.87	-0.46	2.20	**1.46**	**0.38**	**2.53**
33	12.5	0.13	-0.20	0.45	-0.23	-0.54	0.07	0.21	-0.24	0.67	79	20.2	0.79	-0.67	2.25	0.88	-0.47	2.22	**1.48**	**0.39**	**2.57**
34	12.6	0.13	-0.20	0.47	-0.22	-0.51	0.07	0.23	-0.24	0.70	80	20.4	0.81	-0.65	2.28	0.88	-0.47	2.23	**1.49**	**0.39**	**2.59**
35	12.8	0.14	-0.20	0.49	-0.20	-0.47	0.07	0.25	-0.23	0.73	81	20.7	0.84	-0.64	2.31	0.88	-0.47	2.23	**1.50**	**0.39**	**2.61**
36	12.9	0.15	-0.21	0.51	-0.19	-0.44	0.06	0.27	-0.22	0.75	82	20.9	0.86	-0.62	2.33	0.88	-0.47	2.23	**1.51**	**0.39**	**2.63**
37	13.0	0.16	-0.21	0.53	-0.17	-0.40	0.06	0.29	-0.21	0.78	83	21.1	0.87	-0.60	2.35	0.88	-0.47	2.22	**1.52**	**0.39**	**2.64**
38	13.1	0.17	-0.22	0.55	-0.15	-0.36	0.06	0.31	-0.19	0.81	84	21.3	0.89	-0.58	2.37	0.87	-0.47	2.21	**1.52**	**0.39**	**2.65**
39	13.2	0.18	-0.22	0.58	-0.14	-0.32	0.05	0.33	-0.18	0.84	85	21.5	0.91	-0.57	2.38	0.86	-0.47	2.19	**1.52**	**0.39**	**2.65**
40	13.4	0.19	-0.22	0.60	-0.12	-0.28	0.04	0.35	-0.17	0.87	86	21.7	0.92	-0.55	2.39	0.85	-0.47	2.18	**1.52**	**0.38**	**2.66**
41	13.5	0.20	-0.23	0.63	-0.10	-0.23	0.04	0.37	-0.15	0.90	87	21.9	0.94	-0.52	2.41	0.84	-0.47	2.15	**1.52**	**0.38**	**2.66**
42	13.6	0.21	-0.24	0.65	-0.08	-0.19	0.03	0.39	-0.14	0.93	88	22.2	0.97	-0.49	2.42	0.82	-0.47	2.12	**1.52**	**0.37**	**2.66**
43	13.7	0.22	-0.24	0.67	-0.06	-0.15	0.02	0.41	-0.13	0.95	89	22.4	0.99	-0.46	2.44	0.80	-0.48	2.09	**1.51**	**0.36**	**2.66**
44	13.9	0.23	-0.25	0.70	-0.04	-0.09	0.02	0.44	-0.11	0.99	90	22.7	1.01	-0.43	2.45	0.78	-0.49	2.04	**1.50**	**0.34**	**2.66**
45	14.0	0.24	-0.25	0.73	-0.02	-0.04	0.01	0.46	-0.10	1.02	91	23.0	1.04	-0.39	2.47	0.75	-0.51	2.00	**1.49**	**0.32**	**2.65**
46	14.1	0.24	-0.26	0.75	0.00	-0.00	0.00	0.48	-0.08	1.05	92	23.3	1.06	-0.35	2.48	0.71	-0.54	1.96	**1.47**	**0.30**	**2.64**
47	14.2	0.26	-0.27	0.78	0.03	-0.01	0.06	0.51	-0.06	1.08	93	23.6	1.09	-0.32	2.50	0.67	-0.57	1.92	**1.45**	**0.27**	**2.63**
48	14.4	0.27	-0.28	0.82	0.05	-0.02	0.13	0.54	-0.04	1.12	94	23.9	1.11	-0.29	2.52	0.63	-0.62	1.89	**1.43**	**0.23**	**2.62**
49	14.6	0.28	-0.29	0.86	0.08	-0.04	0.20	0.57	-0.02	1.16	95	24.3	1.14	-0.26	2.55	0.57	-0.70	1.85	**1.39**	**0.18**	**2.61**
50	14.7	0.30	-0.31	0.90	0.11	-0.05	0.27	**0.60**	**0.00**	**1.21**											

Coefficients were obtained from random-effects meta-analysis and are estimated as the change in the square-root transformed outcome score at each temperature of the lag period-specific country-wide temperature distribution between percentile 5 and 95 respective to the corresponding reference temperature (10.4, 14.1, and 9.6 ºC for internalizing, externalizing, and attention problems, respectively). Bold values indicate associations that are significant at the 0.05 level and grey shaded values indicate associations that survived correction for multiple testing (P-value < 0.025). Within each INMA region, distributed lag non-linear models were adjusted for age of adolescent at and month of Child Behavioural Checklist assessment, adolescent sex, parental age, national origin, educational level, employment status, and family status; monthly income, number of children in the household, residential surrounding greenness, and neighbourhood socio-economic status. Abbreviations: CI, confidence interval; Coef., coefficient; Perc., percentile; Temp., temperature.

© 2025 Essers E et al. *JAMA Network Open*.

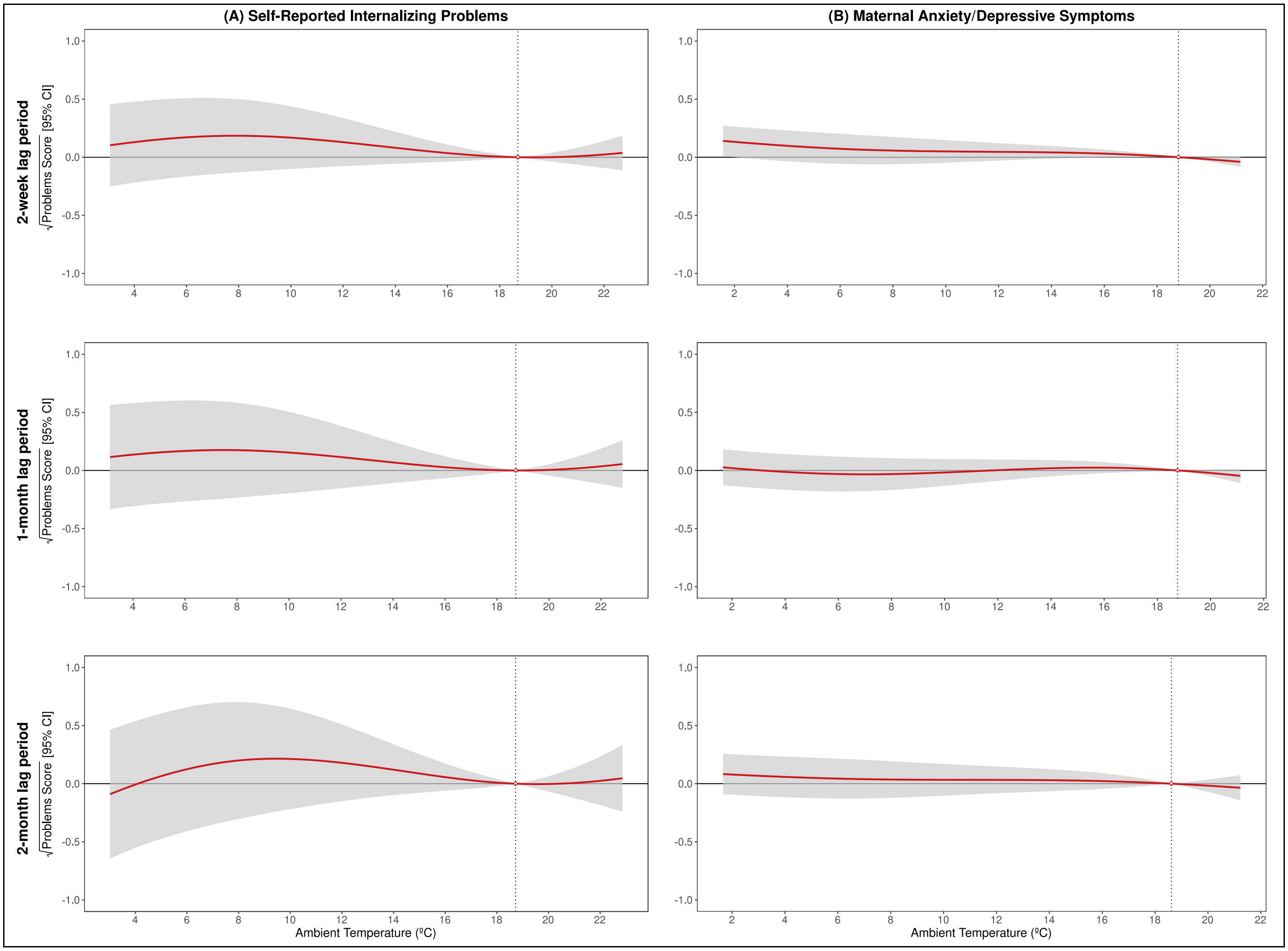

eFigure 5. Cumulative associations between temperature exposure during the two weeks, one month, and two months prior to outcome assessment and (A) self-reported internalizing problems from the YSR assessment at adolescent fourteen years (N = 4,089) and (B) maternal anxiety/depressive symptoms from the BSI assessment at child nine years (N = 3,731) in Generation R

The x-axis depicts the 5th to 95th range of the country-wide temperature distribution (for **A**, 3.1 to 22.7 ºC for the two-week, 3.1 to 22.8 ºC for the one-month, and 4.7 – 22.8 ºC for the two-month period and for **B,** 1.6 to 21.1 ºC for the two-week, 1.7 to 21.1 ºC for the one-month, and 1.7 to 21.2 ºC for the two-month period). Solid red lines represent the country-average curve, expressed as beta coefficients of the problem scores, derived from the distributed lag non-linear models, with their respective 95% confidence intervals in grey. Coefficients are estimated as the change in the outcome score at each temperature of the country-wide temperature distribution respective to the corresponding reference temperature (black dotted lines). Within each cohort, distributed lag non-linear models were adjusted for age of adolescent at and month of Child Behavioural Checklist assessment, adolescent sex, parental age, national origin, educational level, employment status, and family status; monthly income, number of children in the household, residential surrounding greenness, and neighbourhood socio-economic status. Abbreviations: BSI, Brief Symptom Inventory; CBCL, Child Behavioural Checklist; CI, confidence interval; YSR, Youth Self-Report.

© 2025 Essers E et al. *JAMA Network Open*.

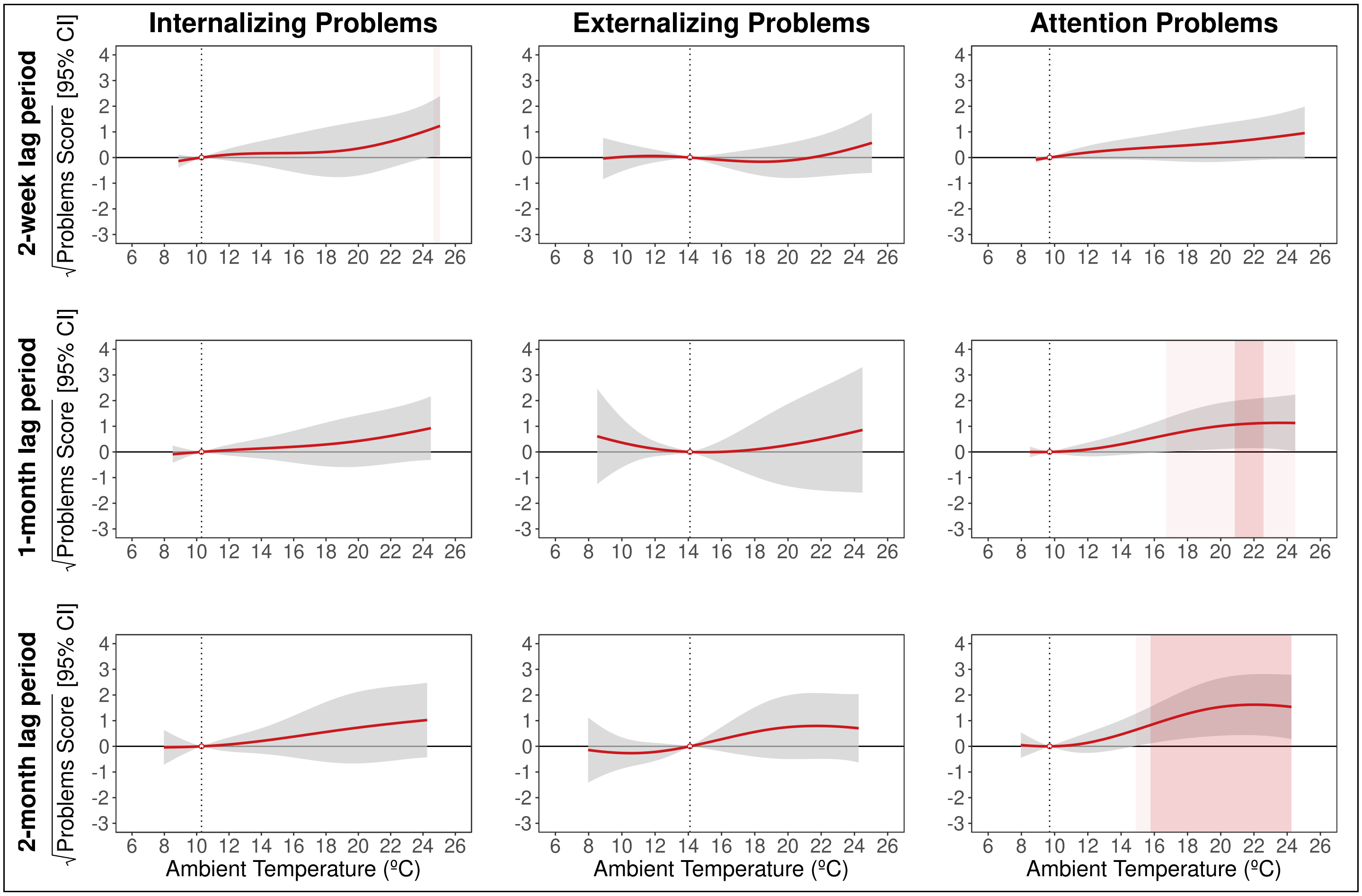

eFigure 6. Cumulative associations between temperature exposure during the two weeks, one month, or two months prior to the outcome assessment and internalizing, externalizing, or attention problems in adolescents from INMA (N = 855), additionally adjusted for the presence of air conditioning in the household.

The x-axis depicts the 5th to 95th range of the country-wide temperature distribution (8.9 – 25.0 ºC for the two weeks, 8.5 – 24.5 for the one month, and 8.0 – 24.3 ºC for the two months). Solid red lines represent the country-average curve, expressed as beta coefficients of the problem scores, derived from the random-effects meta-analysis models, with their respective 95% confidence intervals in grey. Coefficients are estimated as the change in the square-root transformed outcome score at each temperature of the country-wide temperature distribution respective to the corresponding reference temperature (black dotted lines). Light red (warmer compared to reference temperature) shaded areas indicate the range of statistically significant associations at the $P < 0.05$ level and dark red statistically significant associations after correction for multiple testing ($P < 0.025$). Within each cohort/region, distributed lag non-linear models were adjusted for the presence of air conditioning in the household, age of adolescent at and month of Child Behavioural Checklist assessment, adolescent sex, parental age, national origin, educational level, employment status, and family status; monthly income, number of children in the household, residential surrounding greenness, and neighbourhood socio-economic status. Abbreviations: CI, confidence interval.

© 2025 Essers E et al. *JAMA Network Open*.

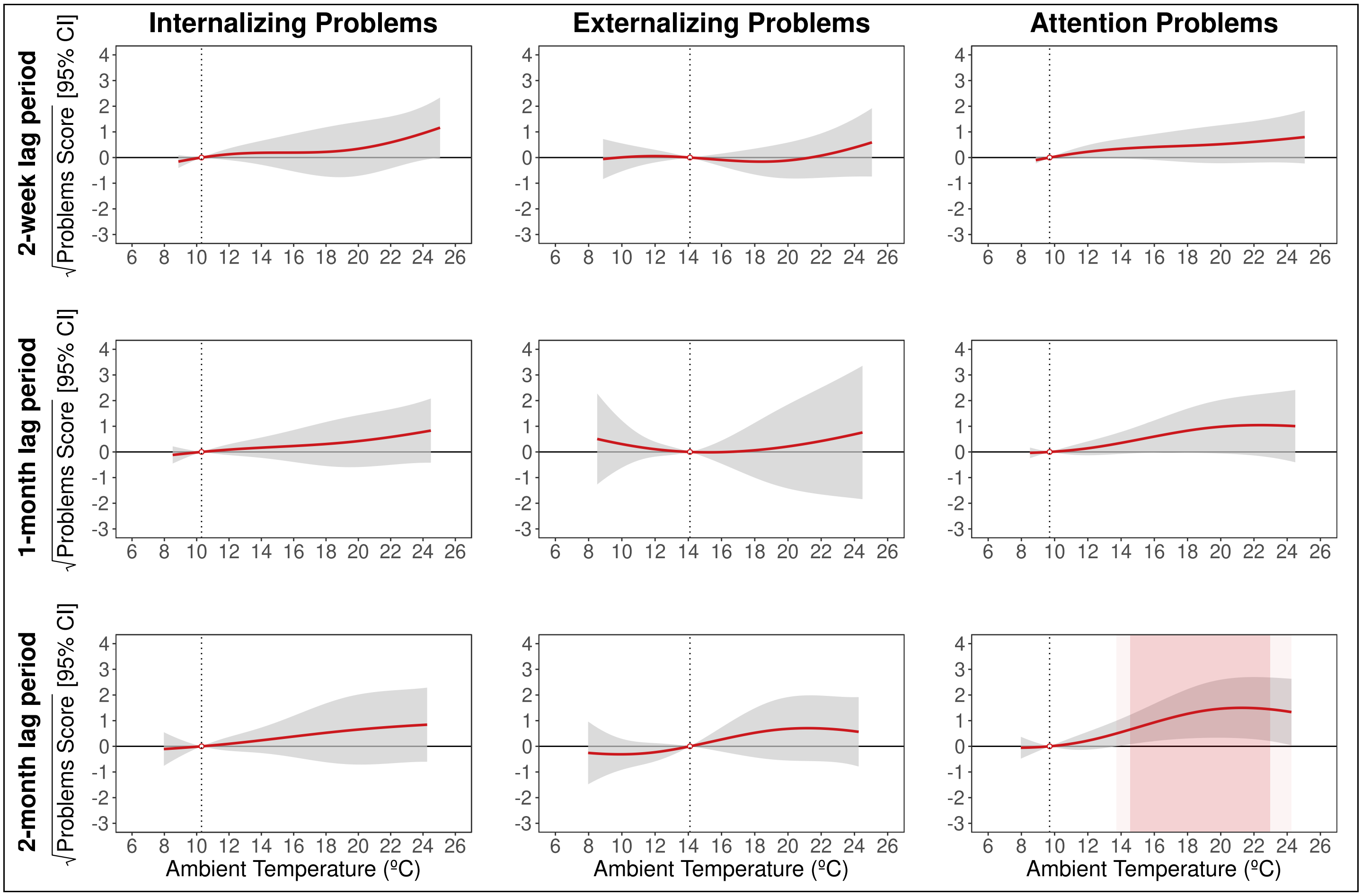

eFigure 7. Cumulative associations between temperature exposure during the two weeks, one month, or two months prior to the outcome assessment and internalizing, externalizing, or attention problems in adolescents from INMA (N = 855), additionally adjusted for the presence of heating in the household.

The x-axis depicts the 5th to 95th range of the country-wide temperature distribution (8.9 – 25.0 ºC for the two weeks, 8.5 – 24.5 for the one month, and 8.0 – 24.3 ºC for the two months). Solid red lines represent the country-average curve, expressed as beta coefficients of the problem scores, derived from the random-effects meta-analysis models, with their respective 95% confidence intervals in grey. Coefficients are estimated as the change in the square-root transformed outcome score at each temperature of the country-wide temperature distribution respective to the corresponding reference temperature (black dotted lines). Light red (warmer compared to reference temperature) shaded areas indicate the range of statistically significant associations at the *P* < 0.05 level and dark red statistically significant associations after correction for multiple testing (*P* < 0.025). Within each cohort/region, distributed lag non-linear models were adjusted for the presence of heating in the household, age of adolescent at and month of Child Behavioural Checklist assessment, adolescent sex, parental age, national origin, educational level, employment status, and family status; monthly income, number of children in the household, residential surrounding greenness, and neighbourhood socio-economic status. Abbreviations: CI, confidence interval.

© 2025 Essers E et al. *JAMA Network Open*.

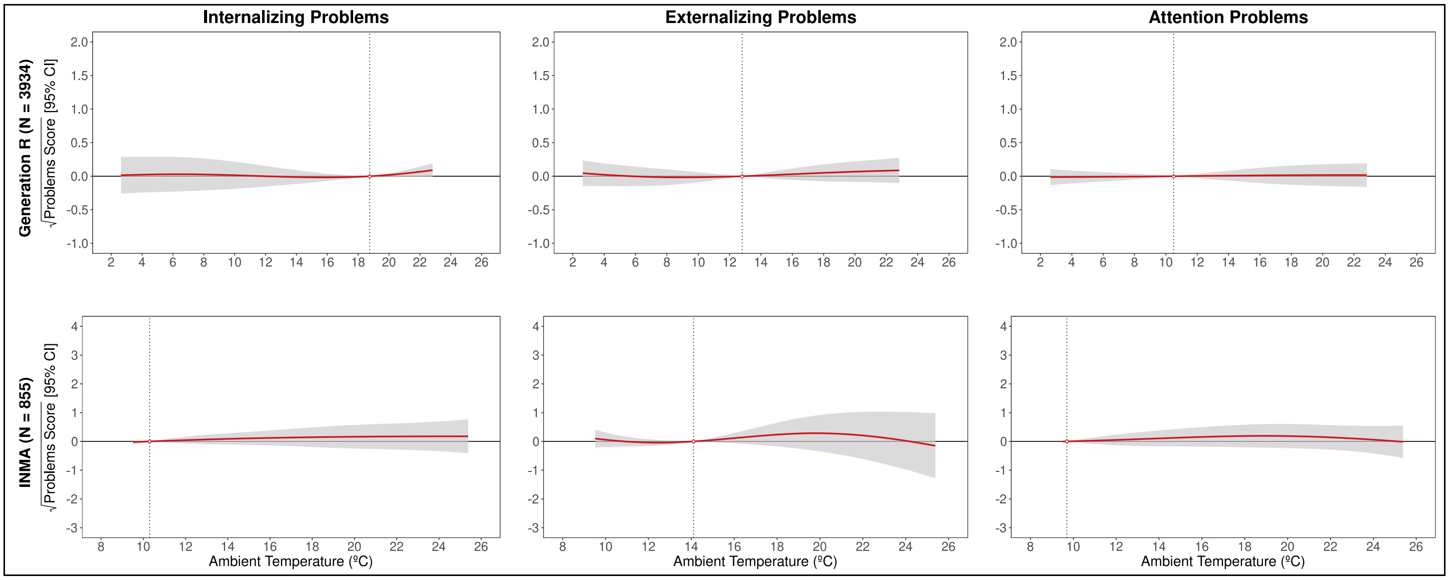

eFigure 8. Cumulative associations between temperature exposure three days prior to the outcome assessment and internalizing, externalizing, and attention problems in adolescents from Generation R (top) and INMA (bottom).

The x-axis depicts the 5th to 95th range of the country-wide temperature distribution (2.6 – 22.8 ºC in Generation R and 9.5 – 25.4 ºC in INMA). Solid red lines represent the country-average curve, expressed as beta coefficients of the problem scores, derived from the distributed lag non-linear models (Generation R) or random-effects meta-analysis models (INMA), with their respective 95% confidence intervals in grey. Coefficients are estimated as the change in the square-root transformed outcome score at each temperature of the country-wide temperature distribution respective to the corresponding reference temperature (black dotted lines). Distributed lag non-linear models were adjusted for age of adolescent at and month of Child Behavioural Checklist 6-18 assessment, adolescent sex, parental age, national origin, educational level, employment status, and family status; monthly household income, number of children in the household, residential surrounding greenness, and neighbourhood socio-economic status. Abbreviations: CI, confidence interval.

© 2025 Essers E et al. *JAMA Network Open*.

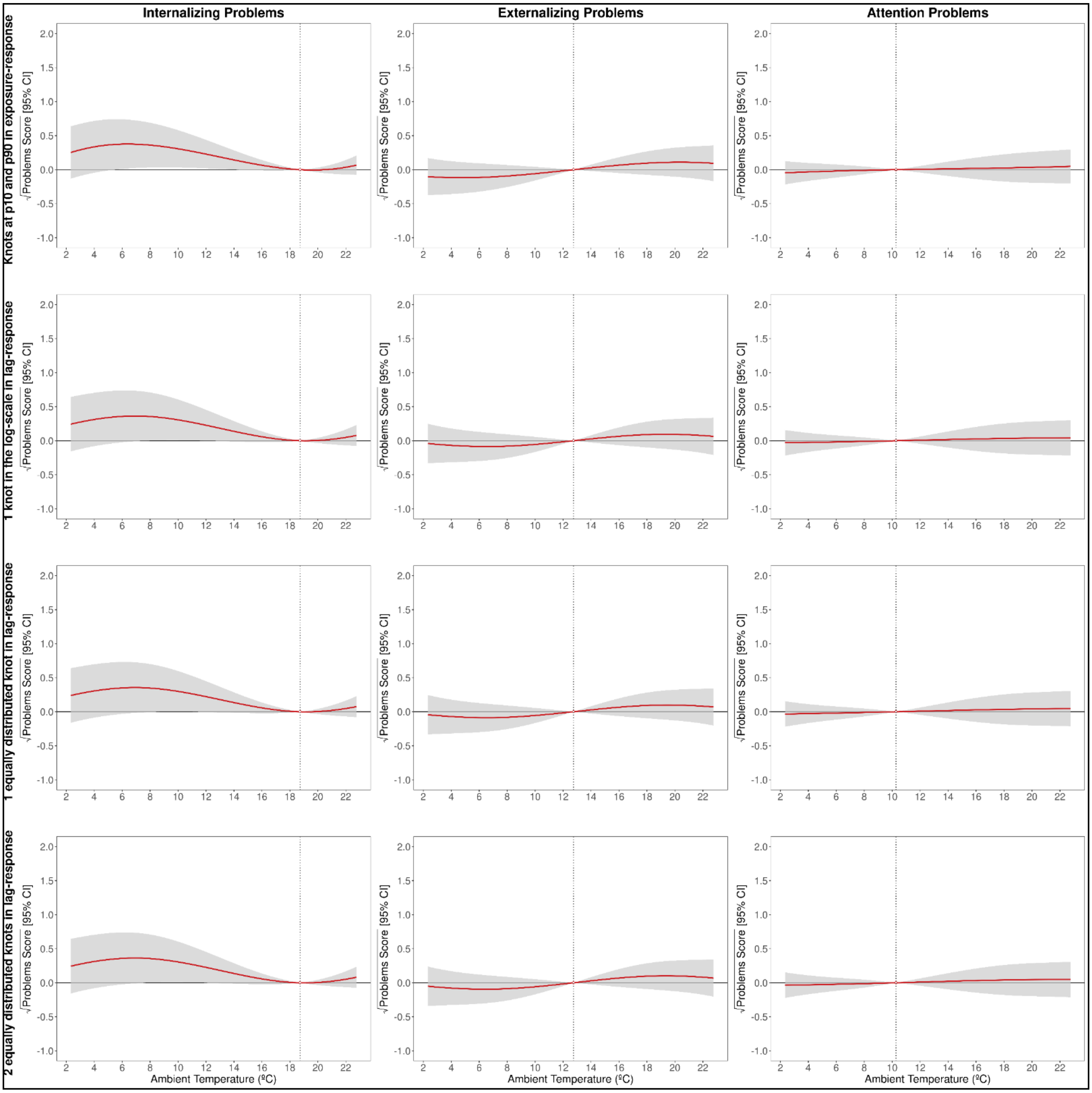

eFigure 9. Cumulative associations between temperature exposure during the **two weeks** prior to the outcome assessment and internalizing, externalizing, and attention problems in adolescents from **Generation R (N = 3,934)**, with knots placed at the 10th and 90th percentiles of temperature distribution in the exposure-response relationship (first row) or the lag-response relationship modeled with: 1 knot in the log-scale of the lag period (second row), 1 equally distributed knot in the lag period (third row), or 2 equally distributed knots in the lag period (bottom row).

The x-axis depicts the 5th to 95th range of the country-wide temperature distribution (2.3 – 22.8 ºC). Solid red lines represent the country-average curve, expressed as beta coefficients of the problem scores, derived from the cohort-specific distributed lag non-linear models, with their respective 95% confidence intervals in grey. Coefficients are estimated as the change in the outcome score at each temperature of the country-wide temperature distribution respective to the corresponding reference temperature (black dotted lines). Distributed lag non-linear models were adjusted for age of adolescent at and month of Child Behavioural Checklist 6-18 assessment, adolescent sex, parental age, national origin, educational level, employment status, and family status; monthly household income, number of children in the household, residential surrounding greenness, and neighbourhood socio-economic status. Abbreviations: CI, confidence interval; CBCL, Child Behavioural Checklist.

© 2025 Essers E et al. *JAMA Network Open*.

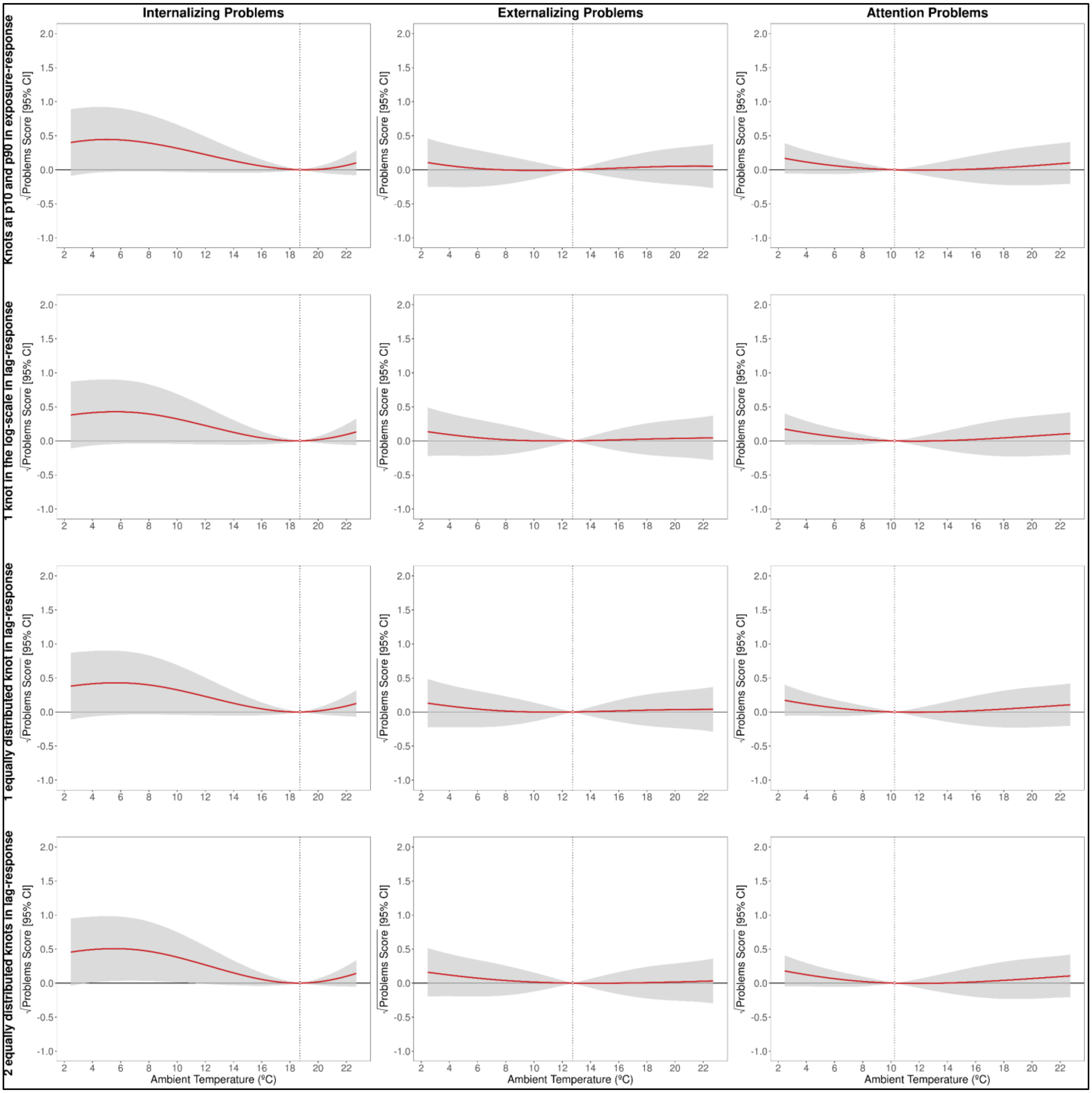

eFigure 10. Cumulative associations between temperature exposure during the **one month** prior to the outcome assessment and internalizing, externalizing, and attention problems in adolescents from **Generation R (N = 3,934)**, with knots placed at the 10th and 90th percentiles of temperature distribution in the exposure-response relationship (first row) or the lag-response relationship modeled with: 1 knot in the log-scale of the lag period (second row), 1 equally distributed knot in the lag period (third row), or 2 equally distributed knots in the lag period (bottom row).

The x-axis depicts the 5th to 95th range of the country-wide temperature distribution (2.5 – 22.7 ºC). Solid red lines represent the country-average curve, expressed as beta coefficients of the square-root problem scores, derived from the cohort-specific distributed lag non-linear models, with their respective 95% confidence intervals in grey. Coefficients are estimated as the change in the outcome score at each temperature of the country-wide temperature distribution respective to the corresponding reference temperature (black dotted lines). Distributed lag non-linear models were adjusted for age of adolescent at and month of Child Behavioural Checklist 6-18 assessment, adolescent sex, parental age, national origin, educational level, employment status, and family status; monthly household income, number of children in the household, residential surrounding greenness, and neighbourhood socio-economic status. Abbreviations: CI, confidence interval; CBCL, Child Behavioural Checklist.

© 2025 Essers E et al. *JAMA Network Open*.

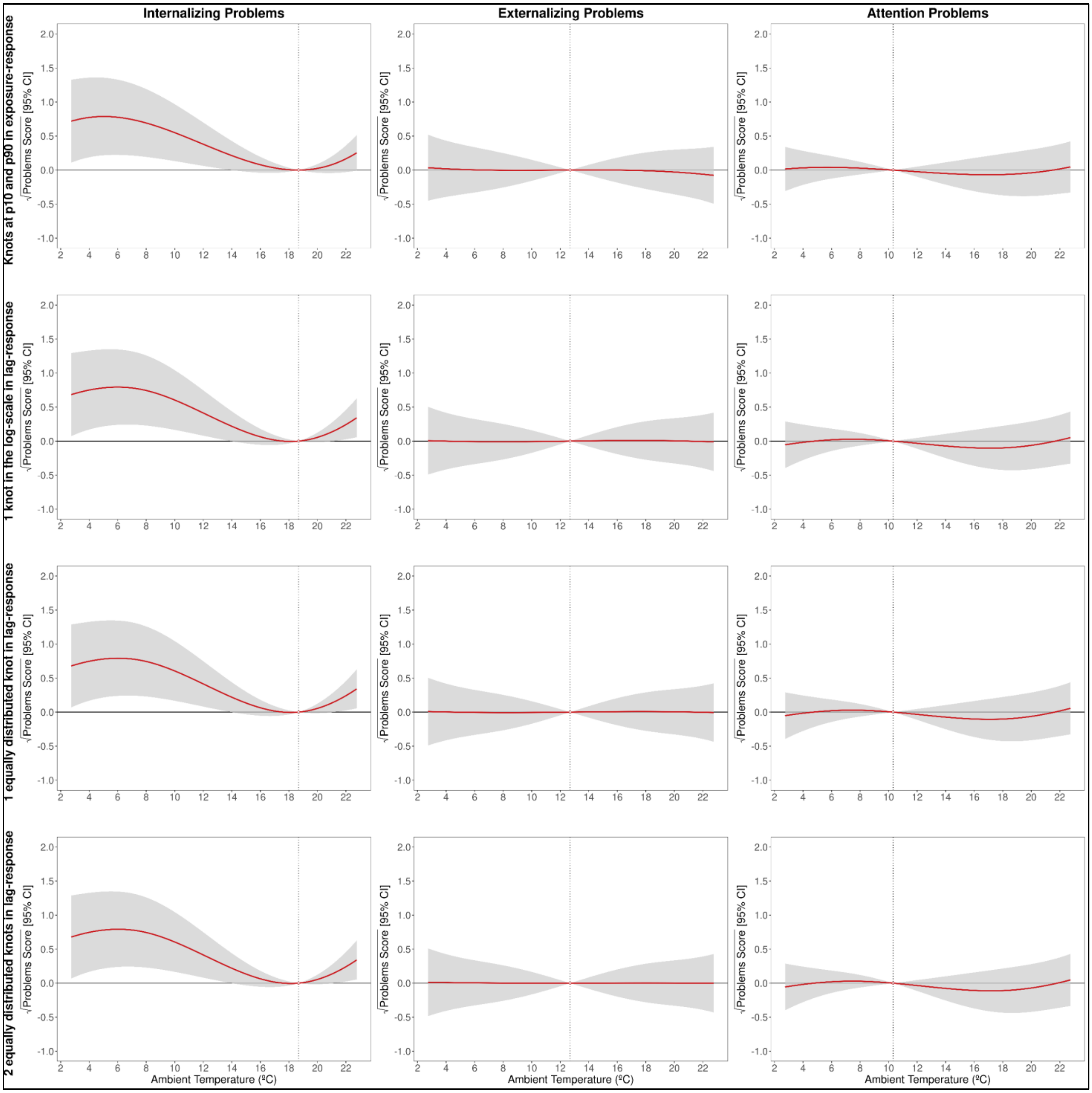

eFigure 11. Cumulative associations between temperature exposure during the **two months** prior to the outcome assessment and internalizing, externalizing, and attention problems in adolescents from **Generation R (N = 3,934)**, with knots placed at the 10th and 90th percentiles of temperature distribution in the exposure-response relationship (first row) or the lag-response relationship modeled with: 1 knot in the log-scale of the lag period (second row), 1 equally distributed knot in the lag period (third row), or 2 equally distributed knots in the lag period (bottom row).

The x-axis depicts the 5th to 95th range of the country-wide temperature distribution (2.7 – 22.8 ºC). Solid red lines represent the country-average curve, expressed as beta coefficients of the square-root problem scores, derived from the cohort-specific distributed lag non-linear models, with their respective 95% confidence intervals in grey. Coefficients are estimated as the change in the outcome score at each temperature of the country-wide temperature distribution respective to the corresponding reference temperature (black dotted lines). Distributed lag non-linear models were adjusted for age of adolescent at and month of Child Behavioural Checklist 6-18 assessment, adolescent sex, parental age, national origin, educational level, employment status, and family status; monthly household income, number of children in the household, residential surrounding greenness, and neighbourhood socio-economic status. Abbreviations: CI, confidence interval; CBCL, Child Behavioural Checklist.

© 2025 Essers E et al. *JAMA Network Open*.

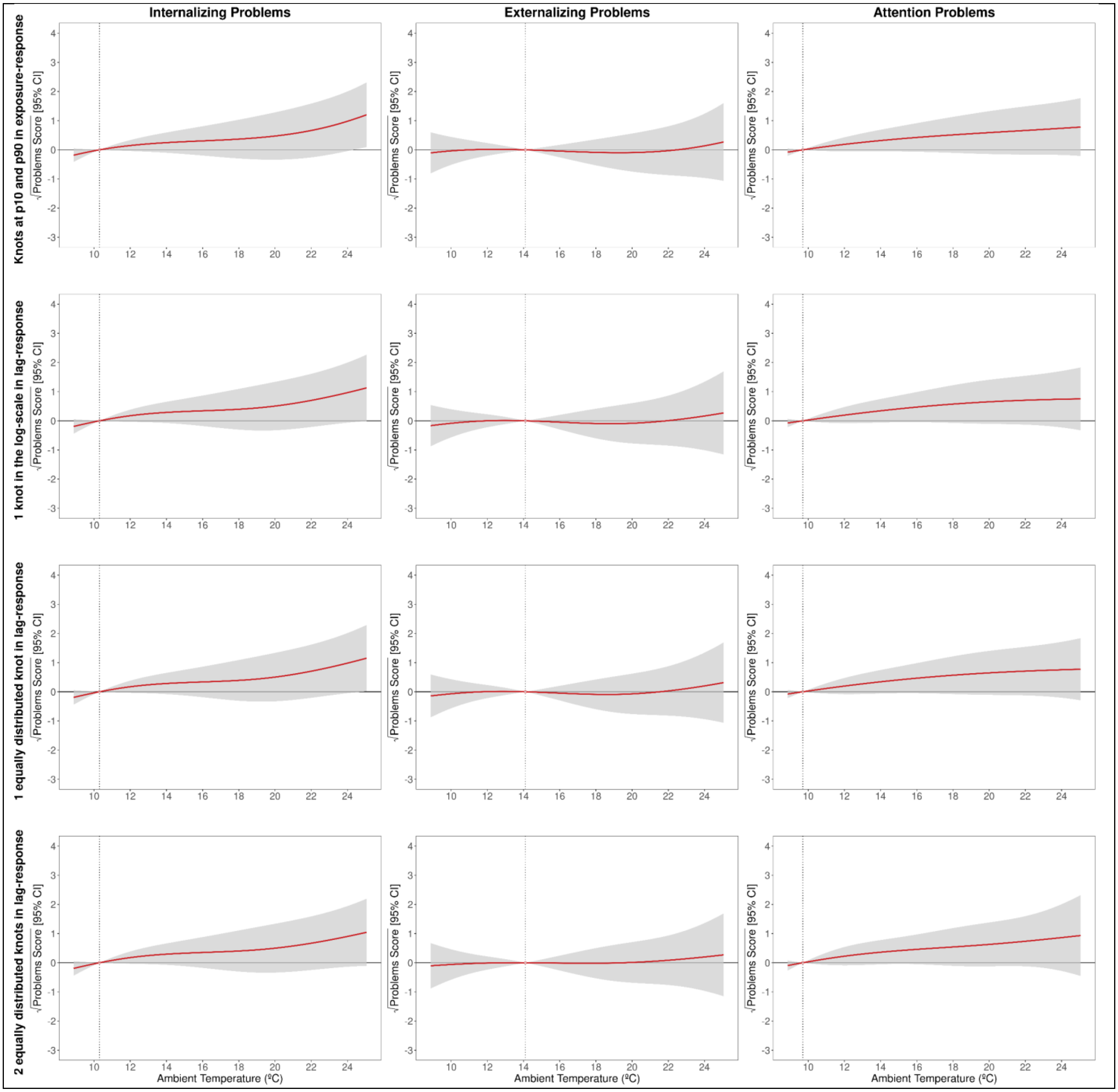

eFigure 12. Cumulative associations between temperature exposure during the **two weeks** prior to the outcome assessment and internalizing, externalizing, and attention problems in adolescents from **INMA (N = 885),** with knots placed at the 10th and 90th percentiles of temperature distribution in the exposure-response relationship (first row) or the lag-response relationship modelled with: 1 knot in the log-scale of the lag period (second row), 1 equally distributed knot in the lag period (third row), or 2 equally distributed knots in the lag period (bottom row).

The x-axis depicts the 5th to 95th range of the country-wide temperature distribution (8.9 – 25.1 ºC). Solid red lines represent the country-average curve, expressed as beta coefficients of the square-root problem scores, derived from the meta-analytical models, with their respective 95% confidence intervals in grey. Coefficients are estimated as the change in the outcome score at each temperature of the country-wide temperature distribution respective to the corresponding reference temperature (black dotted lines). Distributed lag non-linear models were adjusted for age of adolescent at and month of Child Behavioural Checklist 6-18 assessment, adolescent sex, parental age, national origin, educational level, employment status, and family status; monthly household income, number of children in the household, residential surrounding greenness, and neighbourhood socio-economic status. Abbreviations: CI, confidence interval; CBCL, Child Behavioural Checklist.

© 2025 Essers E et al. *JAMA Network Open*.

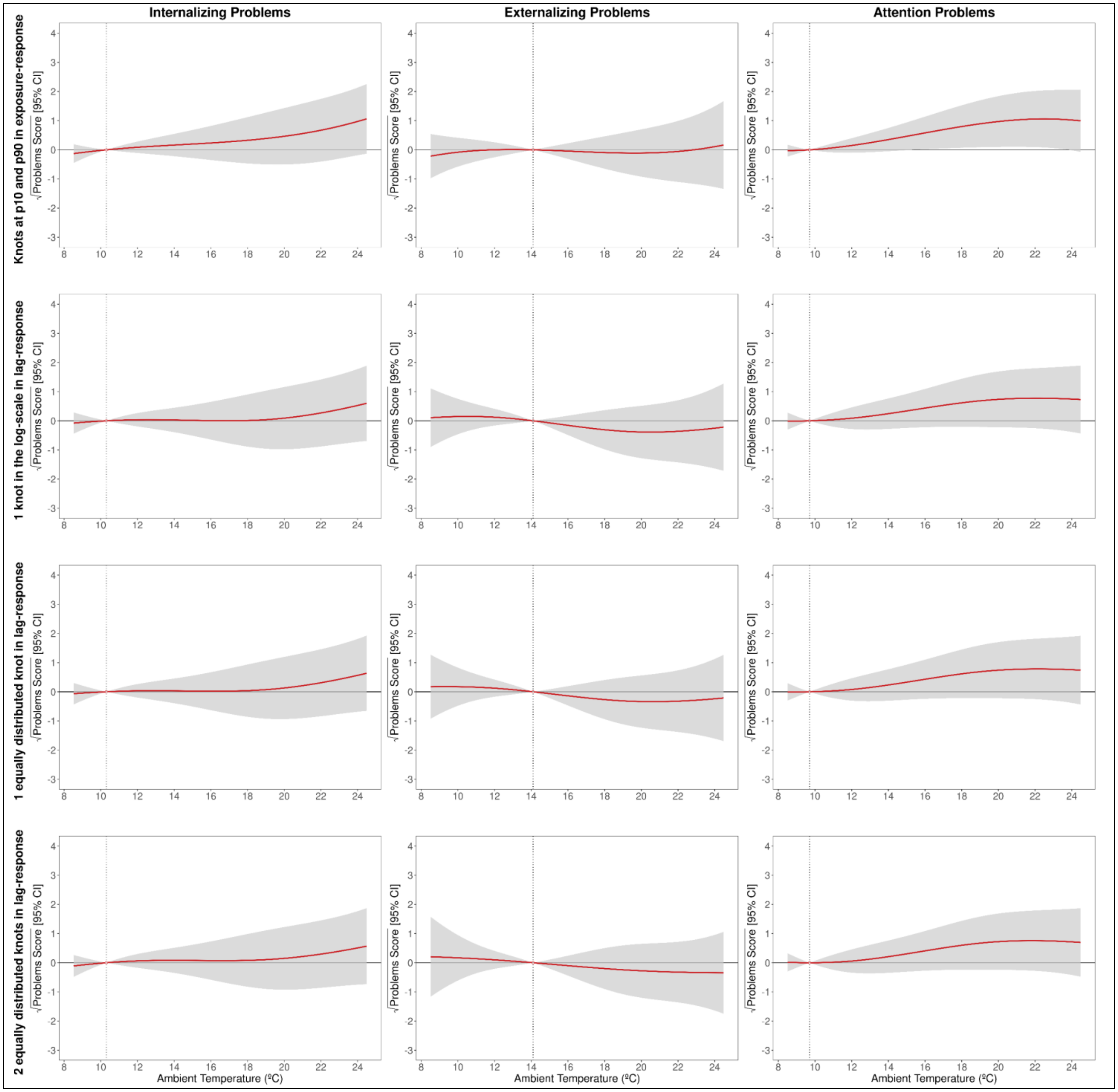

eFigure 13. Cumulative associations between temperature exposure during the **one month** prior to the outcome assessment and internalizing, externalizing, and attention problems in adolescents from **INMA (N = 885),** with knots placed at the 10th and 90th percentiles of temperature distribution in the exposure-response relationship (first row) or the lag-response relationship modeled with: 1 knot in the log-scale of the lag period (second row), 1 equally distributed knot in the lag period (third row), or 2 equally distributed knots in the lag period (bottom row).

The x-axis depicts the 5th to 95th range of the country-wide temperature distribution (8.5 – 24.5 ºC). Solid red lines represent the country-average curve, expressed as beta coefficients of the square-root problem scores, derived from the meta-analytical models, with their respective 95% confidence intervals in grey. Coefficients are estimated as the change in the outcome score at each temperature of the country-wide temperature distribution respective to the corresponding reference temperature (black dotted lines). Distributed lag non-linear models were adjusted for age of adolescent at and month of Child Behavioural Checklist 6-18 assessment, adolescent sex, parental age, national origin, educational level, employment status, and family status; monthly household income, number of children in the household, residential surrounding greenness, and neighbourhood socio-economic status. Abbreviations: CI, confidence interval; CBCL, Child Behavioural Checklist.

© 2025 Essers E et al. *JAMA Network Open*.

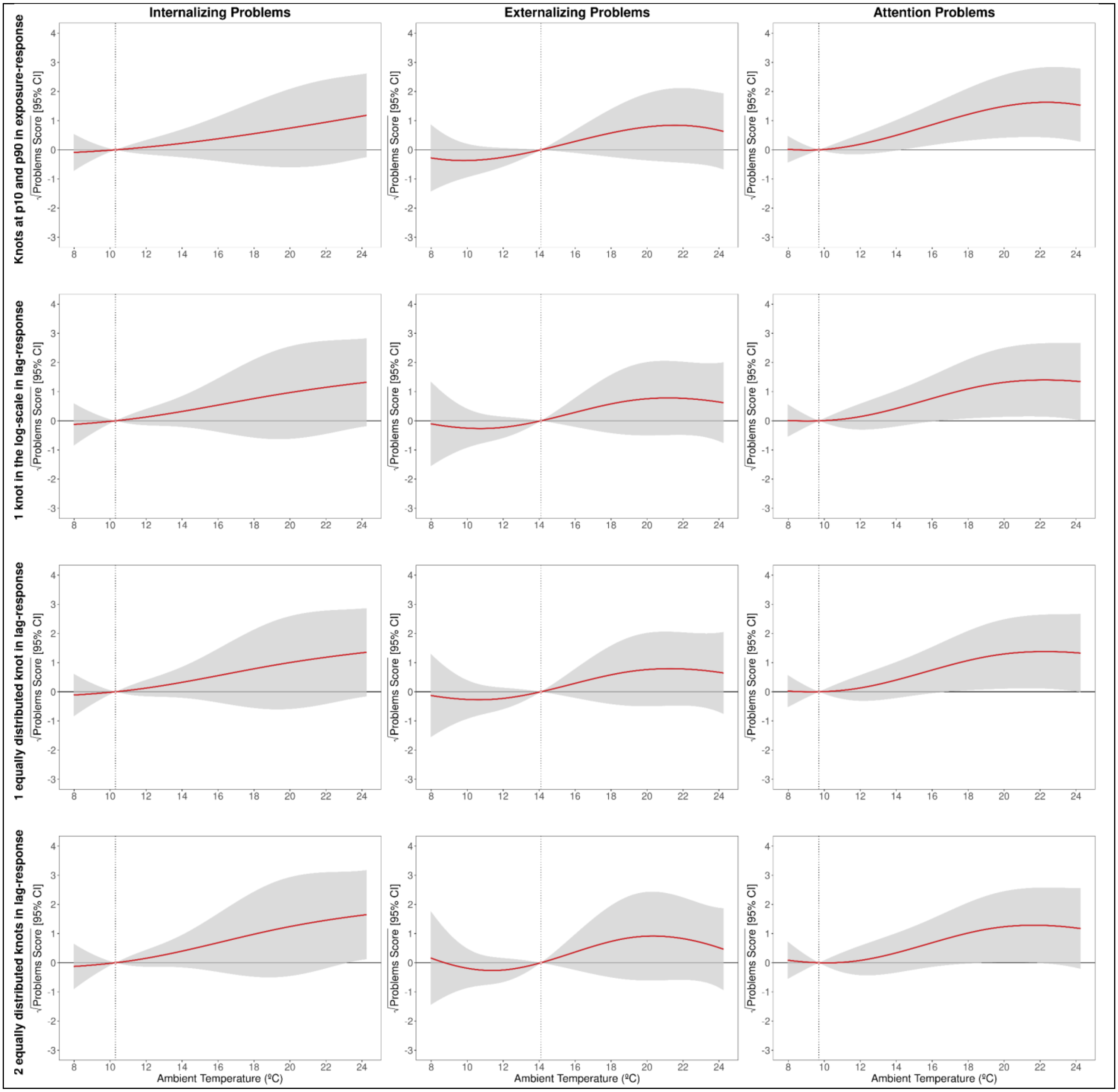

eFigure 14. Cumulative associations between temperature exposure during the **two months** prior to the outcome assessment and internalizing, externalizing, and attention problems in adolescents from **INMA (N = 885),** with knots placed at the 10th and 90th percentiles of temperature distribution in the exposure-response relationship (first row) or the lag-response relationship modeled with: 1 knot in the log-scale of the lag period (second row), 1 equally distributed knot in the lag period (third row), or 2 equally distributed knots in the lag period (bottom row).

The x-axis depicts the 5th to 95th range of the country-wide temperature distribution (8.0 – 24.3 ºC). Solid red lines represent the country-average curve, expressed as beta coefficients of the square-root problem scores, derived from the meta-analytical models, with their respective 95% confidence intervals in grey. Coefficients are estimated as the change in the outcome score at each temperature of the country-wide temperature distribution respective to the corresponding reference temperature (black dotted lines). Distributed lag non-linear models were adjusted for age of adolescent at and month of Child Behavioural Checklist 6-18 assessment, adolescent sex, parental age, national origin, educational level, employment status, and family status; monthly household income, number of children in the household, residential surrounding greenness, and neighbourhood socio-economic status. Abbreviations: CI, confidence interval; CBCL, Child Behavioural Checklist.

© 2025 Essers E et al. *JAMA Network Open*.

eReferences

1. De Ridder K, Lauwaet D, Maiheu B. UrbClim - A fast urban boundary layer climate model. *Urban Clim*. 2015;12. doi:10.1016/j.uclim.2015.01.001

2. Golan R, Kloog I, Almog R, et al. Environmental exposures and fetal growth: the Haifa pregnancy cohort study. *BMC Public Health*. 2018;18(1). doi:10.1186/s12889-018-5030-8

3. Garciá-Diéz M, Lauwaet D, Hooyberghs H, Ballester J, De Ridder K, Rodó X. Advantages of using a fast urban boundary layer model as compared to a full mesoscale model to simulate the urban heat island of Barcelona. *Geosci Model Dev*. 2016;9(12). doi:10.5194/gmd-9-4439-2016

4. Lauwaet D, Hooyberghs H, Maiheu B, et al. Detailed urban heat island projections for cities worldwide: Dynamical downscaling CMIP5 global climate models. *Climate*. 2015;3(2). doi:10.3390/cli3020391

5. Lauwaet D, De Ridder K, Saeed S, et al. Assessing the current and future urban heat island of Brussels. *Urban Clim*. 2016;15. doi:10.1016/j.uclim.2015.11.008

6. Granés L, Essers E, Ballester J, et al. Early life cold and heat exposure impacts white matter development in children. *Nat Clim Chang*. 2024;14(7):760-766. doi:10.1038/s41558-024-02027-w

7. Essers E, Granés L, Delaney S, et al. Ambient air temperature exposure and foetal size and growth in three European birth cohorts. *Environ Int*. 2024;186:108619. doi:10.1016/j.envint.2024.108619

8. Annoni A, Luzet C, Gubler E, Ihde J. *Map Projections for Europe*.; 2001.

9. Corpernicus. CORINE Land Cover. Accessed November 29, 2022. https://land.copernicus.eu/pan-european/corine-land-cover

10. SOISE. SOISE Land Cover. Accessed November 29, 2022. https://www.siose.es/presentacion

11. ECMWF. ERA5. Accessed November 29, 2022. https://www.ecmwf.int/en/forecasts/datasets/reanalysis-datasets/era5

12. NASA. MODIS Vegetation Index Products (NDVI and EVI). Accessed November 29, 2022. https://modis.gsfc.nasa.gov/data/dataprod/mod13.php

13. Honaker J, King G, Blackwell M. *AMELIA II: A Program for Missing Data, Version 1.6.2*.; 2012.

14. Gasparrini A, Armstrong B, Kenward MG. Distributed lag non-linear models. *Stat Med*. 2010;29(21). doi:10.1002/sim.3940

15. Gasparrini A. Modeling exposure-lag-response associations with distributed lag non-linear models. *Stat Med*. 2014;33(5). doi:10.1002/sim.5963

16. European Environmental Agency. Main climates of Europe. https://www.eea.europa.eu/data-and-maps/figures/climate.

17. Gasparrini A, Armstrong B, Kenward MG. Multivariate meta-analysis for non-linear and other multi-parameter associations. *Stat Med*. 2012;31(29):3821-3839. doi:10.1002/sim.5471

© 2025 Essers E et al. *JAMA Network Open*.
