# Supplementary material for: Temperature Exposure and Psychiatric Symptoms in Adolescents From 2 European Birth Cohorts
Source: JAMA Netw Open. 2025 Jan 28;8(1):e2456898. doi: 10.1001/jamanetworkopen.2024.56898 (PMC11775747; doi:10.1001/jamanetworkopen.2024.56898)
Supplement: Supplement 2. — Data Sharing Statement [file jamanetwopen-e2456898-s002.pdf]

Data Sharing Statement

Essers. Temperature Exposure and Psychiatric Symptoms in Adolescents From 2 European Birth Cohorts. *JAMA Netw Open*. Published January 28, 2025.
doi:10.1001/jamanetworkopen.2024.56898

Data
Data available: No

Additional Information
Explanation for why data not available: The datasets generated and analyzed during the current study are not publicly available due to legal and ethical regulations, but may be made available upon request to the respective directors of the cohort in accordance with the local, national, and European Union regulations: Director of the Generation R Study, Vincent Jaddoe (v.jaddoe@erasmusmc.nl) and Director of the INMA Project, Monica Guxens (monica.guxens@isglobal.org).
